# Analysing heat transport in crystalline polymers in real and reciprocal space

**DOI:** 10.1038/s41524-026-01988-0

**Published:** 2026-02-18

**Authors:** Lukas Reicht, Lukas Legenstein, Sandro Wieser, Egbert Zojer

**Affiliations:** 1 Institute of Solid State Physics, Graz University of Technology, NAWI Graz, Graz, ST Austria; 2 Institute of Materials Chemistry, TU Wien, Vienna, Austria

**Keywords:** Condensed-matter physics, Theoretical chemistry, Atomistic models, Polymers

## Abstract

Heat transport can be modelled with a variety of approaches in real space (using molecular dynamics) or in reciprocal space (using the Boltzmann transport equation). Employing two conceptually different approaches of each type, we study heat transport in crystalline polyethylene and polythiophene. We find that consistent results can be obtained when using highly efficient and accurate machine-learned potentials, provided that the physical intricacies of the considered materials and methods are correctly accounted for. For polythiophene, this turns out to be comparably straightforward, whereas for polyethylene, we find that the inclusion of higher-order anharmonicities is crucial to avoid a massive overestimation of the thermal conductivity. The responsible long-lived phonons are found at relatively high frequencies between 11 THz and 16 THz. This complicates the use of classical statistics in all molecular-dynamics-based approaches.

## Introduction

**Fig. 1:**
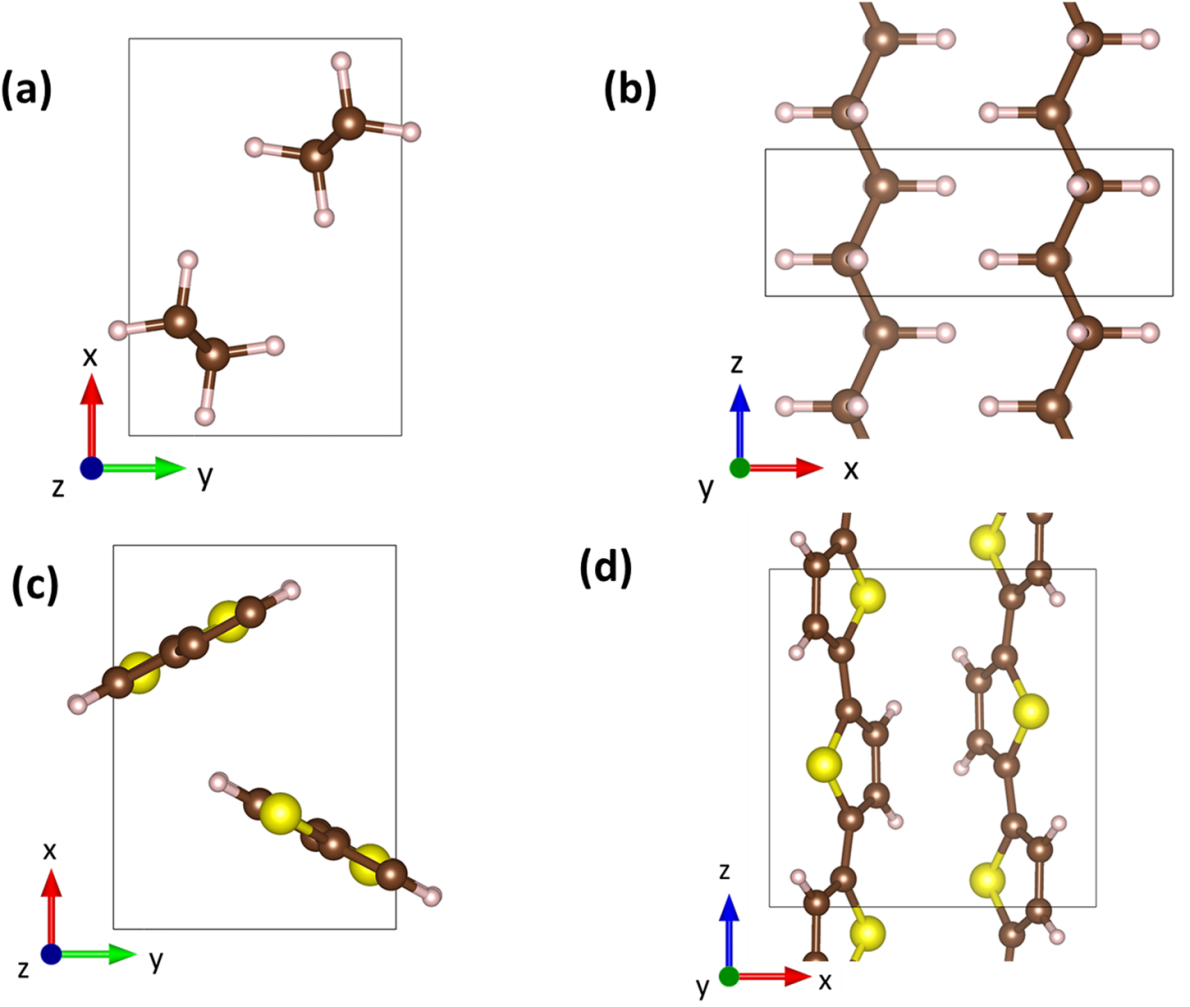
Crystal structure of polyethylene (PE) polythiophene (PT). **a** and **c** viewing direction along the polymer chains for PE and PT; **b** and **d** viewing direction perpendicular to the chains again for PE and PT. For both materials, the orthorhombic unit cell (shown with black lines) contains two molecular chains. Carbon, hydrogen, and sulphur atoms are depicted as brown, white, and yellow spheres, respectively. Adapted from ref. ^10^. © the authors, published by MDPI under CC-BY 4.0 license.

The heat-transport properties of crystalline polymers have recently attracted interest due to the potential for achieving particularly high thermal conductivities in the chain direction in highly ordered materials. In the present study, we focus on two prototypical polymeric materials, crystalline polyethylene (PE) as a model for a conventional polymer, and polythiophene (PT), as a reference material for semiconducting polymers. Their crystal structures are shown in Fig. 1. Amorphous PE and PT have low thermal conductivities of below 0.5 Wm^−1 ^K^−1^ ^1,2^. In contrast, in their crystalline state, much higher thermal conductivities can be achieved along the direction of the polymer chains. This has been demonstrated especially for PE variants, for which ref. ^3^ summarises 14 distinct measurements. Amongst these, the highest thermal conductivities of 104^−1^ ^4^ and 90 Wm^−1 ^K^−1^ ^5^ were obtained for stretched, crystalline PE fibres along the fibre axes. Also, in a variety of thin films, thermal conductivities between 22.5 and 62 Wm^−1 ^K^−1^ ^2,6–8^ were observed parallel to the direction of preferred orientation of the PE chains. Kim et al. even suggest that the ‘thermal conductivity of PE films has not yet reached its upper limit’ and that ‘the practical challenge is synthesising disentangled UHMWPE (ultra-high-molecular-weight polyethylene) films with larger extended crystal dimensions’^6^. In this context, it would be interesting to know the upper theoretical limit of PE’s thermal conductivity for a structurally perfect PE single crystal. Reliably answering this question is one of the goals of the present study.

Regarding crystalline PT, there are significantly fewer data available in the literature, but it still serves as an interesting model for conjugated materials with a fundamentally different bonding structure along the backbone. The latter comprises alternating single and double bonds between carbons, plus additional, stabilising carbon-sulphur-carbon bridges. In fact, as will become evident below, an accurate calculation of the thermal conductivity for PT is much more straightforward than for PE, which makes it a well-suited model material. For PT, we are only aware of a theoretical study, which predicts its thermal conductivity in the chain direction to be 198 Wm^−1 ^K^−1^ ^9^ (which is compared to our results in Section S2.1). This value is in a similar range as the simulated thermal conductivity along PE chains, as can be inferred from a comparison to the corresponding values in Table 1. Considering that all results mentioned above deal with transport along polymer chains and to ease the overall discussion, in the main manuscript, we focus solely on heat transport along the chains of the crystalline polymers. The results for transport in the van-der-Waals-bonded directions are provided in Sections S3 and S4.

**Table 1:** Simulated thermal conductivity of polyethylene (PE) along the chain direction, ordered by year of publication

First author	Year	Method	Scattering order	DFT functional or force field	Statistics	Thermal conductivity / Wm^−1 ^K^−1^
Zhang^56^	2020	ALD-BTE, finite differences	3	AIREBO potential	BE	216
Wang^55^	2017	ALD-BTE, finite differences	3	optB88-vdW functional^76,77^	BE	237
Shulumba^64^	2017	ALD-BTE, TDEP	3	vdW-DF functional^78^	BE MB	160 ~255
Zhang^79^	2014	NEMD	all	COMPASS	EQ	50 ^§^
Henry^52^	2010	GK	all	AIREBO potential	EQ	47 ^§^
Shen^4^	2010	GK	all	AIREBO potential	EQ	180 ± 65^§^
Ni^53^	2009	NEMD	all	REBO potential	EQ	310 ± 190

Methods are the Boltzmann transport equation based on anharmonic lattice dynamics (ALD-BTE), non-equilibrium molecular dynamics (NEMD), and Green-Kubo methods (GK). The third-order force constants for the ALD-BTE calculation can be obtained via a finite difference scheme, as it is implemented, for example, in phono3py^11^ or ShengBTE^83^, or via the temperature-dependent effective potential (TDEP)^84^ method. COMPASS^50^, AIREBO^51^ and REBO^85^ are transferable force fields. In the DFT calculations, Shulumba et al.^64^ used the van der Waals density functional by Dion et al.^78^, while Wang et al.^55^ employed the optB88-vdW^76,77^ functional. BE stands for Bose–Einstein statistics, MB for classical Maxwell–Boltzmann statistics, and EQ for classical equipartition. To date, to the best of our knowledge, no calculation exists that includes all phonon scattering orders (i.e., full anharmonicity) at the DFT accuracy level.

^§^At the time the quoted data were published, the determination of the heat flux was incorrectly implemented for many-body interactions in LAMMPS^80–82^. This likely affects the results, as such interactions are considered in the AIREBO potential and in the COMPASS force field.

A somewhat discomforting observation in Table 1 is the huge variation in the calculated thermal conductivities of PE reported in the literature. We attribute this mainly to two aspects: (i) For the description of the interatomic forces, either comparably accurate dispersion-corrected density-functional theory (DFT) approaches or classical, transferable force fields have been used. The latter are, however, notoriously inaccurate, as has recently been shown also explicitly for PE^10^. (ii) Moreover, fundamentally different methods for simulating thermal conductivities have been used in different studies. These comprise, on the one hand, reciprocal-space methods, which rely on a description of heat transport via the propagation of phonons. These methods typically employ a perturbative approach combining anharmonic lattice dynamics (ALD) and the Boltzmann Transport Equation (BTE)^11^. On the other hand, real-space approaches based on molecular dynamics (MD) have been used, like non-equilibrium molecular dynamics (NEMD)^12^ and the Green-Kubo (GK)^13,14^ method, which is based on employing equilibrium molecular dynamics simulations in combination with the fluctuation-dissipation theorem. One would hope that, even though the aforementioned approaches are fundamentally different, they yield reasonably similar results. Unfortunately, the vastly different thermal conductivities in Table 1 suggest that this might not necessarily be the case here. Such an observation would be highly problematic, as it would generally cast doubt on the reliability of heat transport simulations. One of the reasons for possible discrepancies are the commonly applied and often inevitable approximations in the MD and BTE simulations: while the former incorporate the materials’ full anharmonicity, in the BTE calculations listed in Table 1, only three-phonon scattering processes have been considered via third-order force constants. Conversely, in the BTE-based simulations, the occupation of phonon modes is described using the (appropriate) Bose–Einstein (BE) statistics, whereas all MD simulations were performed classically, such that the thermal occupation of phonons was described by the equipartition theorem (EQ)^15^. This even applies to MD simulations, where phonons are not explicitly modelled, as their properties are still implicitly reflected in the resulting MD trajectories (especially in crystalline materials).

In view of the said discrepancies, a central goal of the present paper is to assess what measures need to be taken to provide a quantitatively accurate description of thermal transport in PE and PT that works both in real space (employing MD approaches) and in reciprocal space (employing the BTE). Therefore, the above-mentioned problems need to be assessed and, if possible, overcome. In recent years, the challenge of accurately and efficiently describing interatomic interactions has been largely overcome: system-specific, machine-learned potentials (MLPs) have been shown to provide an accuracy extremely close to the parent DFT simulations, while reducing the computational costs by many orders of magnitude^16,17^. In fact, we have recently suggested an approach, in which computationally extremely efficient moment tensor potentials (MTPs) (as implemented in the MLIP code^18^) are parametrised on DFT-calculated reference structures generated during active learning runs using the VASP code^19–22^. This strategy will also be applied here and is described and benchmarked in detail in ref. ^23^. There, it is also shown that the obtained MTPs allow closely reproducing the DFT-calculated elastic constants and phonon properties of metal-organic frameworks, and that employing the MTPs in conjunction with molecular dynamics approaches yields an excellent quantitative agreement between the measured and calculated single-crystal thermal conductivity of MOF-5. Moreover, in a follow-up study, we illustrated that MTPs achieve close to DFT accuracy also when calculating phonon-related properties of polyethylene (PE), polythiophene (PT), and poly-3-hexylthiophene (P3HT)^10^. Of particular relevance for the present work is the finding that for PE, the MTPs yield results very close to those obtained with DFT, also when calculating the thermal conductivity with the BTE employing anharmonic lattice dynamics to third order (ALD-BTE)^10^. These encouraging findings show that the challenge of achieving a highly accurate and, at the same time, computationally extremely efficient description of interatomic and inter-molecular interactions can nowadays be overcome for most materials. Thus, the remaining key challenges are the intricacies of modelling thermal transport per se and doing so both based on phonon properties as well as on real-space particle trajectories.

## Results

### Brief overview of approaches for simulating thermal conductivities

Comprehensive and insightful discussions of methodological approaches for simulating heat transport go beyond the scope of the current manuscript and can, for example, be found in refs. ^15,24–26^. As discussed above, it is still crucial to address the main aspects of such simulations, as they will be relevant for the later discussion of our results on PT and PE. When aiming at a description of heat transport in reciprocal space via phonons, the most straightforward approach is to use the BTE^11^. A linearised form of the BTE can, for example, be solved directly following the approach described by Chaput et al.^27^, which is also implemented in the phono3py code^11^ (see ‘Methods section’). This direct solution of the linearised BTE is used in the current study, and we will refer to it concisely as the ‘full BTE’ solution. A common simplification of the full BTE is realised when applying the relaxation-time approximation (RTA). In this context, one has to keep in mind that employing the RTA typically leads to a reduction of the thermal conductivity^11^, an aspect that will become relevant later, when analysing BTE-calculated thermal conductivities for PE and PT. An advantage of the RTA is that it yields a particularly simple form of the BTE, which can be interpreted straightforwardly. It reads^11^

1 $${{\rm{\kappa }}}_{{RTA}}=\frac{1}{{N}_{{\boldsymbol{q}}}{V}_{c}}\mathop{\sum }\limits_{\lambda }{C}_{{\rm{\lambda }}}{{\boldsymbol{v}}}_{\lambda }\otimes {{\boldsymbol{v}}}_{\lambda }{{\rm{\tau }}}_{{\rm{\lambda }}}^{{RTA}}$$

with ${\kappa }_{{RTA}}$ representing the thermal conductivity tensor in the RTA and ${N}_{{\boldsymbol{q}}}$ denoting the number of ***q***-points used for sampling reciprocal space. The volume of the unit cell is given by ${V}_{c}$, the mode heat capacity by ${C}_{\lambda }$, the group velocity vector by ${{\boldsymbol{v}}}_{\lambda }$, and the phonon lifetime by ${\tau }_{\lambda }$. The composite index $\lambda$ refers to the wave vector ${\boldsymbol{q}}$ and the band index $n$. The sum runs over all phonon modes, $\lambda$. A particular advantage of this approach compared to, e.g., NEMD and GK is that employing Eq. (1) not only yields a numerical value of the thermal conductivity, but also allows for separately analysing the contribution of each phonon mode. The mode heat capacities ${C}_{\lambda }$ and the group velocities ${{\boldsymbol{v}}}_{\lambda }$ can be straightforwardly obtained from harmonic lattice dynamics, employing a finite-differences scheme (see ‘Methods section’). More effort is needed to determine phonon lifetimes, for which one has to consider phonon scattering processes between at least three phonons. In these anharmonic lattice dynamics (ALD-BTE) calculations, it is straightforward to apply the BE statistics as the correct quantum statistics for the occupation of the phonon modes. This affects the calculation of mode heat capacities and phonon lifetimes. Alternatively, phonon lifetimes can be extracted from equilibrium molecular dynamics trajectories (MD-BTE). In these simulations, one typically relies on classical statistics (i.e., equipartition statistics in the phonon picture^15^). When aiming at a consistent application of identical statistics in the MD-BTE calculations, one then can apply equipartition statistics also in the calculation of the mode heat capacities for evaluating Eq. (1).

Going beyond the harmonic approximation results in a shift of phonon frequencies due to anharmonic effects^28^. This can change group velocities and heat capacities. This so-called phonon renormalisation also leads to a different scattering phase space, i.e., different possibilities for phonon scattering processes that obey energy and momentum conservation. Thus, phonon renormalisation can change phonon lifetimes. For heat transport along the chain direction in PE and PT, it, however, turns out that the effects of phonon renormalisation are negligible, as shown in Section S5. In passing, we note that a perturbative approach like the BTE can only be applied as long as the phonons are not overdamped, i.e., as long as their lifetimes are sufficiently large and fulfil the Ioffe-Regel limit^29^. While the Ioffe-Regel limit is not fulfilled for strongly disordered systems^30,31^, it is satisfied for the phonons relevant in PE and PT, as shown in Section S6.

A complementary transport mechanism that is worthwhile mentioning is the conduction due to wave-like phonon tunnelling, which is neglected in the BTE. It has been described by Simoncelli et al. in the framework of the Wigner transport equation^32^. Phonon tunnelling gives rise to an additive term that increases the overall thermal conductivity. This is mainly relevant for materials with low thermal conductivity. In this context, we have recently shown that phonon tunnelling is crucial for the series of acene crystals, as prototypical examples of small-molecule-based organic semiconductors^33^. Later, Dettori et al. encountered a similar situation in 2D layered metal-organic frameworks^34^. Consistent with this notion, for the polymers considered here, phonon tunnelling becomes relevant especially in the vdW-bonded directions, as shown in Section S3. For transport along the polymer axes, where particle-like transport is highly efficient, the impact of phonon tunnelling is, however, comparably minor. More quantitatively, for PT, it increases the thermal conductivity along the chains by ca. 2.5%, while for PE, the increase is only around 0.2% (see Section S3).

When applying ALD for calculating phonon lifetimes, one often describes anharmonicities only to the leading (third-order) term, especially when dealing with highly complex materials. In the framework of the BTE, that means that only three-phonon scattering processes are taken into account for determining phonon lifetimes. The limitations of this approach will become particularly relevant for PE, making it necessary to discuss alternative approaches. Going beyond the third-order term, one can (at least conceptually) also consider higher orders, such as the fourth-order term in the Taylor expansion of the potential-energy surface. However, until recently^15,35,36^, the resulting four-phonon interactions have rarely been considered in literature, because their calculation is computationally extremely costly. As an example for a recent result, Ravichandran et al. investigated 17 zinc blende structures, where the thermal conductivities of half of them were strongly influenced by four-phonon scattering, resulting in at least a 20% reduction of the thermal conductivity at room temperature^37^. Four-phonon scattering becomes particularly important when three-phonon scattering is reduced by selection rules. This has been observed, for example, for boron arsenide, whose calculated thermal conductivity decreases from 2200 Wm^-1^K^-1^ to 1400 Wm^-1^K^-1^ when four-phonon scattering processes are included^38^. Also for graphene, four-phonon scattering has been identified as important^39^. Unfortunately, for comparably complex materials like PE and PT, an explicit calculation of four-phonon processes is computationally too demanding. Therefore, we chose to obtain phonon lifetimes beyond including only three-phonon scattering from molecular dynamics trajectories using the DynaPhoPy package^40^. In this approach, equilibrium molecular dynamics simulations are performed, during which the velocities of the atoms are tallied. These velocities are then projected onto phonon eigenvectors and onto their associated wave vectors. The autocorrelation of the group velocities is then Fourier-transformed, yielding a power spectrum for each phonon mode. Each of these power spectra consists of a single peak, which is fitted by a Lorentzian function from whose width the phonon lifetime is obtained. In this way, the full anharmonicities of the materials are considered, albeit at the price of using classical equipartition statistics. Independent of the statistics used for calculating phonon lifetimes, for the mode heat capacity, one can choose, e.g., between equipartition and the BE statistics, which leaves a variety of possible combinations. These have been analysed in detail for selected inorganic crystals by Puligheddu et al.^15^. Here, we will assess the combinations of statistics and degrees of anharmonicity that appear to be most relevant for properly describing heat transport in crystalline polymers.

Besides ALD-BTE and MD-BTE, there is also a multitude of purely MD-based techniques for describing heat transport. A widespread approach in this context is the Green-Kubo method^13,14^, where the thermal conductivity is obtained from integrating the time autocorrelation of the heat flux in an equilibrium MD calculation. As in LAMMPs a correct implementation of the heat flux for MTPs has become available only recently^41,42^, we do not apply this approach here and, instead, focus on non-equilibrium methods like the (reverse) NEMD approach^12^. In (reverse) NEMD simulations, a constant heat flux between two thermalized regions is generated by repeatedly exchanging the velocities of particles in the ‘hot’ and ‘cold’ regions, which induces a temperature gradient in the material such that the thermal conductivity can then be obtained from the ratio between the heat flux and the temperature gradient. An ambiguity in this context is caused by the fact that in many NEMD studies^43,44^ the essentially constant temperature gradient is determined in the ‘bulk’ region far from the hot and cold thermostats, and disregards the pronounced temperature drops that result from phonon scattering at the boundaries of the thermostat regions. As an alternative, Li et al.^45^ suggested that the net temperature gradient should be determined from the temperature difference and from the distance of the thermostat regions. In this way, the temperature drops at the region boundaries are also included. In the present study, the NEMD results were obtained using the strategy suggested by Li et al. because only this strategy yielded results consistent with AEMD and MD-BTE, as discussed in Section S7.

In view of the aforementioned ambiguity in the NEMD simulations, we also employed the approach-to-equilibrium molecular dynamics (AEMD)^46^ method as an alternative strategy. Here, the supercell is divided into a hot and a cold region with either a rectangular^46,47^ or a sinusoidal^48^ temperature profile. In a second step, the system is allowed to equilibrate, and the thermal conductivity of the material is obtained from the time-dependent decay of the (averaged) temperature difference of the two regions. A technical challenge in both NEMD and AEMD is that at the boundary between the different regions, additional scattering of phonons occurs. This requires a finite-size extrapolation based on calculations for a series of finite supercells with increasing size. For NEMD simulations, typically a linearp relation between the inverse thermal conductivity and the inverse supercell length is assumed. This approach has, however, been cast into doubt (see, e.g., the recent paper by Dong et al.^24^). The finite-size extrapolation for AEMD (with the thermal conductivity scaling linearly with one over the negative square root of the supercell length) has a more rigorous foundation, but is still based on some assumptions, such as a linear phonon dispersion relation^49^. Another technical challenge for NEMD and AEMD approaches is that they are computationally significantly more demanding than ALD-BTE calculations. This is particularly relevant for materials with very high thermal conductivities, which require the consideration of extremely large supercells for the finite-size extrapolations. For example, for the case of PE, the largest considered supercell contained more than 100,000 atoms, for which the coupled equations of motion were simulated for 4.3 million time steps. Historically, for complex materials like crystalline polymers, this required the use of fast empirical potentials like the COMPASS^50^ or AIREBO^51^ potential (c.f. some entries in Table 1). As these potentials are not overly accurate for predicting (anharmonic) phonon properties, it is not surprising that the four MD-based approaches in Table 1 show a vast spread in thermal conductivities of PE, ranging from 47 Wm^-1^K^-1^ ^52^ to 310 ± 190 Wm^-1^K^-1^ ^53^.

### Expected impact of methodology-inherent approximations on thermal conductivities

To conclude this discussion, it is useful to systematically summarise the impacts of the various methodological aspects, as they are particularly relevant for the remainder of the paper. This is done in Table 2, with the main effects discussed in the following.

**Table 2:** Influence of different methodological aspects on the heat capacity C_λ_, group velocity **v**_λ,_ phonon lifetime τ_λ,_ and thermal conductivity κ

BTE in the RTA				
methodological aspect	***C***_***λ***_	***v***_***λ***_	***τ***_***λ***_	***κ***
equipartition statistics instead of Bose–Einstein statistics (impacting also NEMD and AEMD simulations)	↑	—	↓ or ↑	↓ or ↑
inclusion of the fourth-order scattering rate	—	—	↓ or ↓↓	↓ or ↓↓
thermal expansion (assuming positive expansion; opposite trends would apply for negative thermal expansion)	↑	↓	↓ or ↑	typically ↓, but in specific cases ↑

effects beyond the BTE in the RTA				
full Boltzmann transport equation (i.e., disregarding the RTA)	—	—	n.a.	usually ↑

The influence is relative to the ideal situation without any approximations. Increases are indicated by ↑, decreases by ↓, and no change by —. Particularly strong increases or decreases are indicated by ↑↑ and ↓↓. For methods that do not explicitly consider C_λ_, **v**_λ_, or τ_λ_, the corresponding columns read ‘n.a.’ for ‘not applicable’. For NEMD and AEMD simulations, the impact of using equipartition statistics is expected to be qualitatively similar to solving the BTE with MD-calculated lifetimes.

**Fig. 2:**
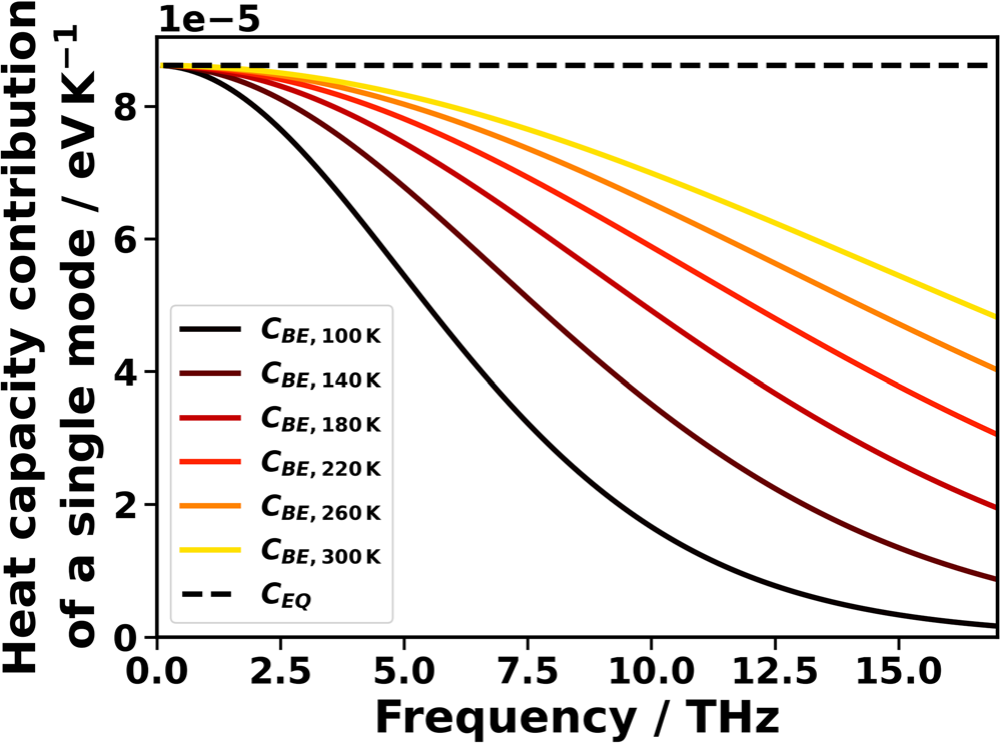
Temperature-dependent contributions to the heat capacity. Heat capacity of a single mode at a given frequency with equipartition statistics C_EQ_ (dotted line) and Bose–Einstein statistics C_BE_ for different temperatures (solid lines).

In the context of BTE-type simulations, using equipartition statistics instead of the BE distribution for calculating phonon occupations results in an overestimation of the mode heat capacity (see Fig. 2). This also leads to an overestimation of *κ*. The deviations are particularly pronounced at high frequencies and at low temperatures. These deviations also affect MD-based approaches like NEMD and AEMD, even though phonons are not considered explicitly. Moreover, when calculating phonon lifetimes from MD trajectories (MD-BTE, see above), the implicit use of equipartition statistics for phonon occupations changes the possible scattering processes and, therefore, also the phonon lifetimes, $\tau$ ^15^. In fact, Puligheddu et al. showed that (at least for MgO and PbTe) this effect dominates over the impact of equipartition statistics on the mode heat capacities^15^. Whether the use of equipartition statistics increases or decreases the phonon lifetimes can be best described for three-phonon scattering processes: for those, either a phonon decays into two other phonons, or two phonons merge into one phonon. As suggested by Turney et al., when the decay process dominates, equipartition leads to higher lifetimes, whereas when the process of merging of phonons is dominant, equipartition reduces phonon lifetimes^54^.

For particle-like transport, including fourth (and higher) order scattering rates reduces phonon lifetimes (and thermal conductivities) compared to solely considering third-order scattering. In fact, when third-order scattering is particularly low (e.g., due to selection rules), fourth-order scattering can even become dominant and lead to a drastic reduction in phonon lifetimes^38^. Notably, the probability for fourth-order scattering events tends to increase with temperature due to the larger number of available phonons^37,38^. This is opposite to the trend for the difference between equipartition and BE statistics, which drops significantly at higher temperatures. Thus, we suggest that temperature-dependent simulations of the thermal conductivity with both ALD-BTE and MD-BTE can be used to disentangle the role of the two effects, an aspect that will be exploited below.

Another process that leads to a shift of phonon frequencies is thermal expansion. Since positive thermal expansion generally decreases the bond strength, it results in a downward shift of the phonon bands. The resulting reduced phonon dispersion leads to an increase in the mode heat capacity and a decrease in the phonon group velocities, while the impact on the phonon lifetimes is a priori not certain. Typically, as a net effect, we observed a drop in the thermal conductivity when considering thermal expansion effects.

The BTE in the RTA commonly underestimates the thermal conductivity compared to the full BTE (i.e., the linearised BTE without the RTA)^55–57^. This underestimation is particularly pronounced for materials with high thermal conductivities^56^, and as such is important for PE and PT in the chain direction.

NEMD and AEMD usually employ equipartition statistics, which is expected to cause the same complications as discussed above for MD-BTE type simulations. In contrast, higher-order scattering processes are implicitly accounted for in MD-based simulations, and the RTA is not applied. Still, it is worth noting that for MgO and PbTe, Puligheddu et al. observed a very good agreement between Green-Kubo-based MD results and ALD-BTE simulations^15^. Similar observations were made in other studies^36,58,59^. Based on an in-depth analysis of the individual contributions, Puligheddu et al.^15^, however, concluded that the apparent agreement was, at least to some extent, the consequence of error-cancellation effects. Those arose from opposite impacts of various approximations on properties such as heat capacities and phonon lifetimes, as well as from applying the relaxation time approximation (RTA). Similar error-cancellation effects were also suggested by Zhou et al.^60^. Consistent with the error-cancellation argument, Puligheddu et al. observed that when phonon lifetimes are obtained from MD simulations employing the equipartition theorem, using classical statistics to compute the mode heat capacities yields thermal conductivities that agree more closely with those from ALD-BTE and Green–Kubo MD methods. As a consequence, employing the BE statistics does not serve as a simple quantum correction for simulations relying on phonon lifetimes obtained using classical MD.

These considerations raise the question, how the situation changes for crystalline polymers, which are structurally much more complex than the inorganic crystals for which the trends were summarised in the previous paragraph. Are consistent results even possible for polymers in view of all the mentioned complications? Can one still benefit from the cancellation of error effects, or are there additional shortcomings that further deteriorate the simulations? As the situation turns out to be fundamentally different in PE and PT, these two materials will be discussed separately. This will be done after providing a short description of the MLPs, which enable meaningful simulations of the types discussed above in the first place. The focus here will be on aspects necessary for understanding the results below, while further technical details can be found in the ‘Methods’ section.

### Interatomic potentials employed in the present study

Performing any of the calculations mentioned in the previous section for PT with DFT turned out to be prohibitively expensive on the available ressources^61^ when choosing numerical settings necessary for a high level of accuracy. An alternative strategy is the use of highly accurate and efficient MLPs, which are trained on a few hundred reference configurations of relatively small supercells. In particular, we used MTPs^18^. These were trained on DFT-calculated reference configurations produced in an MD-based active learning run^21,22^ that follows a predefined temperature ramp, as implemented in the VASP code^19,20^. The key aspects of this approach are summarised in the ‘Methods’ section and described in detail in ref. ^23^. There, it is also shown that the resulting MTPs are numerically extremely efficient, while at the same time they provide a close to DFT-quality description of several physical observables, particularly in metal-organic frameworks^23^. The same applies to PE and PT, as shown in ref. ^10^. In the latter study, we also extended the strategy by explicitly parametrising optimal potentials for the desired use case. For ALD-BTE simulations, interatomic force constants are calculated from structures in which individual atoms or pairs of atoms are displaced only slightly. The resulting phonon properties are highly sensitive to the accuracy of the calculated forces^62^. Therefore, the ideal potentials for ALD simulations, referred to as MTP^phonon^, comprise highly complex variants of MTPs (expressed by choosing a high value of the ‘level’ parameter^18^) parametrised on reference structures generated only at low temperatures (up to 100 K; to consider only comparably small atomic displacements in the parametrisation). The achieved high level of accuracy comes at the price of a somewhat decreased computational efficiency. Although the number of required force calculations is large, especially when calculating phonon lifetimes, it is still much smaller than when performing MD simulations including finite-size corrections. Therefore, for MD simulations, numerically more efficient (i.e., lower ‘level’) MTPs, referred to as MTP^MD^, need to be trained. This needs to be done on reference structures containing also large displacements, as in MD runs at room temperature also more strongly distorted structures are visited. Here, reference structures were generated during active learning runs up to 500 K. As discussed in ref. ^10^, the two types of MTPs are not simply ‘interchangeable’; i.e., it is typically not advisable to use an MTP^phonon^ for MD calculations at room temperature, as this will result in a disintegration of the studied materials during the MD run; also MTP^MD^ type potentials are not necessarily ideal for calculating phonon properties as they can potentially yield imaginary phonon bands. All these aspects have been considered for the simulations described in the following. Additionally, to further optimise the performance of the MTPs, we used potentials trained on unit cells with lattice parameters adapted to the 300 K and alternatively to the DFT-relaxed structures, as detailed in the Methods Section. In passing, we note that, ideally, the same MTP^MD^s would be used for the MD-BTE calculations, on the one hand, and for the NEMD and AEMD simulations, on the other hand. Due to technical reasons described in Section S11, the potentials for the NEMD and AEMD runs were, however, not fully consistent, but (as also illustrated in Section S11) this has only a very minor impact on the results.

As a final aspect, it needs to be stressed that the parametrisation of MTPs is a stochastic process. Thus, we always parametrised several (typically five) MTPs, from which the ‘best’ were selected based on their performance in either reproducing phonon band structures or in predicting forces occurring in molecular dynamics runs (see Methods section for details). To provide a sense for the spread between the MTPs, we present not only the data obtained with the ‘best’ MTPs, but also those calculated with all other MTPs. Additionally, for certain observables, we report values averaged over all MTPs together with the calculated standard deviations.

### Polythiophene: very good agreement between ALD-BTE, MD-BTE, and fully MD-based methods

With accurate MTPs at hand, we perform ALD-BTE calculations of the thermal conductivity of PT along the chain direction at 300 K. This is done using the 300 K unit cell as well as the DFT-optimised cell. The former has been obtained from an MD simulation at 300 K as described in the Methods section, while the latter is the outcome of a conventional, gradient-based geometry optimisation and, thus, disregards the impact of finite temperature on the unit-cell dimensions. As the gradient-based optimisations using DFT and MTPs yield very similar results (see ref. ^10^. and Section S12), the comparison of the thermal conductivities obtained with the 300 K cell and with the DFT-relaxed cell illustrates the impact of thermal expansion. Performing these simulations within the RTA actually allows one to assess the impact on specific phonons. When employing the ‘best’ MTP^phonon^, the ALD-BTE simulation within the RTA performed for polythiophene yields a rather sizable value of 84 Wm^−1 ^K^−1^ for the DFT optimised unit cell and a slightly smaller value of 79 Wm^−1 ^K^−1^ for the 300 K cell. As shown in Table 3, values similar to those for the ‘best’ MTPs are obtained when considering the mean values of all five parametrised MTPs. Also, the standard deviations when considering all parametrised MTPs are rather small in both cases, which testifies to the consistency of the results.

**Table 3:** Thermal conductivity of crystalline PT at 300 K along the chain direction calculated with different methods

Method	Cell	Thermal conductivity / Wm^−1 ^K^−1^
ALD-BTE in RTA	DFT-relaxed	‘best’: 84 mean: 81 [ ± 5]
full ALD-BTE	DFT-relaxed	‘best’: 98 mean: 96 [ ± 5]
ALD-BTE in RTA	300 K	‘best’: 79 mean: 71 [ ± 6]
full ALD-BTE	300 K	‘best’: **92** mean: 85 [ ± 7]
MD-BTE($(C_{BE})$)	300 K	70
MD-BTE($({C}_{EQ})$)	300 K	83
MD-BTE($\left({C}_{{{EQ}}},\frac{{{{\kappa }}}_{{{fullBTE}}}}{\kappa_{RTA}}\right)$)	300 K	**97**
NEMD	300 K	Linear fit: **84** ± 9 2nd order fit: 98 ± 12
AEMD	300 K	**94** ± 3

The methods are the linearised Boltzmann transport equation building on anharmonic lattice dynamics (ALD-BTE) in the relaxation time approximation (RTA) and without that approximation (full ALD-BTE), non-equilibrium molecular dynamics (NEMD), approach-to-equilibrium-molecular dynamics (AEMD), and the Boltzmann transport equation with lifetimes from molecular dynamics (MD-BTE). For ALD-BTE calculations, the values in square brackets refer to the standard deviation of the thermal conductivities calculated for five independently parametrised MTP^phonon^s. Uncertainties in NEMD and AEMD results are reported as standard deviations (68% confidence) derived from the finite-size extrapolation (see Sections S8 and S10 for details). Heat capacities are treated according to the Bose–Einstein (BE) distribution, C_BE_, or following equipartition statistics (EQ), C_EQ_. In ALD-BTE calculations, lifetimes are treated with Bose–Einstein statistics and the phonon scatterings are taken into account up to third order, while in MD-BTE, NEMD, and AEMD calculations, lifetimes follow equipartition statistics and the full anharmonicity is considered. The ratio between the full ALD-BTE and the ALD-BTE in the RTA is 91 Wm^−1 ^K^−1^ divided by 79 Wm^−1 ^K^−1^. This ratio is used to scale the thermal conductivity of MD-BTE, resulting in MD-BTE(${C}_{{EQ}},\frac{{\kappa }_{{fullBTE}}}{{\kappa }_{{RTA}}}$). Values intended for a direct comparison between the methods are highlighted in bold.

As discussed above, the RTA usually underestimates the thermal conductivity of materials with high thermal conductivities^55,56^ such as PT and PE. Therefore, we also solved the linearised BTE directly^11,27^, referring to the result as ‘full ALD-BTE’. As expected, the full ALD-BTE yields somewhat higher thermal conductivities of 98 Wm^−1 ^K^−1^ for the DFT-optimised unit cell and 92 Wm^−1 ^K^-1^ for the 300 K unit cell. While this approach generally leads to more accurate results, it does not rely on well-defined relaxation times for each mode, which makes a mode-resolved analysis impossible. Considering that for PT the discrepancy between the RTA and the full ALD-BTE results is not particularly large, it is, thus, useful to analyse the contributions of individual modes to the thermal conductivity on the basis of the RTA simulations.

**Fig. 3:**
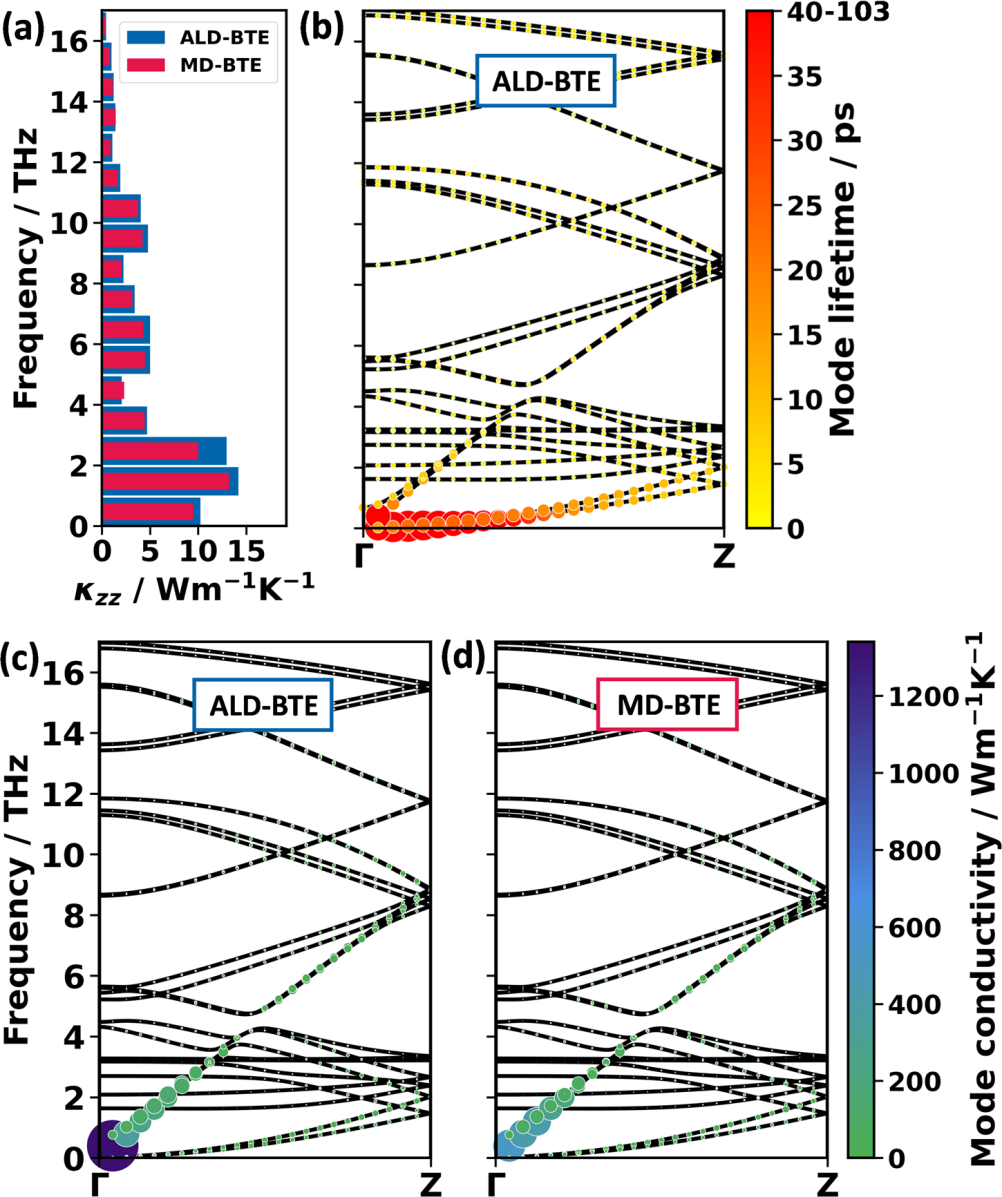
Mode contributions to the thermal conductivity in PT. **a** Histogram of thermal conductivity contributions, ${\kappa }_{{zz}}$, of PT in intervals of 1 THz. The bars are calculated as a sum over the entire Brillouin zone, sampled with a 4 × 6 × 48 (ALD-BTE) and a 2 × 3 × 48 (MD-BTE) **q**-mesh. **b** Shows the lifetimes along the chain direction from the same ALD-BTE calculation. A respective plot for the MD-BTE calculation can be found in Section S15. The additional panels illustrate the thermal conductivity contributions of the phonon modes for the (**c**) ALD-BTE and (**d**) MD-BTE calculations. The areas of the spheres and their colours are scaled linearly with the lifetime and thermal conductivity contribution. Here, the MD-BTE calculation is computed using C_BE_, which gives a qualitatively similar result compared to using C_EQ_ (see Section S14). Both calculations are performed for the 300 K unit cell with the MTPs specified in the ‘Methods’ section.

Adding up the thermal conductivity contributions in windows of 1 THz yields the results shown in Fig. 3a by blue bars. Together with the cumulative thermal conductivity shown in Fig. S3, they illustrate that more than half of the contribution to the thermal conductivity comes from phonons below 4 THz. In fact, it is a common observation that low-frequency phonons are the primary carriers of heat^63^, because of the rapid decrease of phonon lifetimes with frequency. This drop in phonon lifetime is found also for PT, as illustrated by the phonon lifetimes superimposed on the phonon band structures in Fig. 3b. The largest phonon lifetimes are found for the longitudinal acoustic band at low frequencies and for the two transverse acoustic phonon bands. When considering the mode-resolved contributions to the thermal conductivity in Fig. 3c, one, however, sees that the mode contributions of the two transverse acoustic bands are very small. This can be attributed to the very small group velocities of the associated phonons, following from the particularly small slopes of the bands (c.f., Eq. (1)). As a result, the longitudinal acoustic phonons close to the Γ-point clearly have the largest mode contributions. Nevertheless, as the reciprocal space volume close to the Γ-point is rather small and increases with the wavevector, ***q***, the bin with the largest mode-contribution to the thermal conductivity in Fig. 3a is the one between 1 THz and 2 THz. The bands between 4 and 12 THz are also comparably steep, which results in often sizable group velocities of the phonons, especially at large ***q***-values (see Fig. 3a). This is responsible for the non-negligible mode contributions to the thermal conductivity in that frequency range, despite the rather small lifetimes of the respective phonons.

A further advantage of the mode-resolved analysis, when explicitly considering phonon lifetimes, is that it allows investigating the role of higher-order scatterings by comparing the ALD-BTE calculation (Fig. 3a, c) with MD-BTE results (Fig. 3a, d). A possible origin for the somewhat larger deviation of the MD-BTE calculation at the point closest to Γ is discussed in Section S13. As argued in the Section dealing with the ‘Expected impact of methodology-inherent approximations on thermal conductivities’, differences between ALD-BTE and MD-BTE primarily arise from the inclusion, respectively, the exclusion of higher-order phonon scattering effects, and also from the use of different phonon occupation statistics. The latter can directly influence the results via the dependence of the mode heat capacities on phonon occupation. Additionally (as described in the Section dealing with the ‘Expected impact of methodology-inherent approximations on thermal conductivities’), the used phonon statistics also impact the results indirectly via the modification of the phonon scattering rates. Nevertheless, only minor differences are observed between ALD-BTE and MD-BTE calculated mode contributions across the entire frequency range. This is illustrated in Fig. 3a and is also seen when comparing Fig. 3c, d.

A priori, one cannot exclude that effects due to higher-order scattering and due to the use of classical statistics in MD-BTE simulations might cancel. However, this is very unlikely here, as the impact of employing equipartition instead of the BE statistics should distinctly increase with frequency; i.e., the difference between *C*_*EQ*_ and *C*_*BE*_ is larger for higher frequencies (see Fig. 2 and Section S14 for an explicit calculation with both heat capacities). A corresponding increase in the deviations between ALD-BTE and MD-BTE calculated phonon contributions to the thermal conductivity is, however, not observed (see Fig. 3a). There is also no indication for a pronounced impact of the occupation statistics on phonon lifetimes. Thus, the data in Fig. 3a suggest that higher-order phonon scattering processes do not play a significant role in PT. This is consistent with the observation that in PT in the low-frequency region, phonon bands lie densely, ensuring a dominance of third-order scattering without particularly strong restrictions due to selection rules^37^.

As indicated above, when evaluating the BTE with MD-extracted lifetimes (MD-BTE), choosing either *C*_*EQ*_ or *C*_*BE*_ in the evaluation of Eq. (1) does not qualitatively change the situation (see also Section S14). The appeal of using C_EQ_ would be that consistent occupation statistics would be used in the evaluation of the mode heat capacities, and when extracting phonon lifetimes from MD trajectories. Conversely, when using *C*_*BE*_, the physically correct occupation statistics would be applied at least for the evaluation of the mode heat capacities. In practice, the thermal conductivities are rather similar in both cases: the MD-BTE(${C}_{{BE}}$) approach yields 70 Wm^−1 ^K^−1^, while MD-BTE(${C}_{{EQ}}$) yields 83 Wm^−1 ^K^−1^. Both values agree well with the ALD-BTE result when also using the RTA (76 Wm^−1 ^K^−1^). In passing, we note that Puligheddu et al. observed a better agreement between MD-BTE and ALD-BTE results when using equipartition statistics for MD-BTE, an aspect they attributed to error cancellations^15^. Such a clear trend is not seen in our simulations on PT. A final aspect relevant in the context of the MD-BTE calculations is that we are not aware of a strategy to seamlessly integrate the MD calculated phonon lifetimes into a direct solution of the BTE, going beyond the relaxation time approximation. Therefore, we rescaled the MD-BTE(${C}_{{EQ}}$) value with the ratio between the aforementioned ALD-BTE results for full BTE simulations and when applying the RTA. This approach is rather approximate, but in the present context it still provides a rough estimate. It yields a value of 97 Wm^−1 ^K^−1^ for the rescaled MD-BTE(${C}_{{EQ}}$, $\frac{{\kappa }_{{fullBTE}}}{{\kappa }_{{RTA}}}$) result (see Table 3).

The good agreement between the different simulations discussed so far raises the question of whether purely MD-based approaches would also provide consistent results. To address this question, we performed NEMD and AEMD calculations (now employing an MTP of the type described in Section S11). In analogy to MD-BTE, both NEMD and AEMD are based on equipartition statistics and capture phonon scatterings to all orders (i.e., they consider the full level of anharmonicity). Additionally, NEMD and AEMD implicitly include phonon renormalisation and phonon tunnelling effects, even though these effects are small for PT along the chain direction (see above and Sections S3 and S5). The NEMD and AEMD results are listed in Table 3. They amount to 84 Wm^−1 ^K^−1^ ± 9 Wm^−1 ^K^−1^ (NEMD; linear fit), and 94 Wm^−1 ^K^−1^ ± 3 Wm^−1 ^K^−1^ (AEMD). Both values agree very well with the full ALD-BTE result and with the MD-BTE result rescaled to correct for shortcomings of the RTA (bold values in Table 3).

Regarding the uncertainty estimates of the NEMD and AEMD simulations, three aspects need to be considered: (i) the values in Table 3 are estimated fitting errors from the finite size extrapolation (see Sections S8 and S10) and, consequently, describe the situation for a single MTP. (ii) To additionally estimate the spread of MTPs with differently initialised parameters, we performed AEMD simulations on the smallest considered supercells with each of those MTPs (see Section S16). This yielded an uncertainty estimate of about 8%. This value is comparable to (iii) the inherent statistical uncertainty of an AEMD simulation, which we determined by repeating AEMD simulations multiple times with the same MTP, but changing the random seed for initialising the velocities in AEMD simulations (see Section S16).

Overall, the very good agreement between the full BTE result, the rescaled MD-BTE(C_EQ_) data, as well as the NEMD and the AEMD calculations is highly encouraging. It shows that consistent results can be obtained independent of the used methodology, as long as all relevant technical peculiarities are considered and as long as the level of anharmonicity included in the different approaches is sufficient. This promising finding shows that it can also be worthwhile for other materials to perform a simultaneous analysis of thermal transport processes in reciprocal space (i.e., via phonon properties and corresponding transport equations) and in real space (i.e., from particle trajectories).

### Polyethylene: Why MD-based methods give a lower thermal conductivity than ALD-BTE simulations relying on third-order force constants

The situation becomes considerably more complex for PE despite its seemingly simpler structure: On the one hand, certain technical complications are encountered, which will be discussed first. On the other hand, due to the suppression of three-phonon scattering processes by selection rules, it becomes necessary to go beyond merely calculating third-order force constants when aiming at a consistent description of heat transport in real and reciprocal space.

Regarding technical aspects, a distinct advantage of PE is that its structure is simple enough that third-order force constants and phonon lifetimes can be calculated using DFT with properly converged numerical settings (see ‘Methods’ section). This is feasible because simulations for PE require only about 8000 single-point calculations, whereas the more complex PT would require around 50,000 calculations^10^. Consequently, in the following discussion, the MTP results can be directly compared with DFT data. As shown in ref. ^10^, MTPs exhibited an excellent performance for a variety of observables when applied for DFT- and MTP-optimised unit cells obtained with the conjugate gradient algorithm^10^. This notion is also confirmed by the data in the second and third row of Table 4, which shows essentially identical thermal conductivities for the ALD-BTE approach when using DFT or the ‘best’ MTPs. The corresponding values are 296 Wm^−1 ^K^−1^ (DFT) and 295 Wm^−1 ^K^−1^ (‘best’ MTP^phonon^) when applying the RTA and 398 Wm^−1 ^K^−1^ (DFT) and 408 Wm^−1 ^K^−1^ (‘best’ MTP^phonon^) when directly solving the linearised BTE. These results show that the thermal conductivity along the chain direction is extremely high. They also illustrate once more that for a high thermal conductivity material like crystalline PE, applying the RTA results in severely underestimated thermal conductivities. Also, when averaging over all parametrised MTPs, similar values of 292 [ ± 33] Wm^−1 ^K^−1^ (RTA) and 388 [ ± 40] Wm^−1 ^K^−1^ (full BTE) are obtained, where the values in square brackets again refer to the standard deviation of the results for five parametrised MTPs. In passing, we note that the rather large statistical error is primarily caused by a single MTP that reproduces the general trends but produces a comparably small value of the thermal conductivity, as discussed in Sections S18 and S19.

**Table 4:** Thermal conductivity of PE along the chain direction calculated with different variants of the ALD-BTE method and different unit cells

Method	Unit cell	*κ*^DFT^ / Wm^−1^K^−1^	*κ*^MTP^ / Wm^−1^ K^−1^
ALD-BTE, RTA	DFT-relaxed	296	‘best’: 295 (mean: 284 [ ± 34])
ALD-BTE, full	DFT-relaxed	398	‘best’: 408 (mean: 388 [ ± 40])
ALD-BTE, RTA	300 K	**242**	‘best’: 263 (mean: 324 [ ± 41])
ALD-BTE, full	300 K	**309**	‘best’: 393 (mean: not meaningful^§^)

DFT-calculated thermal conductivities are denoted by κ^DFT^, while MTP-calculated values are denoted by κ^MTP^. Five independent MTPs are parametrised, of which we report the ‘best’ (see ‘Methods’ section for definition of ‘best’), the mean, and the standard deviation (in square brackets). The DFT values with the 300 K unit cell are used in the subsequent comparison to the MD-based approaches and are therefore highlighted in bold.

^§^One of the five MTPs yielded a clearly incorrect thermal conductivity, rendering the calculation of a mean value not meaningful, as discussed in more detail in Section S18.

When considering the 300 K unit cell, the thermal conductivity is reduced, as was the case in PT. The effect is, however, significantly more pronounced in PE, for which *κ* drops by between 18 and 29% in the DFT-based ALD-BTE simulations, resulting in values of 242 Wm^−1 ^K^−1^ (RTA) and 309 Wm^−1 ^K^−1^ (full BTE). This larger drop can be attributed to the more significant thermal expansion of PE compared to PT (for example, the volume changes by 6.6% for PE and only by 1.9% in PT between the DFT-optimised and the 300 K cells).

Also, in the calculations performed with the ‘best’ MTP^phonon^, a drop of *κ* to 263 Wm^−1 ^K^−1^ (in the RTA) and to 393 Wm^−1 ^K^−1^ (for the full BTE) is observed. This drop is, however, much less pronounced than in the DFT simulations; more problematic is the observation that this trend is not obeyed for all MTPs, such that the mean value for the five parametrised MTPs even increases to 324 Wm^−1 ^K^−1^ when applying the RTA. Even worse, when solving the full BTE for all tested ***q***-meshes, one finds that one specific MTP consistently yields unrealistically high thermal conductivity values, which in this case makes averaging futile (see Section S18). The origin of these problems with the MTPs trained for the 300 K unit cell of PE remains elusive, as they are not observed in any other case we have ever studied (PT above, PE with DFT-optimised cell, molecular crystals in ref. ^33^, and metal-organic frameworks in ref. ^23^, etc.). The problems also prevail despite numerous tests (including the repeated generation of training data, varying the displacement amplitude in phono3py between 0.01 and 0.11 Å, and varying the **q**-mesh). In this context, it is also worth noting that no such inconsistencies occur in the MD-BTE simulations, where the thermal conductivities calculated with the five independently parametrised MTP^MD^s for the 300 K unit cell display a standard deviation of only 7%. These five MD-BTE calculations also give qualitatively similar mode-resolved contributions (see Section S17). Likewise, in the AEMD simulations, the MTP^MD^s display a benign behaviour with a standard deviation of 5% between the five independent MTP^MD^s (see Section S16). In view of these observations, we tentatively attribute the complications in the full ALD-BTE simulations of PE in the 300 K unit cell to numerical instabilities, adversely impacting third-order force constants in this specific case.

As a consequence, in all following comparisons, we will consider the DFT-calculated data whenever referring to the ALD-BTE results for PE. As already mentioned above, the complications discussed in the current paragraph are not relevant for any of the MD-based approaches discussed next, which is good news, as none of these calculations could be reasonably done using DFT.

Notably, the aforementioned technical complications are not the only unusual observation in which PE fundamentally differs from PT. Even more importantly, when comparing the ALD-BTE to the MD-BTE results (employing the Bose–Einstein statistics for the mode heat capacities), the thermal conductivities drop by a factor of around three to 95 Wm^−1 ^K^−1^ for the DFT-optimised unit cell and to 78 Wm^−1 ^K^−1^ for the 300 K cell. The drop is somewhat less pronounced (but still dramatic) when employing the equipartition theorem, with values of 135 Wm^−1 ^K^−1^ and 114 Wm^−1 ^K^−1^, respectively.

This raises the question of whether the ALD-BTE or the MD-BTE values are more appropriate. Additionally, it is a priori unclear whether the massive differences between ALD-BTE and MD-BTE results in PE are caused by the inclusion of higher-order phonon scattering or by the use of different phonon-occupation statistics (or by both).

**Fig. 4:**
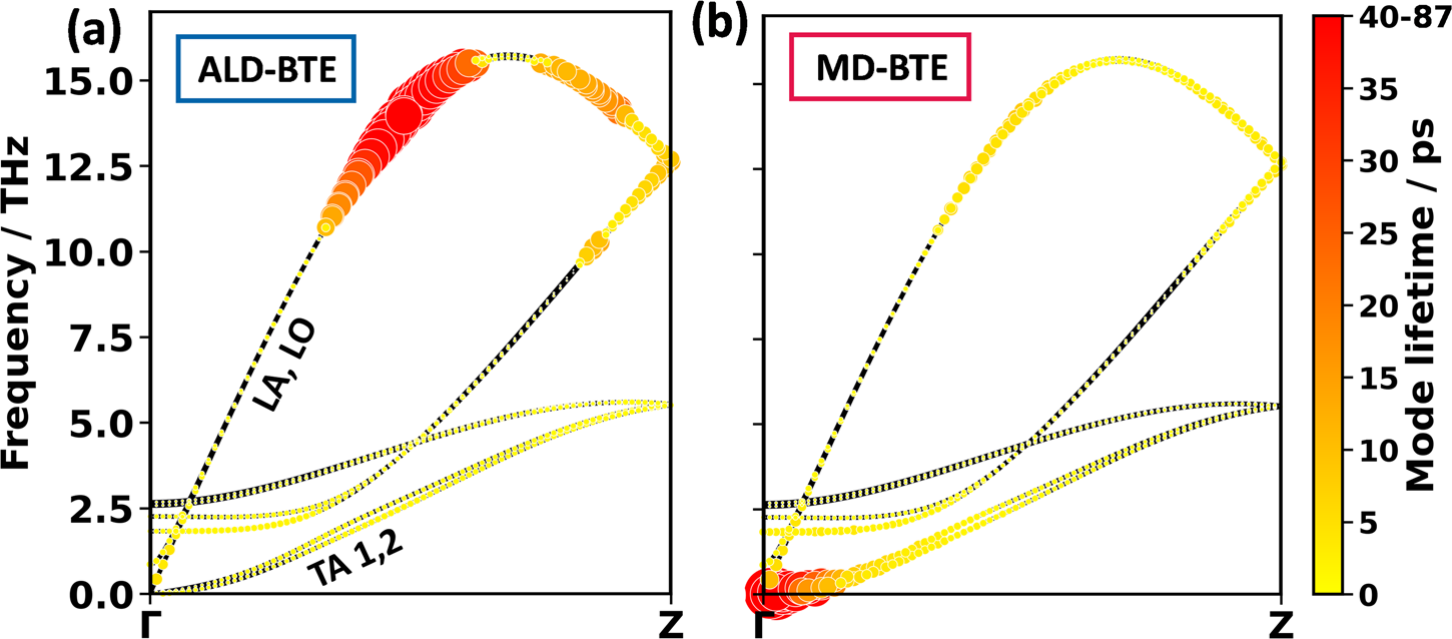
Phonon lifetimes in PE. Phonon band structure of PE along the chain direction with superimposed spheres, whose areas and colours are scaled linearly with the phonon lifetime. The displayed data have been calculated for the 300 K unit cell. **a** Shows the ALD-BTE calculation performed with DFT. **b** Is the MD-BTE calculation performed with the MTP. The longitudinal acoustic (LA), longitudinal optical (LO), and the two transverse acoustic (TA) bands are indicated. In passing, we note that for the MD-BTE simulations, the TA modes close to the Γ−point display particularly large phonon lifetimes. As shown in Fig. 5, these modes are, however, insignificant for the thermal conductivities, as they display very small phonon group velocities.

**Fig. 5:**
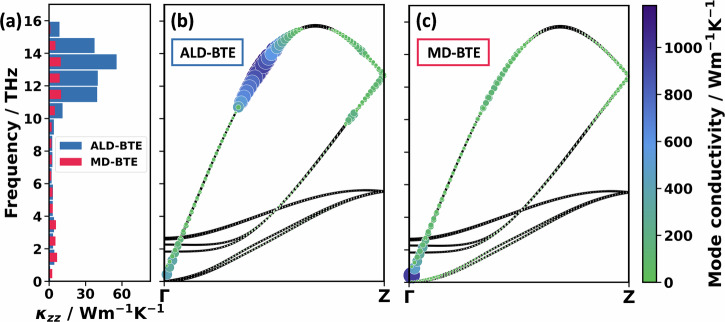
Mode contributions to the thermal conductivity in PE. **a** Histogram of thermal conductivity contributions, ${\kappa }_{{zz}}$, of PE in intervals of 1 THz. The bars are calculated as a sum over the entire Brillouin zone, sampled with a 4 × 6 × 160 (ALD-BTE) and 2 × 3 × 160 (MD-BTE) **q**-mesh. Further panels provide the thermal conductivity contribution of the phonon modes for the (**b**) ALD-BTE and (**c**) MD-BTE calculations. The areas of the spheres and their colours are scaled linearly with the lifetime and thermal conductivity contribution. Smaller spheres are plotted in front such that they are not occluded. Calculations are performed with the Bose–Einstein heat capacity and the 300 K unit cell.

To address these questions, it is useful to first discuss the phonon lifetimes of PE. The values calculated from the third-order force constants (c.f., ALD-BTE) are superimposed on the phonon band-structures for wavevectors parallel to the polymer chains in Fig. 4a. The equivalent plot for phonon lifetimes obtained from MD trajectories (c.f., MD-BTE) is provided in Fig. 4b. Surprisingly, when considering only three-phonon scattering processes (Fig. 4a) by far the highest lifetimes are found in the frequency range between 11 and 16 THz, while low-frequency phonons exhibit lifetimes that are more than an order of magnitude smaller. This atypical behaviour has already been observed by Wang et al.^55^, for a single PE chain. They explained it by certain (usually dominant) three-phonon scattering channels being unavailable for the phonons in the said frequency range: typically, the dominant scattering mechanism affecting longitudinal acoustic modes in PE is Umklapp scattering involving twisting modes and transverse acoustic modes, e.g., a longitudinal acoustic phonon (LA) decaying into a twisting acoustic phonon and a transverse acoustic phonon from branch 1 (TA1)^55^. This channel is very efficient for LA modes below 11 THz. However, above 11 THz, the LA mode has more than twice the energy of the other two modes, such that this channel becomes energetically forbidden for three-phonon scattering processes. This leads to the extreme increase in phonon lifetimes around 11–16 THz. As the corresponding phonons are also characterised by rather high group velocities and appreciable mode heat capacities, their mode contributions to the thermal conductivity dominate. This is illustrated in Fig. 5a by the blue bars for the integral mode contributions in the 11–16 THz windows and for the phonons along the Γ−Z-path in Fig. 5b. The cumulative thermal conductivity plotted in Fig. S4 reveals that the modes between 11 and 16 THz contribute 79% of the thermal conductivity for the 300 K unit cell. A similar behaviour is observed for the DFT-optimised cell, as shown in ref. ^10^.

In this context, it is critical to realise that the above-discussed energy-derived selection rules exclusively apply to three-phonon scattering processes. That means that phonons at 11–16 THz can still scatter with phonons below 5.5 THz, when one considers four- or higher-order phonon scatterings. Such processes are, however, neglected in the ALD-BTE approach implemented here. This runs the risk of massively overestimating contributions from 11 to 16 THz phonons. This suspicion is confirmed by comparing the mode-resolved data for the ALD-BTE and for the MD-BTE calculations in Fig. 5. When including the full anharmonicity (i.e., in the MD-BTE case), significantly reduced phonon lifetimes and mode contributions to the thermal conductivity are obtained in the range between 11 and 16 THz. Such reductions in thermal conductivity by including higher-order scattering have also been observed for other efficient thermal conductors, such as silicon and diamond^38^. Nevertheless, even in the MD-BTE calculations, the contributions at 11–16 THz are far from negligible, which makes sense, as the absence of three-phonon scattering processes due to selection rules still increases phonon lifetimes, just not to the extent predicted by the ALD-BTE calculations.

A direct consequence of high-frequency phonons still contributing significantly also in the MD-BTE simulations is that for PE, there are comparably large differences in the thermal conductivities when using different statistics for describing the mode heat capacities (see Table 5). This is because for a given temperature, the differences in mode heat capacities increase with increasing frequency (see Fig. 2). This explains why switching from C_BE_ to C_EQ_ in PT results only in a moderate increase in the thermal conductivity of 19% (see Table 3), while the increase is rather significant in PE, amounting to 46% (see Table 5).

**Table 5:** Thermal conductivity of crystalline PE along the chain direction calculated with different methods

Method	Unit cell	Thermal conductivity/Wm^−1 ^K^−1^
MD-BTE(${C}_{{BE}}$)	DFT-relaxed	95
MD-BTE(${C}_{{EQ}}$)	DFT-relaxed	135
MD-BTE(${C}_{{BE}}$)	300 K	78
MD-BTE(${C}_{{EQ}}$)	300 K	114
MD-BTE(${C}_{{EQ}},\frac{{\kappa }_{{full}\,{BTE}}}{{\kappa }_{{RTA}}}$)	300 K	146
NEMD	300 K	linear fit: 115 ± 20 2nd order fit: 146 ± 22
AEMD	300 K	127 ± 3

Uncertainties in NEMD and AEMD are reported as standard deviations (68% confidence) derived from the finite-size extrapolation (see Sections S8 and S10 for details). Heat capacities are treated according to the Bose–Einstein (BE) distribution, C_BE_, or following equipartition statistics (EQ), C_EQ_. The ratio between the full ALD-BTE and the ALD-BTE in RTA is 309 Wm^−1 ^K^−1^ divided by 242 Wm^−1 ^K^−1^ (see Table 4). This ratio is used to scale the thermal conductivity of the MD-BTE simulation, yielding the result denoted as MD-BTE(${C}_{{EQ}},\frac{{\kappa }_{{fullBTE}}}{{\kappa }_{{RTA}}}$).

**Fig. 6:**
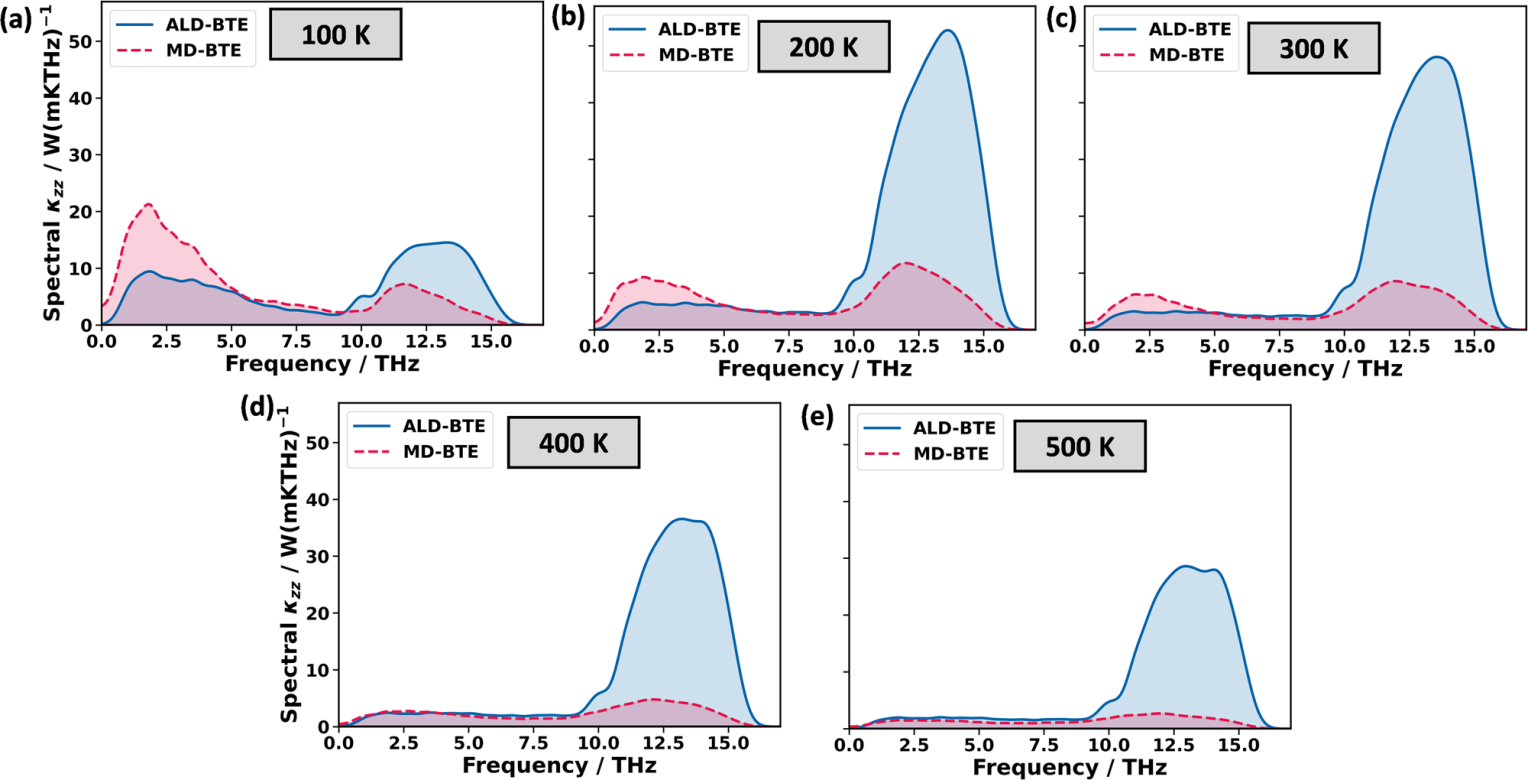
Temperature-dependent spectrally resolved contributions to the thermal conductivity. Spectrally resolved contributions to the thermal conductivities of ALD-BTE and MD-BTE simulations at temperatures of 100K (**a**), 200K (**b**), 300K (**c**), 400K (**d**), and 500K (**e**). In passing, we note that PE melts at around 400 K, but for the timescales on the order of ns that are employed in the MD simulations, this does not have a major impact on the simulations. The spectrally resolved contributions to the thermal conductivity are calculated as the derivative of the cumulative thermal conductivity. The cumulative thermal conductivity is smoothed by a 1-D Gaussian filter with 1 THz standard deviation for the Gaussian kernel; the spectrally resolved contributions to the thermal conductivity are smoothed with a 4 THz standard deviation.

A complication in assessing the role of higher-order scattering processes is that their impact in the MD-BTE calculations cannot be straightforwardly disentangled from effects that result from employing the equipartition theorem. In fact, we attribute the increased mode contributions between 0 and 4 THz, which is apparent in Fig. 5a, to the use of classical statistics for the determination of phonon lifetimes in the MD-BTE calculations. This assessment is based on the observation that the differences between low-frequency ALD-BTE and MD-BTE mode contributions diminish at higher temperatures, at which the classical equipartition statistics and the BE statistics converge (see Fig. 6). In contrast, at higher temperatures, the huge (relative) difference between ALD-BTE and MD-BTE calculations between 11 and 16 THz increases even further. This contradicts the idea that occupation statistics are responsible for the differences in phonon lifetimes between 11 and 16 THz, and strongly suggests that the differences are caused by higher-order scattering events.

Just as for PT, we also performed NEMD and AEMD simulations for PE. As described already for PT, to compare the MD-BTE to NEMD and AEMD, it is useful to consider the mode heat capacity calculated with equipartition statistics and to rescale the MD-BTE results with the ratio between the full ALD-BTE calculation and the ALD-BTE calculation in the RTA. This yields a thermal conductivity of 146 Wm^−1 ^K^−1^ for the rescaled MD-BTE case (see line labelled as MD-BTE(${C}_{{EQ}},\frac{{\kappa }_{{full\; BTE}}}{{\kappa }_{{RTA}}}$) in Table 5). Notably, this value compares very well with that of the finite-size extrapolated AEMD calculations, which amounts to 127 Wm^−1 ^K^−1^. It is also close to the outcome of the NEMD calculations, which, depending on the extrapolation scheme, yield 115 Wm^−1 ^K^−1^ for the linear fit and 146 Wm^−1 ^K^−1^ for the 2nd order polynomial fit (see discussion in Section S8). The rather pronounced difference between the two different extrapolation schemes can be attributed to the material’s very high thermal conductivity and the associated large phonon mean free paths (of up to 1.5 μm for the ALD-BTE calculation). As a consequence, even the largest considered cells of PE containing 1440 repeat units in chain direction and in total ca. 103,000 atoms (system sizes only possible due to the extreme numerical efficiency of MTPs) are still too small to allow fully reliable finite-size extrapolations. This is discussed in more detail in Section S8.

## Discussion

When describing thermal transport in complex materials like crystalline polymers, one has to overcome two challenges: (i) describing interatomic interactions in a numerically efficient way at a high level of accuracy, and (ii) choosing a suitable theoretical approach for modelling transport processes either in reciprocal space, i.e., based on phonon properties, or in real space, i.e., based on atom trajectories. The first challenge can nowadays be overcome using MLPs^10,16,17,23^, which we also apply here. In particular, we use moment tensor potentials^18^, whose parametrisations are system-specific and tailored to the particular use case^10^. This provides close to DFT-level accuracy combined with a speedup of many orders of magnitude compared to DFT. The speedup becomes particularly pronounced for large supercells, considering that DFT typically scales with the number of atoms to the power of three, while the computational effort associated with MTPs increases linearly.

When describing heat transport via phonons employing the BTE, the key challenge is to obtain phonon lifetimes, which we obtain here either from anharmonic lattice dynamics (ALD-BTE) or from the trajectories of molecular dynamics runs (MD-BTE). ALD-BTE is restricted to three-phonon scattering processes (since the direct computation of four-phonon scattering processes is prohibitively expensive for systems as complex as the ones studied here). An advantage of ALD-BTE is that it applies the correct quantum statistics for phonon occupations. Conversely, MD-BTE considers the full anharmonicity of the potential energy surface but describes phonon occupations only via the equipartition theorem. Despite these fundamental differences, we observe for PT an excellent agreement between ALD-BTE and MD-BTE not only in the overall thermal conductivity but also concerning the contributions of individual phonon modes. Importantly, for PT, the results of these two reciprocal space approaches also agree very well with those obtained from real-space descriptions of heat transport when applying either non-equilibrium molecular dynamics (NEMD) or approach-to-equilibrium molecular dynamics (AEMD) simulations. In this context, it is worth mentioning that the excellent agreement between the various, fundamentally different approaches is obtained even though NEMD and AEMD simulations rely again solely on classical statistics. This good agreement can be explained by the fact that the main heat carriers are phonons with low frequencies (below about 4 THz), and that the classical equipartition statistics and the BE statistics at 300 K converge towards each other at such low frequencies. Based on these arguments and the excellent agreement between ALD-BTE, MD-BTE, NEMD, and AEMD, we conclude that the respective approximations of these methods—namely, neglecting four-phonon scattering in ALD-BTE and employing classical statistics in MD-BTE, NEMD, and AEMD—have only a minimal impact on PT.

The situation is fundamentally different for PE, where, due to the simpler structure of the polymer chain and, thus, due to the reduced number of phonon bands, three-phonon scattering processes in certain frequency regions are diminished by energy-based selection rules. This results in particularly high phonon lifetimes for the states between 11 and 16 THz, such that they dominate thermal conduction in the ALD-BTE simulations. The large number of long-lived phonons with appreciable group velocities results in extremely high thermal conductivities of 309 Wm^−1 ^K^−1^ at 300 K. A comparison to the MD-BTE simulations, however, suggests that this is an artefact of neglecting higher-order scattering processes, which for PE is commonly done in the literature^55,56,64^. The inclusion of higher-order scattering massively reduces the phonon lifetimes at high frequencies and, thus, reduces the thermal conductivity by more than a factor of two. Nevertheless, the phonons between 11 and 16 THz are still comparably long-lived also in the MD-BTE simulations, as the typically dominant three-phonon scattering is still suppressed. Thus, even in the MD-BTE simulations, contributions from 11 to 16 THz phonons exceed those of the lower-frequency phonons. This is problematic because BE statistics and equipartition statistics differ significantly for high-frequency phonons. Indeed, for PE, this results in a sizable difference between MD-BTE simulations using the two statistics for describing mode heat capacities. This suggests that, in cases like PE, where also higher-frequency phonons contribute significantly to the thermal conductivity, the need for employing classical statistics can pose a sizable challenge at room temperature. Overall, by using an accurate, yet numerically extremely efficient description of inter-atomic interactions, we demonstrate that heat transport can be described consistently using either real space approaches based on particle trajectories (in MD approaches like NEMD or AEMD), or in reciprocal space approaches via phonons and their lifetimes (employing the BTE). The agreement between the complementary methods extends even to rather complex materials like crystalline polymers. However, this requires that the simulations accurately capture the physical intricacies of the materials. This can become particularly challenging for an ‘ill-behaved’ material like polyethylene, where high-frequency phonons (between 11 and 16 THz) contribute significantly to the thermal conductivity. Accurately describing this behaviour requires a careful treatment of higher-order phonon processes and, at the same time, challenges the use of classical statistics prevalent in MD approaches. Ironically, the challenges encountered in PE are a direct consequence of the relatively simple structure of the material, which makes three-phonon scattering less dominant than usual due to selection rules stemming from energy conservation.

## Methods

For the current manuscript, we mostly employed the same settings as in our previous publication, where we thoroughly benchmarked the accuracy of MTPs for describing crystalline polymers^10^. There, we demonstrated different ways to improve the accuracy of the MTPs, and we showed the (compared to DFT) very encouraging performance when calculating phonon-related properties. These include the phonon band structure, the thermal conductivity via the ALD-BTE approach, thermal expansion, and elastic constants.

### Density functional theory and active learning

VASP (Version 6.3.0^20^) was employed for two different tasks: It was used to perform ALD-BTE calculations of PE using the phono3py package^11^. Additionally, we used VASP to generate training data for PE and PT during active learning MD runs^21,22^. For the active learning runs, temperature ramps from 15 to 100 K and from 15 to 500 K were employed (see Section on ‘Moment tensor potentials’ for details). Simulations were performed in an *NpT* ensemble, and simulations from 15 to 100 K were additionally performed in an *NVT* ensemble with the unit cell fixed to the 300 K unit cells (as explained in more detail in the Section on ‘Moment tensor potentials’). In every case, a time step of 0.5 fs was used, and 150 MD steps were performed per Kelvin temperature increase. This yielded 72750 MD steps for the simulation from 15 to 500 K. In these simulations, a Langevin thermostat with a friction coefficient of 10 ps^−1^ was used, and the fictitious mass of the lattice degrees of freedom was set to 1000 amu. For the training data, the energy cut-off was set to 900 eV (PE) and 700 eV (PT). Convergence tests for these cut-offs and for the k-point sampling can be found in the Supplementary Materials of ref. ^10^. For the ALD-BTE calculation of PE with DFT, due to the high computational cost, we initially employed a 700 eV cut-off. For the sake of comparison, we also performed an ALD-BTE calculation for one of the PE unit cells (the one optimised at 300 K) with a cut-off of 900 eV. This yielded a somewhat higher thermal conductivity of 309 Wm^−1 ^K^−1^ (instead of 288 Wm^−1 ^K^−1^ for the 700 eV cut-off). This has no impact on the above discussion and is in the range of ‘benign’ variations of clearly below 10%, which we typically observe for crystalline polymers when changing details of the computational settings (e.g., when switching between MTPs in MD-based simulations as described in Sections S16 and S17). Thus, the calculation with the DFT-relaxed cell was performed with a 700 eV energy cut-off and the one for the 300 K cell with a 900 eV cut-off. For the DFT simulations, we employed the projector augmented wave method^19,65^ and the PBE functional^66^ complemented by Grimme’s DFT-D3 correction^67,68^ with Becke–Johnson damping^69^. This functional and vdW-correction have been shown to accurately describe phonons of organic semiconductors^62,70–72^. The Gaussian smearing of the occupancies of electronic states was set to 0.05 eV.

### Moment tensor potentials

We used moment tensor potentials (MTPs) as extremely efficient system-specifically parametrised MLPs. For a detailed description of their nature, we refer to the literature^18,73^. In a previous publication, we benchmarked MTPs and devised a protocol for their efficient use in combination with VASP-based active learning for metal-organic frameworks^23^. This strategy was then extended to polymers and to the specific needs of calculating anharmonic force constants^10^. Thus, here only a short description of the procedure is provided. DFT-calculated training data were generated in VASP during an active learning MD run, as described in the previous section. MTPs were trained using the MLIP code^18^ (version 2). Training of the MTP stopped when the normalised cost function (built from weighted deviations in forces, energies, and stresses between MTP results and reference data) dropped by less than a factor of 10^−3^ over the previous 50 iterations. This is the default setting in MLIP. For the calculation of the cost function, we used the default weights of 1 (eV)^−1^, 0.01 Å(eV)^−1^, and 0.001 (GPa)^−1^ for energies, forces, and stresses, respectively. The radial basis set size of the MTPs was set to 10. Carbon atoms of PT were split into two atom types, namely those that are bonded to sulphur and those that are not. This drastically improved the accuracy of the MTPs^10^.

In our previous work^10^, we showed that it is highly advantageous to consider lattice dynamics and molecular dynamics as two distinct use cases and to choose the hyperparameters accordingly, such that optimal performance can be achieved in each of the two simulation approaches. For the MTPs used in MD simulations, we, thus, considered the full set of training data obtained in active learning runs from 15 to 500 K in the *NpT* ensemble (see Section on ‘Density functional theory and active learning’). The resulting MTPs are referred to as MTP^MD^. For the MTPs used in the BTE simulations, we only considered the subset of training structures generated between 15 and 100 K, which improves the accuracy of MTPs in describing the phonon band structure by around a factor of two as compared to the case when all 15–500 K data are used. This improvement occurs because when using only low-temperature training data, the atomic displacements are rather small and are similar to the displacement encountered in phonon band structure or ALD-BTE calculations^10^. The MTP trained only on the low-temperature data is referred to as MTP^phonon^. Another factor to consider is the level of the MTP, which determines the size of the basis set and, consequently, the number of parameters^18,73^. MTPs with a higher level are more accurate, but notably slower. Since the calculation of third-order force constants for the BTE calculations is very efficient with MTPs, we could use a high level of 26 for MTP^phonon^. For MD calculations, where computational efficiency is more important, we had to use a lower level. Thus, the MTP^MD^s are level 22 potentials. A further differentiation is that the MTP^phonon^s are either applied to the DFT-relaxed unit cell or the 300 K unit cell. When performing MD simulations in an *NpT* ensemble at 15–100 K, the unit cell is similar to the DFT-relaxed unit cell, and thus the resulting MTPs are well-suited for describing the DFT-relaxed unit cell. However, the 300 K unit cell is substantially different from the DFT-relaxed unit cell (especially for PE). Therefore, we created a separate training set sampled at 15–100 K in an *NVT* ensemble with the unit cell fixed to the 300 K unit cell for parametrising MTP^phonon^s for that cell.

The performance of the five MTP^phonon^s, which were independently parameterised for each set of training data and unit cells with different random initialisations, was then ranked via the root mean square deviation (RMSD) between the MTP-calculated and the DFT-calculated phonon frequencies in the entire Brillouin zone up to 12.5 THz (where the exact value of that frequency cut-off has virtually no impact on the ranking, as shown in ref. ^10^). The performance of MTP^MD^s was evaluated on structures sampled at 300 K in a VASP active learning run, which was perfromed independently of the generation of the training data. Here, the MTP^MD^ with the smallest RMSD for the calculated forces on atoms is chosen as the ‘best’ one.

The NEMD and AEMD simulations (and here especially the finite-size extrapolation) generated gigantic computational efforts caused by the very large thermal conductivity along the chain direction. This required huge supercells in chain direction comprising up to 103680 atoms to at least approach the mean free path of the phonons, and the need to solve the equations of motion 4.3 million times. At the point in time the simulations were performed, the optimised procedure for parametrising MTPs outlined in ref. ^10^ was not yet fully established, and we used a somewhat different (first-generation) parametrisation strategy for those MTPs as described in Section S11. To make sure that this does not result in serious deviations in the results, we repeated the AEMD simulation with consistent MTP^MD^s for individual, relatively small unit cells. For the considered unit cell lengths (three per material), deviations in the same range as those found between AEMD thermal conductivities calculated with independently parametrised MTP^MD^s were obtained (see Sections S11 and S16). This suggests that the first-generation MTPs are acceptable for the analysis provided in the current paper.

### Molecular dynamics simulations

All MD calculations were performed in LAMMPS^74^ (Large-scale Atomic/Molecular Massively Parallel Simulator) using the version from the 2nd of July 2021, whereby we used the LAMMPS-MLIP interface (version 2)^18^. For the NEMD, AEMD, MD-BTE, and ALD-BTE calculations, we chose a unit cell that corresponds to the average lattice parameters at 300 K. To find this thermally expanded unit cell, we performed MD runs with the first-generation MTPs described in the previous section. These MTPs yield unit cells very well consistent with those obtained with MTP^MD^s, which are provided in ref. ^10^. In the MD runs, we used a 4 × 6 × 12 supercell for PE and a 4 × 6 × 4 supercell for PT with extents of approximately 30 Å in each direction. The time step was set to 0.5 fs. The converged simulation times were 500 ps for PT and 700 ps for PE. The first 50 ps (PT) and 100 ps (PE) were neglected in the averaging to account for equilibration effects. A Langevin thermostat with a 0.1 ps damping coefficient was used in these simulations.

### Non-equilibrium molecular dynamics

For the non-equilibrium molecular dynamics (NEMD) simulations^12^, the unit cell was fixed to the one that was obtained from the thermal expansion calculation at 300 K (see above). We used the Langevin thermostat for the equilibration before the actual NEMD calculation. For the NEMD calculation, the Müller-Plathe method in an *NVE* ensemble was applied^12^. The temperature gradient was evaluated as described by Li et al. by taking the difference between the temperatures of the hot and cold regions^45^. There exists an alternative definition of the temperature gradient, which is discussed in Section S7. We note that only when defining the temperature gradient as suggested by Li et al.^45^, results consistent with AEMD and MD-BTE simulations were obtained. The finite-size extrapolation, as well as further details on the NEMD simulation, are provided in Sections S7 and S8.

### Approach-to-equilibrium molecular dynamics

In approach-to-equilibrium molecular dynamics (AEMD)^46,47^ simulations, one half of the simulation box is heated to a hot temperature ${T}_{1}$ and the other half to a colder temperature ${T}_{2}$. In our case, ${T}_{1}$ was 350 K, ${T}_{2}$ was 250 K, and, thus, we obtained the thermal conductivity around 300 K. Details on the calculation of the thermal conductivity from the time evolution of the temperature difference between the hot and cold regions, including convergence considerations, are provided in Sections S9, S1.4, and S1.5. Again, thermal conductivities are corrected for finite-size effects by calculating differently sized supercells as shown in Section S10.

### Boltzmann transport equation from anharmonic lattice dynamics (ALD-BTE)

ALD-BTE calculations were performed with the DFT-relaxed as well as with the 300 K unit cells. Phonon band structures were calculated with phonopy^11^ with a 2 × 3 × 6 and 3 × 2 × 4 supercell for PE and PT, respectively (see ref. ^10^ for convergence tests). ALD-BTE calculations were performed with the phonop3y package^11^, with the same settings as in our previous publication, which also provides convergence tests for PE^10^. The default displacement amplitude of 0.03 Å was used in phono3py, for which convergence tests can be found in Section S1.7 and in ref. ^10^. The supercells for calculating the third-order force constants had dimensions of 2 × 2 × 3 and 2 × 2 × 2 for PE and PT, respectively (see ref. ^10^, for convergence tests). Brillouin zone integration in the ALD-BTE calculation was performed with the tetrahedron method^75^. The meshes for sampling the Brillouin zone were generally chosen such that the thermal conductivity in the chain direction is converged to within 5% (for details see Section S1.6). Brillouin zones of PE and PT were sampled with a 4 × 6 × 160 mesh and a 4 × 6 × 48 mesh, respectively.

### Boltzmann transport equation with lifetimes from molecular dynamics (MD-BTE)

Simulations to obtain the phonon lifetimes from molecular dynamics (MD-BTE) were performed with the DynaPhoPy package version 1.17.15^40^. We improved the memory efficiency of the code, which allowed the computation of structures with a larger number of atoms in the primitive unit cell (see ‘Code availability’ Section). The required MD simulations were performed in LAMMPS with the MTP^MD^ force fields (see Section on ‘Moment tensor potentials’). The used supercells were 2 × 3 × 48 and 2 × 3 × 160 for PT and PE, respectively (see Section S1.2 for convergence tests). The MD runs at 300 K were performed for 1 ns (PE) and for 2 ns (PT) (following the convergence tests illustrated in Fig. S1) with a timestep of 0.5 fs. For other temperatures, somewhat larger simulation times were necessary, as detailed in Section S3. The velocities were written out for every 10th time step, which had only a negligible impact on the results compared to evaluating the velocities every time step. The resolution of the power spectrum was set to 0.004 THz for PE and 0.002 THz for PT (see Section S1.1 for a convergence test). The lifetimes were computed for all symmetry-inequivalent grid points to be consistent with the phono3py implementation, which was then employed to obtain the thermal conductivities.

## Supplementary information

SI-paper_heat_transport_rev2.

## Data Availability

Datasets generated and/or analysed during the current study are available in the TU Graz Repository; 10.3217/jw2ym-g7r83.

## Code availability

The modified version of Dynaphopy, that was used in this study, is available at www.github.com/sandrowieser/DynaPhoPy/tree/orig-ez-paper. VASP can be acquired from the VASP Software GmbH (see www.vasp.at); LAMMPS is available at www.lammps.org; MLIP is available at www.mlip.skol-tech.ru/download; the lammps-mlip interface (version 2) is available at www.gitlab.com/ashapeev/interface-lammps-mlip-2; Phonopy is available at www.phonopy.github.io/phonopy; Phono3py is available at www.phonopy.github.io/phono3py.
