# Supplementary material for: Analysing heat transport in crystalline polymers in real and reciprocal space
Source: NPJ Comput Mater. 2026 Feb 18;12(1):129. doi: 10.1038/s41524-026-01988-0 (PMC13031126; doi:10.1038/s41524-026-01988-0)
Supplement: Supplementary file 1 — SI-paper_heat_transport_rev2. [file 41524_2026_1988_MOESM1_ESM.pdf]

SUPPLEMENTARY INFORMATION

Analysing Heat Transport in Crystalline Polymers in Real and Reciprocal Space

Lukas Reicht[1], Lukas Legenstein[1], Sandro Wieser[1,2], and Egbert Zojer[1,*]

[1] Institute of Solid State Physics, Graz University of Technology, NAWI Graz, Graz, Austria
[2] Institute of Materials Chemistry, TU Wien, Vienna, Austria
* Correspondence: egbert.zojer@tugraz.at

Table of Contents

S1 Convergence tests 3
S1.1 Simulation time and resolution in MD-BTE calculations 3
S1.2 Supercell size in MD-BTE calculations 6
S1.3 Level of the MTP 7
S1.4 Simulation time in AEMD 8
S1.5 Number of exponentials in fit of AEMD data 9
S1.6 Q-mesh in ALD-BTE calculations 9
S1.7 Displacement amplitude in ALD-BTE calculations 11
S2 Comparison with existing literature 12
S2.1 Comparison of polythiophene's thermal conductivity to the simulation results of Cheng et al. 12
S2.2 Comparison of polyethylene's thermal conductivity with results of Wang et al. 14
S3 Phonon tunneling contribution to the thermal conductivity 15
S4 Comparing ALD-BTE and AEMD results for the thermal conductivity in a vdW-bonded direction 17
S5 Effect of phonon renormalization 17
S6 Ioffe-Regel limit 20
S7 Further details on the NEMD calculations 20
S8 NEMD finite-size extrapolation 23
S9 Further details on the AEMD simulation 25
S10 AEMD finite-size extrapolation 26
S11 MTPs for NEMD and AEMD simulations 27
S12 DFT-relaxed and 300 K unit cells 28
S13 Complication with the Dynaphopy fit for PT for one of the considered q-points 29
S14 Frequency-resolved thermal conductivity with heat capacity according to equipartition and Bose-Einstein statistics 30

S15 Lifetimes of PT calculated with MD-BTE 31
S16 Statistical noise in AEMD simulations and uncertainty of MTPs in AEMD 32
S17 Uncertainty of the MTPs in MD-BTE simulations of PE 34
S18 Thermal conductivities of additional MTPs with ALD-BTE 34
S19 Outlier MTP for the DFT-relaxed unit cell of PE 36
S20 References 38

S1 Convergence tests

S1.1 Simulation time and resolution in MD-BTE calculations

The Boltzmann transport equation calculations with lifetimes from molecular dynamics (MD-BTE) are tested regarding the convergence of the simulation time in the MD simulations used for determining phonon lifetimes. The results are shown in Figure S1. As a first step, we here converge the thermal conductivity value at 300 K. The convergence of the mode conductivities of individual phonon bands and for different temperatures is given further below. The calculations are performed with a 2 × 3 × 24 supercell for PT and with a 2 × 3 × 80 supercell for PE. Due to the particularly large supercell for PE, we performed the convergence test for this material with a level 18 MTP, which gives similar results to the level 22 MTP used in the main text (see Figure S7). MD-BTE calculations for PT are converged to within 5% after 1.5 ns when using a resolution of 0.004 THz for the power spectrum (see Figure S18 for an example of the power spectrum), and for PE, an equivalent convergence is reached after 1 ns. Alternatively, at a resolution of 0.002 THz, a simulation time of 1.5 ns is needed to converge the results to within 5%. Regarding the resolution, the difference between 0.002 THz and 0.004 THz is so small that 0.002 THz can be considered well converged.

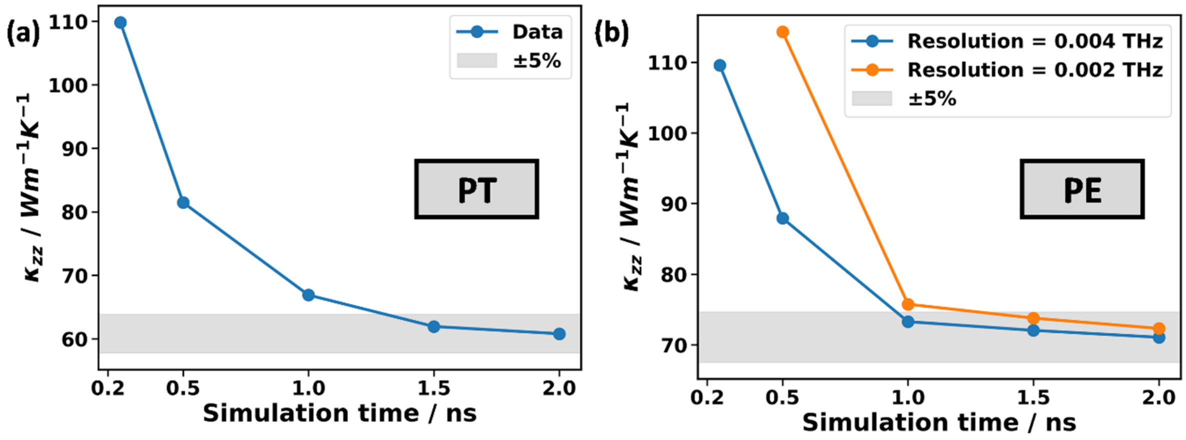

Figure S1: Thermal conductivity along the chain calculated with MD-BTE with different simulation times for (a) PT and (b) PE. For PT, the resolution of the power spectrum is set to 0.004 THz. For PE, resolutions of 0.002 THz and 0.004 THz are used. For the calculations with the lowest resolution and largest simulation time (i.e., the most converged calculation), a shaded grey region is drawn at ± 5%.

While the thermal conductivity value at 300 K is sufficiently converged at these simulation times, individual phonon lifetimes, which do not contribute significantly to the thermal conductivity, take a bit longer to converge to a satisfactory degree. To be specific, for PT, the mode thermal conductivities are shown for MD-BTE calculations with simulation times of 1 ns, 1.5 ns, and 2 ns superimposed on the phonon band structures in Figure S2. The 1 ns calculation was performed with a resolution of 0.004 THz, while the 1.5 ns and 2 ns calculations were performed with a 0.002 THz resolution. A 2 × 3 × 48 supercell is used. Evidently, there are qualitative differences for the longitudinal acoustic (LA) mode close to the Γ-point between the 1 ns and the 1.5 ns calculations. It is sensible that differences occur for this mode, since this is the mode with the largest mode thermal conductivity. The 1.5 ns and 2 ns

calculations are qualitatively the same; therefore, we regard 1.5 ns as sufficient for achieving convergence. In passing, we note that the contribution of the "problematic" data points to the thermal conductivity is very small, since the volume in reciprocal space is so small close to the Γ-point (as argued in the main paper). To confirm this, the cumulative thermal conductivity is shown in Figure S3.

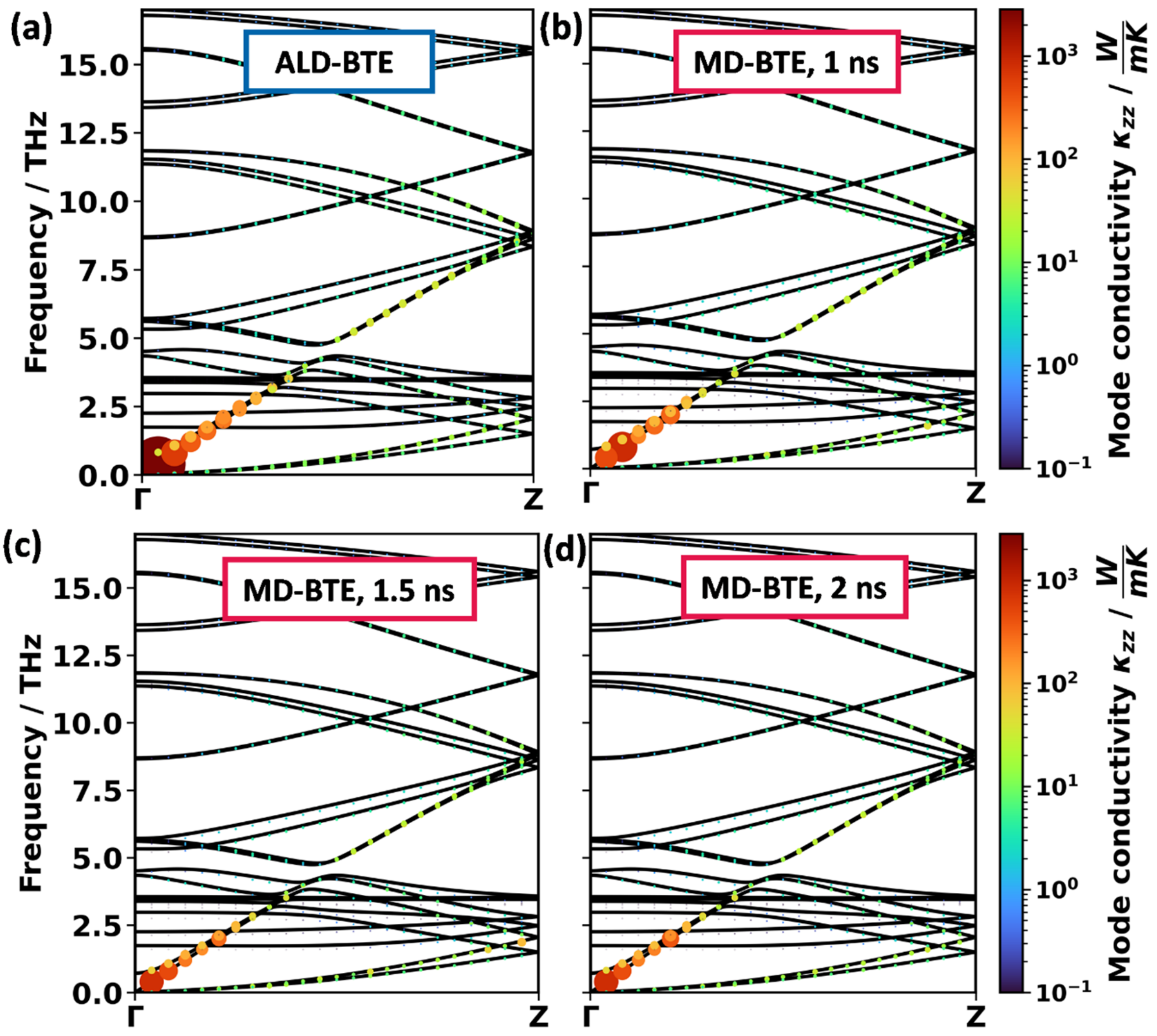

Figure S2: Phonon band structure of PT, whereby the areas of the data points are linearly enlarged depending on their mode thermal conductivity and coloured on a logarithmic scale. In panel (a), the ALD-BTE calculation is shown. The MD-BTE calculation is performed for different simulation times in the MD runs to determine phonon lifetimes in panels (b)-(d).

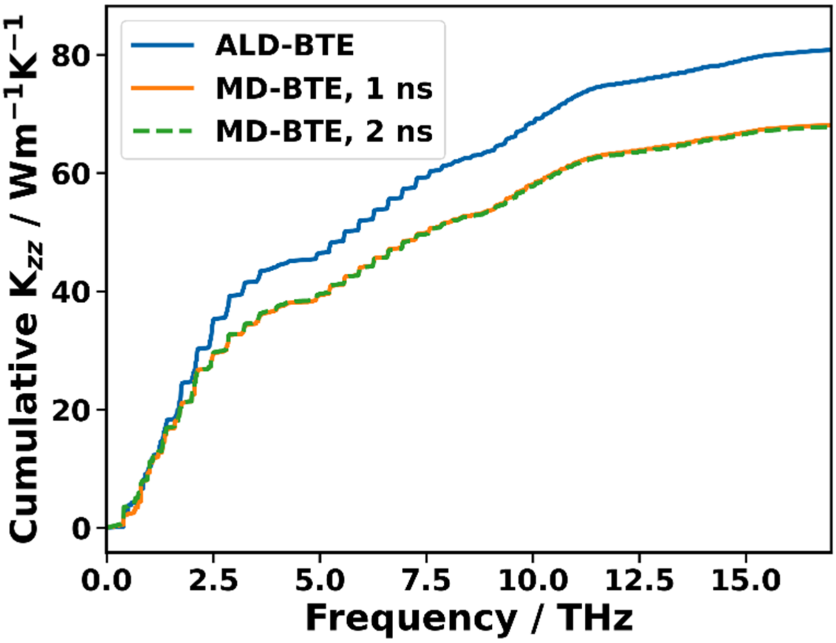

*Figure S3: Cumulative thermal conductivity of PT along the chain direction. For the MD-BTE calculations, simulation times of 1 ns and 2 ns are used, which has only a negligible influence on the cumulative thermal conductivity. The "steps" are due to the finite **q**-mesh sampling of the first Brillouin zone.*

While the MD-BTE simulation of PE at 300 K is very well converged with a simulation time of 1 ns and a resolution of 0.004 THz, this does not guarantee convergence at other temperatures. Thus, we checked the time convergence for the MD-BTE calculation of PE separately for each temperature. For example, Figure S4 shows a convergence test for the MD-BTE calculation at 100 K. As shown in the main paper, the thermal conductivity obtained with MD-BTE is larger at 100 K. This is caused by larger phonon lifetimes, which require longer simulation times and sharper resolutions of the power spectrum. Thus, for calculation at 100 K, 8 ns are necessary when using a resolution of 0.001 THz. At 200 K, already a simulation time of 1 ns and a resolution of 0.004 THz give reasonably well converged results, as shown in Figure S5. Still, since the calculation with the longer time was already performed, we used it in the main paper. Therefore, for the results in the main paper, the used simulation times were 8 ns at 100 K, 4 ns at 200 K, and 2 ns at 300 K, 400 K, and 500 K. The resolution was set to 0.001 THz at 100 K, and 0.004 THz at the other temperatures.

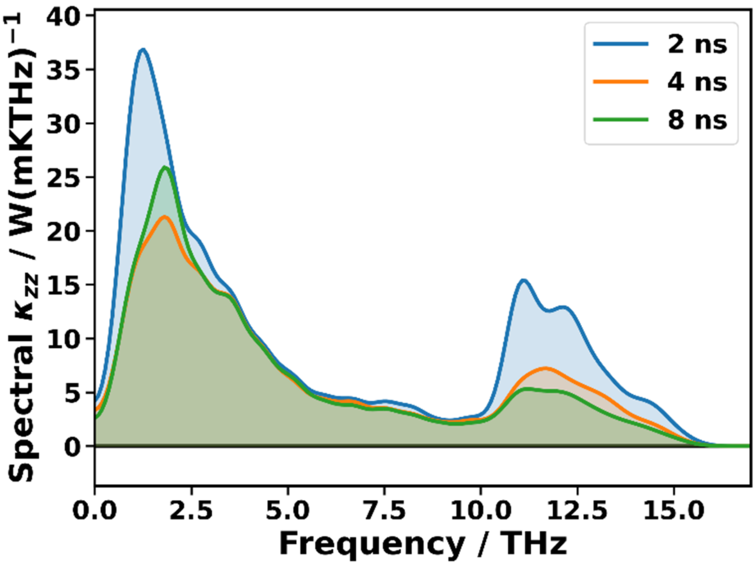

Figure S4: Spectrally resolved contributions to thermal conductivity of PE at 100 K calculated with MD-BTE for simulation times of 2 ns, 4 ns, and 8 ns in the MD runs to calculate phonon lifetimes. The resolution of the power spectrum is 0.001 THz.

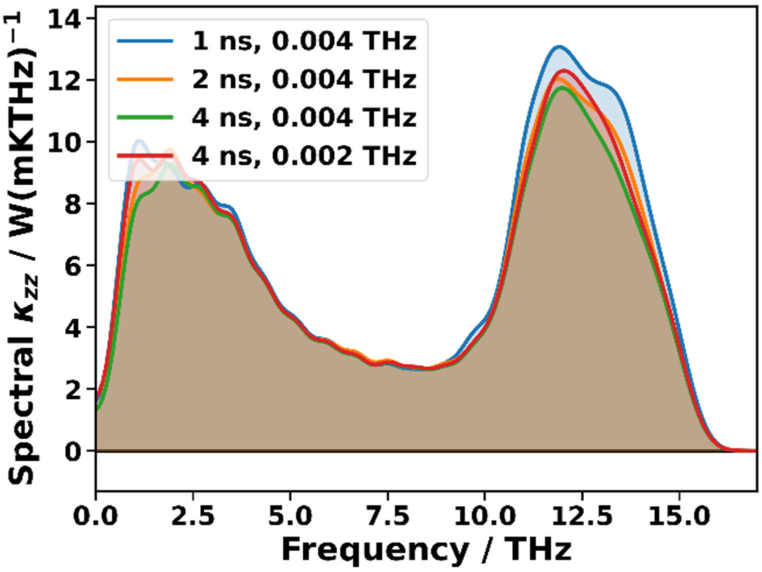

Figure S5: Spectrally resolved contributions to thermal conductivity of PE at 200 K calculated with MD-BTE. The simulation times are 1 ns, 2 ns, and 4 ns with resolutions of 0.004 THz and 0.002 THz as indicated in the figure legend.

S1.2 Supercell size in MD-BTE calculations

As a convergence test of the supercell sizes in MD-BTE, the MD-based calculations of phonon lifetimes were performed with different supercells. The resulting cumulative thermal conductivities are shown in Figure S6. For all four shown calculations, the simulation time in the MD runs was set to 1 ns, and the resolution of the power spectrum was set to 0.004 THz. As in the previous section, the calculations

of PE are performed with the level 18 MTP to save computational resources. The converged **q**-meshes from the ALD-BTE calculation (see Section S1.6) gave us an initial estimate for the supercell size, which we then tested by doubling it. For PT, the thermal conductivities are 66.9 $Wm^{-1}K^{-1}$ and 70.5 $Wm^{-1}K^{-1}$ with the 2 × 3 × 24 and 2 × 3 × 48 supercells, respectively. This amounts to a 5% difference. Considering this small difference and that the shapes of the cumulative thermal conductivities are similar, already the 2 × 3 × 24 supercell can be considered converged for calculating the thermal conductivity in the chain direction. Since the calculation with the larger 2 × 3 × 48 supercell had already been performed for the present test, this even better converged supercell was used in the discussion in the main paper. Since the unit cell of PT is three times longer than that of PE, more cell repetitions are required for PE to reach the same supercell length. Correspondingly, supercells of 2 × 3 × 80 and 2 × 3 × 160 were used, which have a similar length as the supercells of PT. For PE, the thermal conductivities are 73.3 $Wm^{-1}K^{-1}$ and 77.9 $Wm^{-1}K^{-1}$ with supercells of 2 × 3 × 80 and 2 × 3 × 160, respectively. Again, the cumulative thermal conductivities have a similar shape with both supercell lengths. The largest deviations are for phonons around 14 THz to 15 THz. Since the slopes of the cumulative thermal conductivities are similar and the thermal conductivities differ by only 6%, we again regard both supercell sizes as converged in this direction. Moreover, to test the supercell size in the directions perpendicular to the polymer chain, calculations with a supercell size of 2 × 3 × 80 and 4 × 6 × 80 were performed for PE. These show negligible deviations in the phonon lifetimes, spectral thermal conductivity and the thermal conductivity along the chain direction. Thus, we regard the 2 and 3 unit cell repetitions in the **x**- and **y**-directions as converged for calculations of the thermal conductivity along the chain direction.

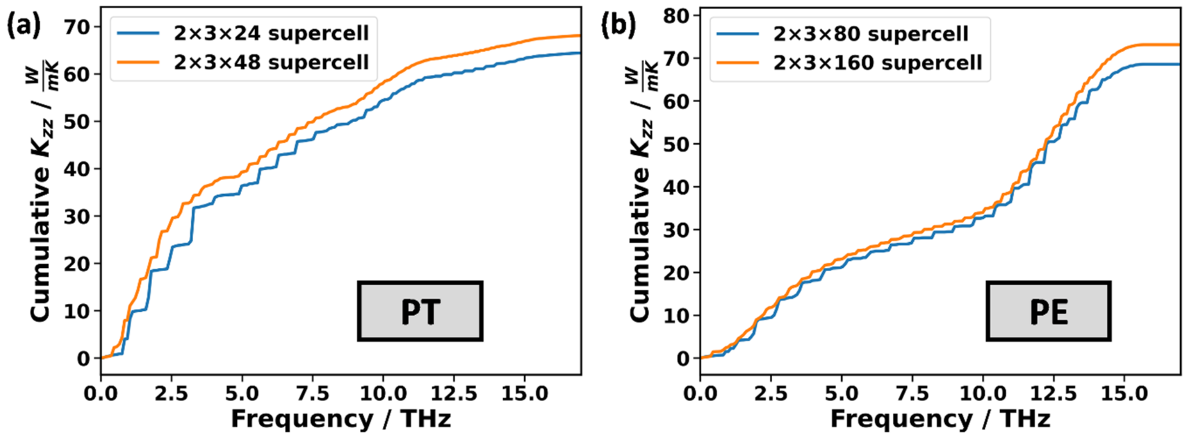

Figure S6: Cumulative thermal conductivity for different supercell sizes of (a) PT and (b) PE.

S1.3 Level of the MTP

In our previous publication[1] and in the Methods section of the main paper, we suggested a protocol for parametrizing an MTP meant for molecular dynamics simulations and referred to it as MTP^{MD}. It is trained on data sampled at 15 K to 500 K and has a level of 22. This MTP^{MD} is used for the MD-BTE calculation. To test the influence of the level, we parametrised five MTPs for PE with level 18 on the same training data that were used for the level 22 MTP^{MD}. The cumulative thermal conductivities calculated with the respective "best" MTPs are shown in Figure S7. Qualitatively, they agree very well.

Also quantitatively, the agreement is highly satisfactory: The corresponding thermal conductivities are 79 $Wm^{-1}K^{-1}$ and 84 $Wm^{-1}K^{-1}$ with the level 18 and level 22 MTPs, respectively. This difference is similar to the difference between the five individual MTP^{MD}s (see Section S18). In light of this very good agreement, we conclude that already the 18 MTP would be sufficiently accurate. In view of the results by Wu et al., who recently suggested that inaccuracies of machine learned potentials can lead to an underestimation of the thermal conductivity,[2] we picked the level 22 MTP for all calculations in the main manuscript. In this way, we are confident that the accuracy of the used MTP is so high that there is no need for applying the correction also suggested by Wu et al.[2].

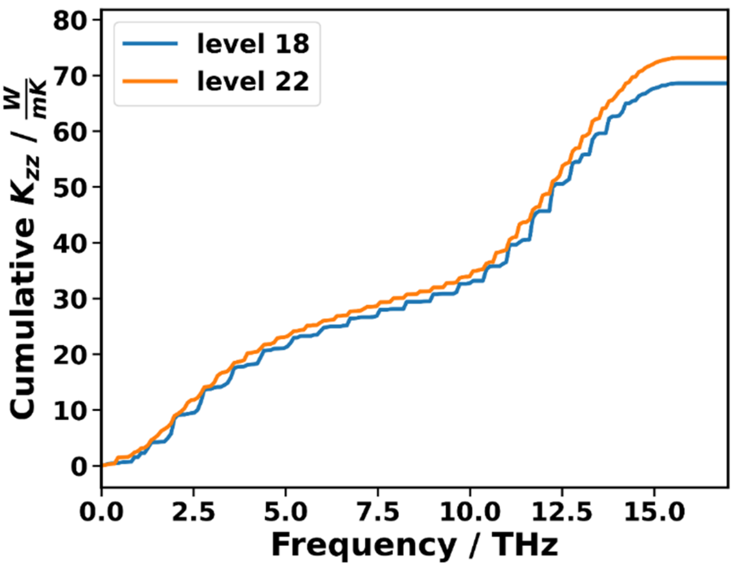

Figure S7: Cumulative thermal conductivity of PE calculated with MD-BTE using MTPs with level 18 and level 22. The supercell size is 2 × 3 × 160 and the simulation time when determining phonon lifetimes is 1 ns.

S1.4 Simulation time in AEMD

In AEMD simulations, the temperature difference decays as shown in Figure S8a for an example calculation on PT. The thermal conductivity is evaluated for different simulation times, and its convergence is plotted in Figure S8b. The thermal conductivity peaks at around 75 ps at a value differing by less than 5% from the value at 200 ps. Therefore, the thermal conductivity for times beyond 75 ps can be regarded as converged for this calculation, yet the even more converged 200 ps were used for the data reported in the main manuscript. For the calculation in Figure S8b, the unit cell was 256 repetitions long along the chain direction. When performing calculations with larger unit cells, the simulation time was scaled linearly with the length of the unit cell. This resulted in calculations that show a similar degree of convergence. Accordingly, the used calculation times are 100 ps, 150 ps, 200 ps, 300 ps, and 400 ps for unit cell repetition of 128, 192, 256, 384, and 512 times. The same analysis as just described for PT was also performed for PE. For PE, the simulation times are 60 ps, 100 ps, 150 ps, 200 ps, and 300 ps for unit cell repetition of 432, 720, 1080, 1440, and 2160 times. As mentioned above, more unit cell repetitions are needed for PE, since its unit cell is three times shorter than that of PT.

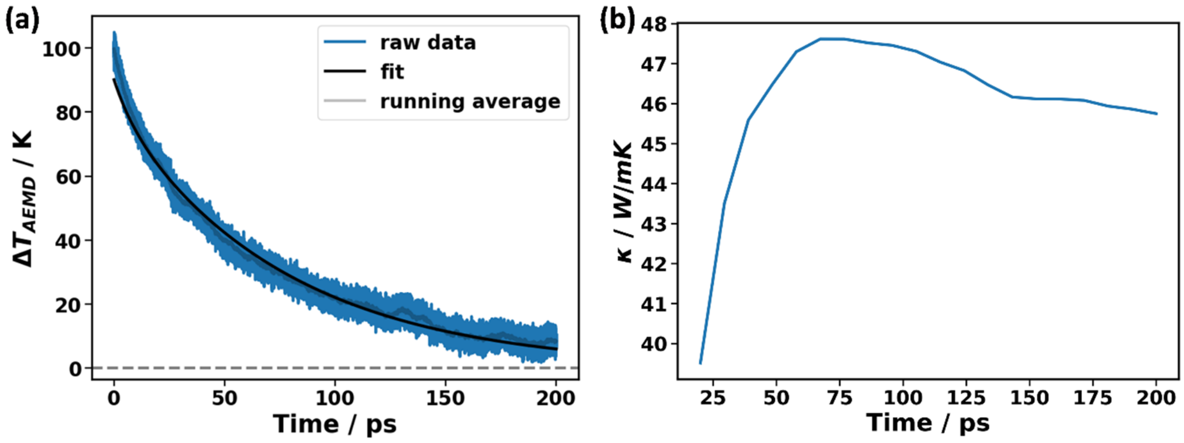

Figure S8: Panel (a): Temperature difference between hot and cold regions, ΔT_{AEMD}, decaying with time in an AEMD simulation of PT. The raw data is shown in blue and a running average in light grey. The fit that is used to evaluate the thermal conductivity is shown as a black line. Panel (b): Simulation time convergence of the thermal conductivity in an AEMD simulation of PT.

S1.5 Number of exponentials in fit of AEMD data

The temperature difference in AEMD simulations is fitted by a sum of exponentials, as detailed in Section S9. The thermal conductivity needs to be converged with respect to the number of exponentials, denoted as $\mathrm{N_{exp}}$. As shown in Figure S9, three exponentials are enough to reach a very convincing convergence. Therefore, three exponentials were used throughout the manuscript.

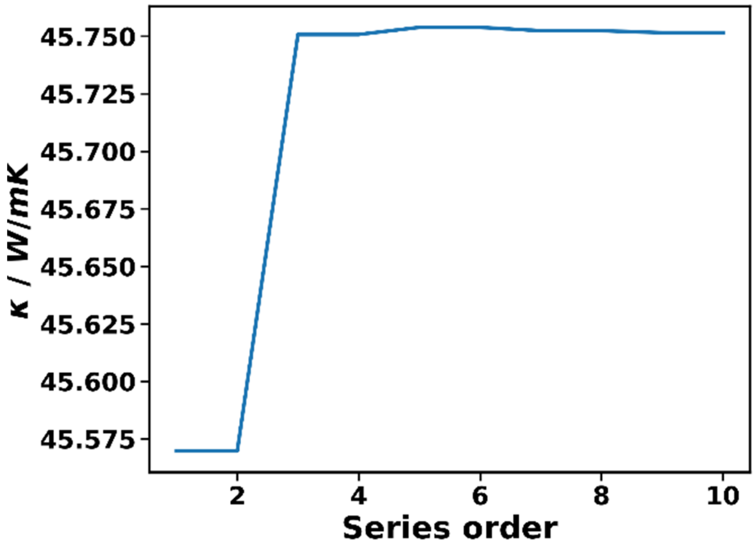

Figure S9: Convergence of the thermal conductivity with respect to the number of exponentials N_{exp} in the fit function.

S1.6 Q-mesh in ALD-BTE calculations

In ALD-BTE calculations, the Brillouin zone is sampled with a **q**-mesh, which has to be checked for convergence. For PT, a convergence test for the **q**-points along the chain direction is shown in Figure S10a. The directions perpendicular to the chain were investigated in a separate convergence test. The thermal conductivity along the chain direction κ_{zz} (with the full BTE and in RTA) is converged to within 5% for a 4 × 6 × 26 **q**-mesh. We note that this calculation was performed with an MTP that was parametrised in a slightly different way than the MTPs of the main manuscript. Namely, this MTP has a level of 28 and was trained on 169 training structures that were sampled at 15 K to 300 K. Since this MTP yields a similar thermal conductivity as the MTPs used in the main paper, we regard the results obtained for the **q**-mesh convergence test to be transferable also to the other MTPs. Therefore, the **q**-mesh convergence test was not repeated for the MTPs of the main paper.

For PE with the DFT-relaxed unit cell, a convergence test of the **q**-mesh can be found in the Supplementary Materials of Ref. [1] . The convergence behaviour for the 300 K unit cell is shown in Figure S10b. There, the **q**-points in **x**- and **y**-direction are held fixed at 4 and 6 points, while the number of **q**-points along the chain direction is varied. The numerical values corresponding to the data in Figure S10b are listed in Table S1. For the RTA, the convergence is rather benign, and already the thermal conductivity with a **q**-mesh of 4 × 6 × 80 differs by less than 5% from the values obtained for larger **q**-meshes. For the full BTE simulation, the situation is somewhat more involved with an outlier for 200 **q**-points. We opted to use the 4 × 6 × 160 **q**-mesh for all calculations of PE, since it is reasonably well converged with a roughly 5% difference in the thermal conductivity as compared to the 4 × 6 × 320 **q**-mesh, while remaining computationally manageable.

Alongside these values, we also list the total number of eigenvalues in the collision matrix and the number of negative eigenvalues in the collision matrix. A definition of the collision matrix can be found in equation (61) of Ref. [3]. From that equation, it is evident that the collision matrix is positive semidefinite, as long as all phonon frequencies are positive. We checked explicitly that, in fact, all phonon frequencies are positive. Therefore, we conclude that any negative eigenvalues of the collision matrix are a numerical artifact. The absolute values of these negative eigenvalues are comparable to or smaller than the arithmetic mean of the positive eigenvalues. Since there is only a very small number of negative eigenvalues compared to positive eigenvalues, we argue that these negative eigenvalues have no meaningful impact on the calculated thermal conductivities. We observe negative eigenvalues for roughly half of our simulations, including the simulations of PT. Their appearance seems random. For example, when parametrizing five MTPs for PT with different initialization, around half of them produce negative eigenvalues, while the corresponding five thermal conductivities are all in good agreement with each other. The appearance of negative eigenvalues does not at all correlate with whether the respective thermal conductivity values are reasonable. In particular, it does not explain the unreasonable thermal conductivities that are observed for the 300 K unit cell with PE when MTPs are used (see Section S18 for more details).

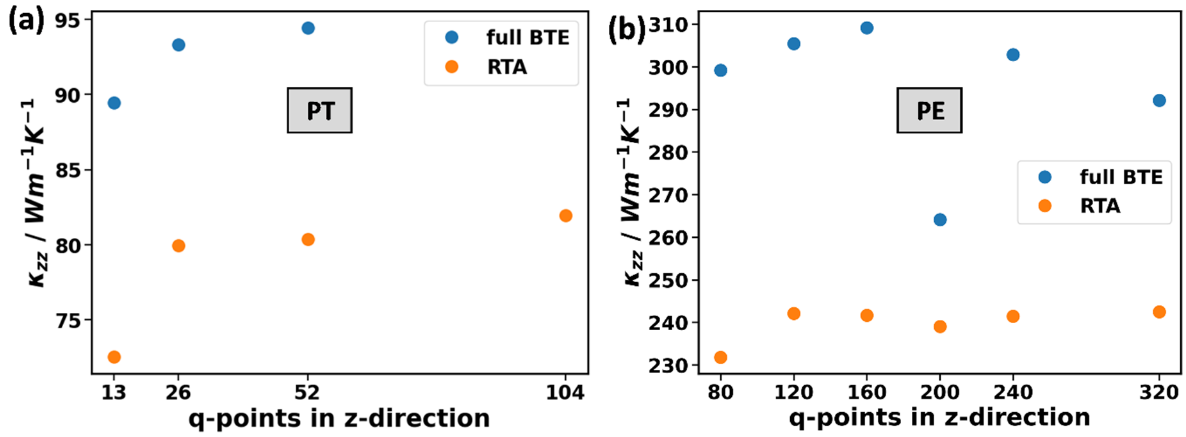

*Figure S10: **Q**-mesh convergence test along the chain direction for (a) PT and (b) PE. The calculation for PT was performed with the DFT-relaxed unit cell using an MTP, which was parametrised in a slightly different way than the MTPs of the main manuscript (see main text for details). For PE, the DFT calculation for the 300 K unit cell is shown.*

Table S1: Numerical values for the convergence test depicted in Figure S10b. Additionally, we list the total number of eigenvalues in the collision matrix and the number of negative eigenvalues in the collision matrix.

**q-mesh**	**4 × 6 × 80**	**4 × 6 × 120**	**4 × 6 × 160**	**4 × 6 × 200**	**4 × 6 × 240**	**4 × 6 × 320**
**κ_{zz} in RTA / $Wm^{-1}K^{-1}$**	231.8	242.0	241.6	239.0	241.4	242.4
**κ_{zz} full BTE / $Wm^{-1}K^{-1}$**	299.1	305.4	309.1	264.1	302.8	292.1
**Total number of eigenvalues**	53,136	79,056	104,976	130,896	156,816	208,656
**Number of negative eigenvalues**	3	3	4	4	4	5

S1.7 Displacement amplitude in ALD-BTE calculations

In phono3py calculations, atoms are by default displaced by 0.03 Å for calculating third-order force constants. This displacement amplitude can be controlled with the "amplitude" tag in phono3py. For too small displacement amplitudes, the numerical noise becomes too large, while for too large amplitudes, higher order anharmonicities play an increasing role and "distort" the calculated third order force constants. Thus, the goal is to find a region in which the magnitude of the displacement does not severely impact the results. To test the convergence of the displacement amplitude, values of 0.01 Å, 0.03 Å, 0.05 Å, 0.07 Å, 0.09 Å, and 0.11 Å were used. Examples of such convergence tests can be seen in Figure S11 for the DFT-relaxed cell of PE and the 300 K cell of PT. These tests were performed for the DFT-relaxed as well as the 300 K unit cells. They were also performed in the vdW-

bonded directions, respectively for the Peierls contribution, phonon tunnelling contribution, and total thermal conductivity (see Section S3 for details on the Peierls and phonon tunnelling contributions). Since showing all of these plots would be rather lengthy, we instead describe the outcome here in the text and show two representative plots in Figure S11. In all of these scenarios, the thermal conductivities for the displacement amplitudes of 0.03 Å and 0.05 Å agreed well. On the one hand, the displacement amplitude of 0.01 Å sometimes seemed too small for the vdW-bonded directions, and also in Figure S11b, as it deviates from the larger amplitudes. On the other hand, displacement distances beyond 0.05 Å tend to give thermal conductivities that deviate from the values at 0.03 Å and 0.05 Å. Thus, considering all the performed convergence tests, the displacement amplitude of 0.03 Å was found to give the best convergence. This displacement amplitude was also used in the calculations of the main manuscript. In addition to these comprehensive tests with the MTPs, the DFT calculation of PE for the DFT unit cell was performed with an amplitude of 0.03 Å and 0.05 Å, yielding very similar results (see ref. [1]).

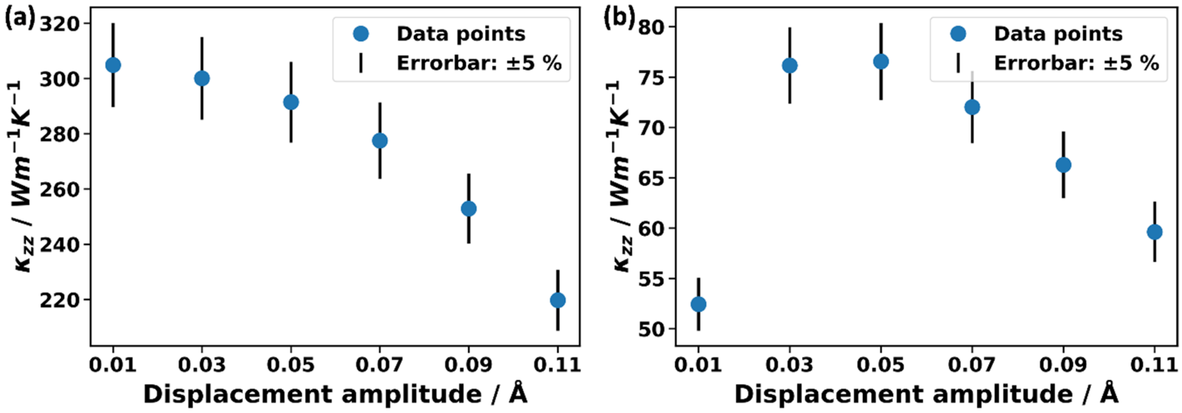

*Figure S11: Convergence of κ_{zz} with respect to the displacement amplitude in phono3py. Panel (a) depicts the MTP-calculated total thermal conductivity for the DFT-relaxed cell of PE. Panel (b) shows the calculation of PT with the 300 K cell. Since we applied a convergence criterion of 5% to the **q**-mesh, error bars of that magnitude are drawn to put the deviations into perspective.*

S2 Comparison with existing literature

S2.1 Comparison of polythiophene's thermal conductivity to the simulation results of Cheng et al.

Cheng et al. calculated the thermal conductivity of crystalline PT using the temperature-dependent effective potential (TDEP) method and obtained a thermal conductivity of 198 $Wm^{-1}K^{-1}$.[4] That result is rather different from the thermal conductivities reported in the main paper of the present study. To address this issue, we identified four possible reasons for this difference: These are the structure (herein referred to as "Cheng's structure" and "our structure"), the vdW-correction (Cheng used the original vdW-DF[5], while we used PBE+D3[6–8]), the different method (Cheng used the TDEP method, while we used ALD-BTE, MD-BTE, AEMD, and NEMD), and various convergence settings. Upon request,

the authors of the paper were so kind as to send us the structure that they used and details on their calculation. The structures differ insofar as in our structure, one of the PT chains is shifted along the chain axis, thereby reducing the symmetry. This intentional breaking of the symmetry is crucial so that the structure does not get stuck on a saddle point of the potential energy surface. Indeed, when we calculate phonons with Cheng's structure and the PBE+D3 functional, we find a negative Γ-mode that corresponds to a translation of one of the PT chains in the unit cell relative to the other. This is precisely the chain shift that entails the difference between our structure and Cheng's structure. A displacement along such a negative Γ-mode corresponds to a lowering in energy, thereby showing that Cheng's structure is not in an energy minimum (at least for the PBE+D3 functional). To investigate the matter further, we relax both structures with each of the DFT functionals. The resulting energy and volume can be found in Table S2 and Table S3. For both functionals, the structure with shifted polymer chains (i.e., "our structure") has a lower energy. These results suggest that "our structure" should be preferred, especially for calculations of phonons and BTE calculations. Notably, the energy barrier between our structure and Cheng's structure is very low, as can be inferred from the observation that, when performing molecular dynamics simulations in VASP, a shift of the chains relative to each other is observed. This creates a certain level of disorder with respect to the shift in chain direction. At this point, we can only speculate to what extent the differences in the structures are relevant when applying the TDEP method, as for room temperature thermal displacements, such shifts are conceivable.

Table S2: Total energy in meV/atom for the DFT functionals PBE+D3 [6–8] and vdW-DF [5], calculated for optimizations of the "Cheng's structure" and "our structure" (for details see main text).

	**PBE+D3**	**vdW-DF**
**Cheng's structure**	3.7 meV/atom	2.2 meV/atom
**Our structure**	0 meV/atom (by normalisation)	0 meV/atom (by normalisation)

Table S3: The volume for the same calculations as in Table S2. For comparison, the experimentally determined volume from Mo et al. [9] is given.

	**PBE+D3**	**vdW-DF**
**Cheng's structure**	332 Å^3	362 Å^3
**Our structure**	324 Å^3	367 Å^3
**EXPERIMENT** [9]	347 Å^3	

In addition to comparing energies in Table S2, we compare volumes in Table S3. The non-negligible difference in cell volumes is primarily caused by the choice of the DFT functional, while the choice of the starting structure for the relaxation ("our structure" vs. "Cheng's structure") has only a minor impact. This raises the question as to which of the DFT functionals produces unit cells that better fit experiments. To make this comparison, lattice constants are compared in Table S4 with experiments. Comparing the DFT results with experiments by Mo et al. and Brückner et al.[9,10] for each of the three lattice constants, it can be seen that for a_1, PBE+D3 gives a superior agreement. For a_2, vdW-DF gives better agreement, with the experimental value lying between the DFT results. For a_3, vdW-DF also agrees better with the experiment. Here, it is important to consider that the DFT-calculated values

correspond to 0 K, while the experiments (at least the one by Brückner et al. [10]) were performed at room temperature. PT exhibits thermal expansion in **x**- and **y**-direction (see Section S12). Thus, it is more reasonable that DFT underpredicts the experimental lattice constants, as is the case for the PBE+D3 functional. Overall, the agreement to experiment is good with both functionals and starting structures. In passing, we note that the "best" MTP gives very similar values to DFT, more similar than the difference caused by the functional.

*Table S4: Lattice constants a_1, a_2, and a_3 are calculated with different DFT functionals (PBE+D3 [6–8], vdW-DF [5]) and starting structures, and are compared to experiments by Mo et al. and Brückner et al.[9,10] The measurement of Brückner et al. [10] was performed at room temperature, while for the experiment of Mo et al. [9] we could not find a specified temperature. The lattice constants a_1, a_2, and a_3 correspond to the unit cell lengths in **x**-, **y**-, and **z**-direction as shown in Figure 1 of the main paper. The lattice constants with "our structure" and PBE+D3 reported here are identical to those contained in our previous publication.[1]*

	a_1 [Å]	a_2 [Å]	a_3 [Å]
**Theory**			
Our structure, PBE+D3	5.542	7.530	7.785
Cheng's structure, PBE+D3	5.639	7.561	7.786
Our structure, vdW-DF	5.840	7.900	7.856
Cheng's structure, vdW-DF	5.908	7.929	7.856
**Experiment**			
Experiment[9]	5.55	7.80	8.03
Experiment at room temperature[10]	5.33	7.79	-

In summary, the calculated thermal conductivity of Cheng et al. [4] is twice as large as our result, which we attribute to one of the following reasons: (1) different functional, (2) structure of Cheng et al. is on a saddle point of the potential energy surface (3) different methods for calculating the thermal conductivity (phono3py vs. TDEP). In the absence of experimental values, we cannot definitively assess which calculation should be preferred.

S2.2 Comparison of polyethylene's thermal conductivity with the results of Wang et al.

Wang et al. report a thermal conductivity of 237 $Wm^{-1}K^{-1}$ for the PE crystal.[11] Since they used a DFT-relaxed unit cell, similar to the DFT-relaxed unit cell in this work, we compare their result with our result obtained with the DFT-relaxed unit cell in Table S5. There, one can see that the thermal conductivity of Wang et al. significantly deviates from our result. Multiple reasons could explain this difference: Firstly, different DFT functionals were used. Wang et al. used the local-density approximation (LDA) with the optB88-vdW[12,13] correction, while we use PBE+D3 [6–8]. Secondly, different energy cut-offs were used. Wang et al. used an energy cut-off of 550 eV, while we use 700 eV. Thirdly, we used larger meshes than Wang et al. in the BTE calculation. They show a convergence test in Figure S4 of their Supplementary Information, where the thermal conductivity remains unchanged for a mesh of 40 and 50 points. This is in contrast to our convergence tests shown in Ref. [1], where we find that

larger meshes are necessary. We found that 60 points are sufficient for the BTE in the RTA, while for the full BTE, at least 80 points are necessary. It is conceivable that Wang's thermal conductivity would be increased with a larger mesh.

Table S5: Thermal conductivity of PE crystal along the chain direction at 300 K, as calculated in this study, is compared to the results of Wang et al.[11] . Values from this study are calculated with the DFT-relaxed unit cell.

	κ_{zz} from Wang et al. [11] / Wm^{-1}K^{-1}	κ_{zz} from this work / Wm^{-1}K^{-1}
**RTA**	~200	296
**full ALD-BTE**	237	398

S3 Phonon tunneling contribution to the thermal conductivity

Simoncelli et al. introduced a unified theory for thermal transport in crystals and glasses.[14] In that theory, in addition to the Peierls thermal conductivity from the Boltzmann transport equation, they proposed to add a second term, labelled coherences' thermal conductivity κ_C, that accounts for phonon tunnelling.[14] This contribution is added to the Peierls thermal conductivity κ_P to yield the total thermal conductivity κ_T. These three quantities are given in Table S6 through Table S9 for PT and PE with the DFT-relaxed and 300 K cells, respectively. For both materials and cells, the contribution from the coherences thermal conductivity is negligible in the chain direction. Therefore, and since it complicates a mode-by-mode analysis, κ_C is neglected in the main paper. In the vdW-bonded directions, however, κ_C has a much larger relative contribution. In the most extreme case, which is PE with the 300 K cell, κ_C even accounts for 38% and 33% of the total thermal conductivity in the respective vdW-bonded directions.

*Table S6: Peierls thermal conductivity, κ_P, coherences' thermal conductivity, κ_C, and total thermal conductivity, κ_T, of PT in Wm^{-1}K^{-1}. The RTA is employed, and the Brillouin zone is sampled with a 7 × 10 × 26 **q**-mesh. The DFT-relaxed cell is used, and the MTP^{phonon} is trained on data sampled at 15 K to 100 K in an NpT ensemble (as described in the Methods section of the main paper). For the definition of the different directions, see Figure 1 of the main paper.*

	x-direction / Wm^{-1}K^{-1}	y-direction / Wm^{-1}K^{-1}	z-direction / Wm^{-1}K^{-1}
$\boldsymbol{\kappa_P}$	0.496	0.428	83.2
$\boldsymbol{\kappa_C}$	0.045	0.025	1.6
$\boldsymbol{\kappa_T}$	0.541	0.453	84.8

Table S7: Same as Table S6, but with the 300 K cell and the respective MTP that is trained on data sampled at 15 K to 100 K with the unit cell fixed to the 300 K unit cell (as described in the Methods section of the main paper).

	x-direction / $Wm^{-1}K^{-1}$	y-direction / $Wm^{-1}K^{-1}$	z-direction / $Wm^{-1}K^{-1}$
κ_P	0.527	0.464	73.6
κ_C	0.037	0.022	1.9
κ_T	0.564	0.485	75.5

*Table S8: Peierls thermal conductivity, κ_P, coherences' thermal conductivity, κ_C, and total thermal conductivity, κ_T, of PE in $Wm^{-1}K^{-1}$. The RTA is employed, and the Brillouin zone is sampled with a 10 × 15 × 160 **q**-mesh. The calculations are performed using DFT with an energy cut-off of 700 eV and with the DFT-relaxed unit cell.*

	x-direction / $Wm^{-1}K^{-1}$	y-direction / $Wm^{-1}K^{-1}$	z-direction / $Wm^{-1}K^{-1}$
κ_P	0.543	0.460	306.1
κ_C	0.049	0.030	0.7
κ_T	0.592	0.490	306.8

Table S9: same as Table S8, but with the 300 K unit cell. DFT with an energy cut-off of 700 eV is used.

	x-direction / $Wm^{-1}K^{-1}$	y-direction / $Wm^{-1}K^{-1}$	z-direction / $Wm^{-1}K^{-1}$
κ_P	0.072	0.097	230.1
κ_C	0.044	0.047	0.4
κ_T	0.117	0.144	230.5

Surprisingly, for PT, the 300 K cell yields a larger thermal conductivity in vdW-bonded directions by 4% and 7%. However, it needs to be stressed here that these calculations were performed with different MTPs for the respective unit cells. When calculating the thermal conductivity with five MTPs for the DFT cell, they show a spread of 12% and 15% in the vdW-bonded directions. Thus, we attribute the apparent increase in thermal conductivity to the spread of the MTPs. In fact, when calculating the thermal conductivity for the DFT-relaxed and 300 K cell with the same MTP, we see a decrease in thermal conductivity for the 300 K cell. This is consistent with our experience that a larger unit cell leads to a reduced thermal conductivity.

On this topic, it is also worthwhile mentioning the significant decrease in the thermal conductivity perpendicular to the PE chains for the 300 K cell. Qualitatively, this result is consistently obtained also when using MTPs. The much larger change of the perpendicular thermal conductivity in PE than in PT between the two cells correlates with a significantly more pronounced thermal expansion of PE (see Section S12). A detailed discussion of the atomistic origin of this thermal conductivity increase goes beyond the scope of the present paper.

S4 Comparing ALD-BTE and AEMD results for the thermal conductivity in a vdW-bonded direction

While the focus of the main paper is on the thermal conductivity along the chain direction, we also performed AEMD simulations in one of the vdW-bonded directions of PT. In passing, we note that this requires a new finite-size extrapolation. AEMD is compared with the ALD-BTE result for the 300 K unit cell in Table S10. The AEMD result of 0.60 $Wm^{-1}K^{-1}$ agrees well with the ALD-BTE result of 0.56 $Wm^{-1}K^{-1}$. Notably, including the coherences' thermal conductivity clearly improves the agreement between the two calculations.

Table S10: Thermal conductivity of PT in the vdW-bonded **x***-direction calculated with ALD-BTE and AEMD. The ALD-BTE calculation is the same as the one reported in Table S7, with settings described there. Uncertainties in AEMD are the standard deviations (68% confidence interval) from the finite-size extrapolation.*

	**κ_{xx} / $Wm^{-1}K^{-1}$**
**ALD-BTE, κ_P**	0.527
**ALD-BTE, κ_C**	0.037
**ALD-BTE, κ_T**	0.564
**AEMD**	Zaoui fit: 0.60 ± 0.03<br>Linear fit: 0.68 ± 0.02

S5 Effect of phonon renormalization

Higher-order phonon scatterings, especially four-phonon scatterings, lead to a renormalization of phonon frequencies.[15] To study the impact of renormalization, phonon frequencies are calculated with Dynaphopy at commensurate **q**-points. Within Dynaphopy, renormalised force constants are calculated from the phonon frequencies at commensurate **q**-points, which allows to interpolate between the commensurate **q**-points.[16] The renormalization leads to a shift in phonon frequencies as shown in Figure S12 for PE. Here, we used molecular dynamics at 300 K, an MTP^{MD} and Dynaphopy to calculate the renormalised band structure with a 2 × 3 × 80 supercell and 250 ps simulation time. The longitudinal acoustic band, which is the main carrier of heat, remains unchanged upon renormalization, while other modes shift somewhat. There are a few deficiencies of the renormalised phonon band structure: Two optical bands have a "bump" at 10 THz, which appears to be an artefact of the calculation, since such bumps seem unphysical in the absence of any avoided crossings. Furthermore, to properly converge the phonon band structure calculated with Dynaphopy, larger supercells than for the phonopy calculations are required. Taking PE as an example, a 2 × 3 × 6 supercell yields a converged phonon band structure with phonopy, while for a Dynaphopy calculation, such a supercell size yields phonon bands that are wildly fluctuating and are clearly not converged. Even for the 2 × 3 × 80 supercell (see Figure S12), the transverse acoustic bands along the Γ-Z path have a slight "wave-like" dispersion, which is clearly unphysical. When increasing the supercell size even further to

4 × 6 × 80, this “wave-like” character is greatly reduced. However, the “bump” at 10 THz remains even for the larger supercell. Also, when doubling the simulation time from 250 ps to 500 ps, it does not vanish. In passing, we note that these huge supercells drastically increase the memory demand and computational cost for obtaining the phonon band structure.

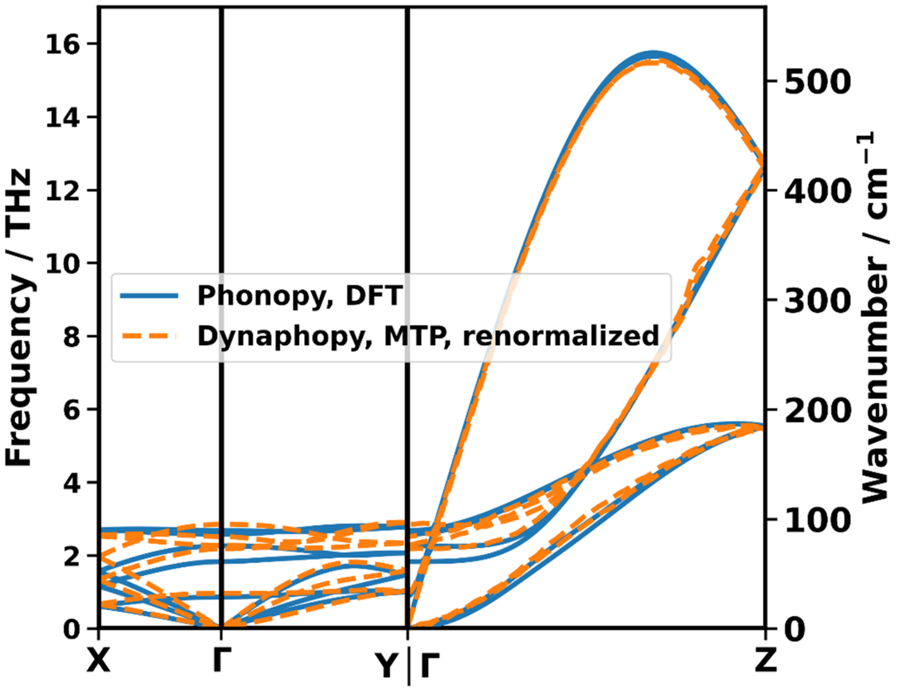

Figure S12: Phonon band structure of PE. The band structure shown as blue solid lines is calculated with finite displacements using phonopy and DFT. The calculation resulting in the phonon bands shown as dashed, orange line was performed using Dynaphopy and an MTP. The Dynaphopy calculation captures phonon renormalization at 300 K.

To investigate the influence on thermal conductivity, the renormalised second-order force constants were used to perform an ALD-BTE calculation with phono3py. The calculation was performed with a 10 × 15 × 160 **q**-mesh in the RTA, the (not renormalised) third-order force constants from phono3py, and the renormalised second-order force constants of the 2 × 3 × 80 supercell. The thermal conductivity κ_{zz} is 230 $Wm^{-1}K^{-1}$ with the unrenormalised force constants and 217 $Wm^{-1}K^{-1}$ with the renormalised force constants. We regard this 6% difference as an indication that phonon frequency renormalization does not play an important role here. This 6% difference appears especially small when we consider that the phonon band structures in the two cases have been obtained based on fundamentally different approaches, which can also contribute to the observed minor deviation. Also for PT, we observe that the renormalization typically lowers the thermal conductivity by around 5%. To make a qualitative assessment the spectral $\kappa_{z,z}$, which is the derivative of the cumulative thermal conductivity, is shown in Figure S13. It shows reasonable agreement of the ALD-BTE calculations with and without renormalization. The renormalised calculation has a somewhat smaller thermal conductivity at around 12-14 THz, while it has a larger thermal conductivity at around 2-5 THz. Since the renormalization effects are small, and considering the issues in the calculations of the renormalised band structures discussed above, phonon renormalization is not considered in any calculations except those present in the current section.

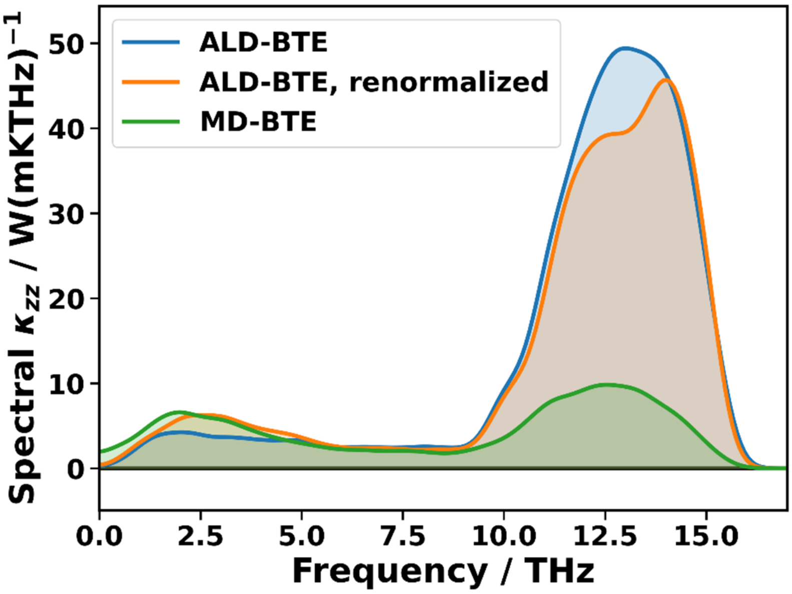

Figure S13: Spectrally resolved contributions to thermal conductivity of PE along the polymer chain, calculated as the derivative of the cumulative thermal conductivity. The ALD-BTE and MD-BTE calculations are the same as in the main paper. The "ALD-BTE, renormalized" calculation is performed with phono3py, whereby the renormalised second-order force constants from the Dynaphopy calculation are used.

S6 Ioffe-Regel limit

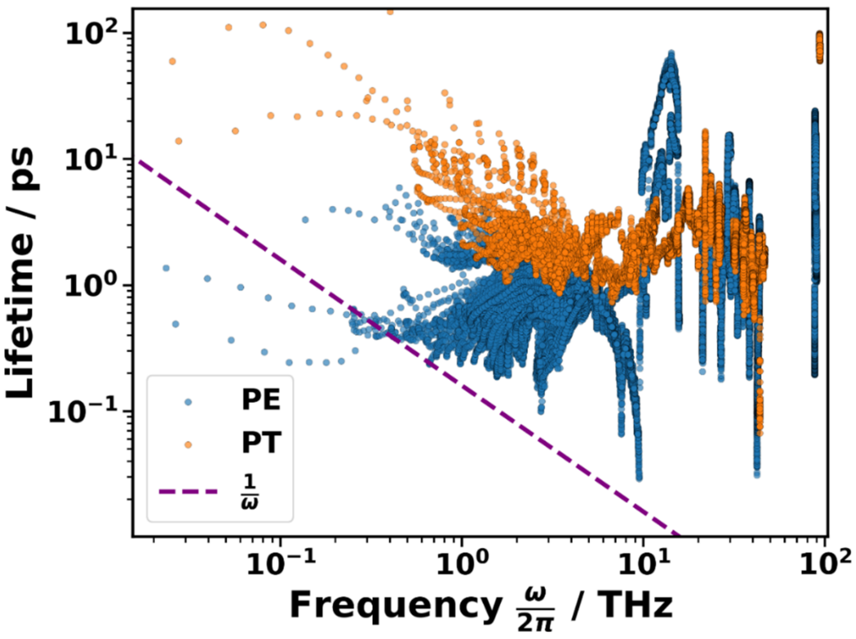

*Figure S14: Phonon lifetimes of PE and PT are plotted against the frequency. The Ioffe-Regel limit is defined by the phonon lifetimes being larger than the reciprocal angular frequency 1/ω. The **q**-meshes used in the simulations are 10 × 15 × 160 and 4 × 6 × 48 for PE and PT, respectively.*

As discussed in Ref. [17], for the BTE to be applicable, phonons must fulfil the Ioffe-Regel limit. It states that the lifetimes of phonons need to be larger than their reciprocal angular frequency. The angular frequency is given by 2π times the "usual" frequency. The latter is used throughout this manuscript, e.g., for plotting the phonon band structure and also in Figure S14. As shown in that Figure, the Ioffe-Regel limit is fulfilled for PE and PT, except for a negligible number of phonons of PE.

S7 Further details on the NEMD calculations

To initialise the NEMD run, we used the following procedure: We first equilibrated the structures at 300 K for 2.5 ps with a Langevin thermostat, then we averaged the energy for 25 ps. We ran MD until a configuration was reached whose energy equalled the average energy within a tolerance of 0.0001%. This configuration was then used as the starting point for the NEMD run. This initialization procedure minimizes effects from temperature oscillations. While we used the Langevin thermostat for the initialization of atomic positions and velocities, in the actual NEMD run, we used the Müller-Plathe method[18] in an *NVE* ensemble employing periodic boundary conditions in all directions and a time step of 0.5 fs. The definition of the regions is the same as in the seminal NEMD paper[18], to which we refer the reader for further details. In short, the NEMD setup consists of a cold region, a region with a temperature gradient, a hot region, and again a region with a temperature gradient, which is in contact with a cold region through the periodic boundary conditions.

The hot/cold region was set to be four unit cells long along the chain direction (for PE and PT). For the calculations along the chain, we found around 15 Å thickness of the simulation box to be sufficient by performing calculations with larger simulation boxes that yielded similar thermal conductivities. The 15 Å thickness of the simulation box corresponds to three and two unit cell repetitions in **y**- and **x**-direction for PE and **x**- and **y**-direction for PT. In the Müller-Plathe method, the kinetic energy of the hottest atoms in the cold region is exchanged with the coldest atoms in the hot region, which induces a temperature gradient. This Müller-Plathe energy exchange is implemented via the "fix thermal/conductivity" command in LAMMPS and was performed every 9600 (PE) or every 4800 (PT) time steps for 96 (PE) or 35 (PT) atoms. This low frequency of swaps was found to reduce the total energy drift to a negligible amount. The resulting temperature difference between the hot and cold slab was around 135 K and 180 K for the smallest and largest cell of PT, respectively. For PE, it was 64 K and 104 K for the smallest and largest cell, respectively. The total linear momenta of the hot and cold regions were fixed to zero every 100 time steps, which was achieved with the "fix momentum/chunk" command in LAMMPS. This was necessary to cancel any centre of mass drift. Simulation times strongly depend on the size of the simulation box, because for larger boxes it takes longer to reach the steady state. For each calculation, we plotted the thermal conductivity over simulation time. The simulations were performed long enough that the time averaging reduces the statistical noise sufficiently, which is typically the case for a simulation time of 1 ns after the steady state is reached.

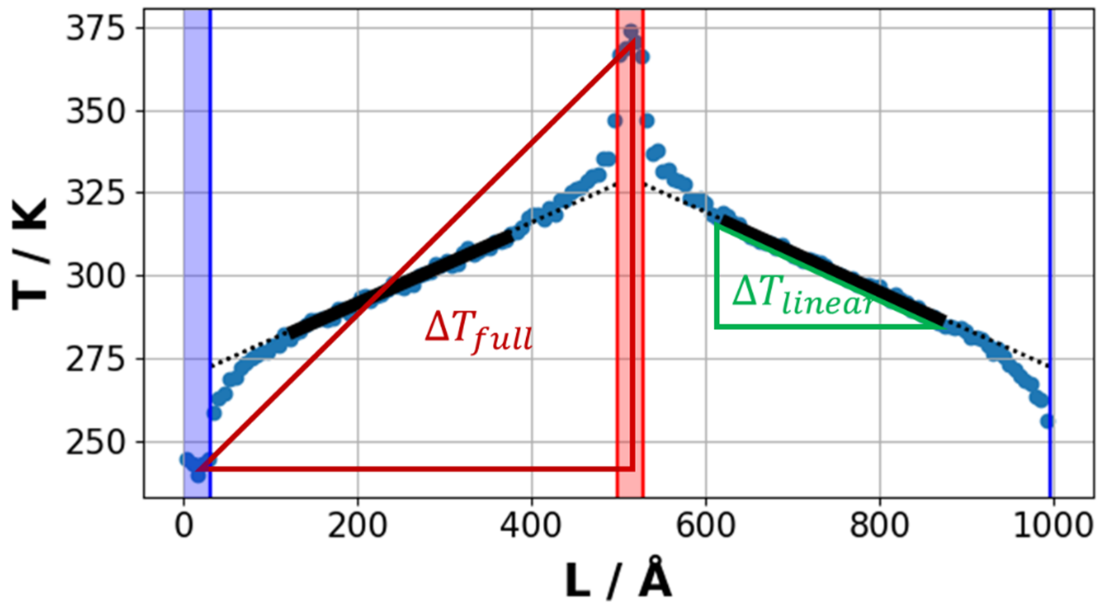

Figure S15: Typical temperature profile in a NEMD simulation, illustrating the two definitions of the temperature gradient. The cold region spans from 0 Å to 31 Å and is shaded in blue. The hot region is at 498 Å to 529 Å and is shaded in red. The linear part of the temperature profile is illustrated by a black line. Defining the temperature gradient as ΔT_{linear} takes only the linear part of the temperature profile into account. Li et al. argue that one should rather define the temperature difference as the difference between the thermostat temperatures, leading to the temperature gradient ΔT_{full}.[19]

In the literature, there exist two different definitions of the temperature gradient in NEMD, which are illustrated in Figure S15. According to one definition, one should take only the linear part of the temperature profile, which we refer to as ΔT_{linear}. However, Li et al. found that the nonlinear part should not be excluded, rather one should calculate the temperature gradient from the difference in

temperatures between the hot and cold slab.[19] Thus, we calculate this temperature gradient ΔT_{full} by dividing the temperature difference, which we obtain as the difference between the atom-averaged temperature in the hot and cold thermostat region, by half the box length. For long simulation boxes and small thermal conductivities, the two definitions converge towards each other, since the phonon scattering at the thermostat boundaries becomes small compared to the scattering in the region between the thermostats. This is the so-called diffusive regime. For example, metal organic frameworks (MOFs) have a small thermal conductivity, and some of us found that in such a case, the two definitions of the temperature gradient result only in a small difference in thermal conductivity (0.32 $Wm^{-1}K^{-1}$ with ΔT_{linear} and 0.26 $Wm^{-1}K^{-1}$ with ΔT_{full} for MOF-5).[20] However, in our case, we deal with much larger thermal conductivity and, thus, observe a strong relative contribution of the scattering at the thermostat even for the largest boxes that were simulated. Therefore, the definition of the temperature gradient makes a significant difference in our case. The numerical values can be found in Table S11. For PT, NEMD with ΔT_{full} compares very well to AEMD, MD-BTE, and ALD-BTE, while with the ΔT_{linear} definition, NEMD significantly deviates from the other methods. Also for PE, the ΔT_{full} definition yields a better agreement to AEMD and MD-BTE. Therefore, we conclude that one should take ΔT_{full} as the definition for the temperature gradient, in accordance with Li et al.[19]

Table S11: Thermal conductivity of PT and PE calculated with different methods. The purpose of this table is to compare the effect that the temperature gradient definition has on the thermal conductivity. The temperature gradient ΔT_{full} is calculated by taking the temperature difference as the average temperature difference of the thermostat regions, according to the suggestions of Li et al.[19]. ΔT_{linear} means that the temperature gradient is taken in the linear region. Uncertainties in NEMD and AEMD are the standard deviation (68% confidence interval) taken from the fitting procedure. The values for AEMD, MD-BTE, and AEMD are the same as in the main paper. Since for PE in the ALD-BTE calculation, higher-order phonon scatterings are relevant (see main paper), the comparison is not particularly helpful here and is thus omitted.

Method	κ_{zz} of PT / $Wm^{-1}K^{-1}$	κ_{zz} of PE / $Wm^{-1}K^{-1}$
**NEMD, ΔT_{full}**	Linear fit: 84 ± 9<br>2nd order polynomial fit: 98 ± 12	Linear fit: 115 ± 20<br>2nd order polynomial fit: 146 ± 22
**NEMD, ΔT_{linear}**	Linear fit: 131 ± 15<br>2nd order polynomial fit: 132 ± 27	Linear fit: 197 ± 17<br>2nd order polynomial fit: 197 ± 47§
**AEMD**	94 ± 3	127 ± 3
**MD-BTE**	98	146
**ALD-BTE**	91	-

§ When using the ΔT_{linear} definition, the calculation with the smallest unit cell is not converged with respect to the simulation time and is thus omitted in the evaluation of the 2nd order polynomial fit.

Aside from the Müller-Plathe approach, which we used throughout this work, it is also common to employ two thermostats to achieve the temperature gradient. Commonly, local thermostats, such as Langevin thermostats, are preferred. However, when using two Langevin thermostats in our MTP-based simulations of metal organic frameworks for Ref. [20], we encountered large differences between the energy that was added by one thermostat and the energy that was removed by the other

thermostat while the total energy in the system was preserved. Thus, we conclude that there must be a technical issue within the energy tallying of the thermostats in LAMMPS (Version 2nd July 2021) when used in combination with the MLIP-LAMMPS interface (Version 2).

S8 NEMD finite-size extrapolation

To account for the finite box size in the NEMD simulations, one has to perform a finite-size extrapolation, whereby one extrapolates to the bulk thermal conductivity.[21] In this extrapolation, one plots the inverse thermal conductivity over the inverse box size. Then, one typically performs a linear fit and extrapolates to an infinite box size, i.e., inverse box size of zero. [21] This extrapolation can be seen in Figure S16 for PE and PT. For PE, the used box sizes had 216, 288, 432, 576, 720, 1080, and 1440 repetitions of the unit cell along the chain direction, while perpendicular to the chains, a fixed number of unit cell repetitions is used as described in Section S7. Due to the periodic boundary conditions in the chain direction, the distance between the cold and hot thermostat regions is half the box size.[18] The 300 K primitive unit cell of PE is 2.5527 Å long, which results in thermostat distances of 275.69 Å, 367.59 Å, 551.38 Å, 735.18 Å, 918.97 Å, 1378.46 Å, and 1837.94 Å. Such large supercell lengths are necessary because of the long mean free path of the phonons along the polymer chains.

Since we used vastly different box sizes, one can see non-linear trends in Figure S16a, which are expected especially for too small box sizes. Thus, we performed the extrapolations with two approaches: As a first method, a linear fit was made through the datapoints with the largest (three) box sizes. The second method is to fit a polynomial of higher order, in this case, a 2nd order polynomial, through data points with vastly different box sizes. As shown by Sellan et al.[21], this is justified as a Taylor series expansion of the true but unknown extrapolation function. We argue that both strategies (linear and 2nd order polynomial fit) are sensible, because if one is taking box sizes that are similar in size (e.g., only the largest three box sizes), they typically lie on a straight line, and a linear fit works well. If one is trying to perform a fit for boxes of vastly different sizes, they will typically not lie on a straight line, but rather “bend down” (like in Figure S16). In that case, it is more sensible to perform a fit of higher order. Sellan et al.[21] noted that such a 2nd order fit is only sensible if multiple data points (at least three but preferably more) are available, which is the case for our PE calculation.

For PE, the aforementioned box sizes give thermal conductivities of 12.55 $Wm^{-1}K^{-1}$, 14.96 $Wm^{-1}K^{-1}$, 20.27 $Wm^{-1}K^{-1}$, 25.76 $Wm^{-1}K^{-1}$, 29.65 $Wm^{-1}K^{-1}$, 39.75 $Wm^{-1}K^{-1}$, and 47 $Wm^{-1}K^{-1}$ (using the ΔT_{full} definition of the temperature gradient, that we discuss in Section S7). Extrapolation with a 2nd order polynomial fit gives 145 $Wm^{-1}K^{-1}$ ± 22 $Wm^{-1}K^{-1}$, where the uncertainty is the standard deviation (68% confidence interval) from the fitting procedure. A linear fit through the largest three box sizes gives 115 $Wm^{-1}K^{-1}$ ± 20 $Wm^{-1}K^{-1}$. Both values agree with each other within their uncertainties. We note that the extrapolated value is significantly larger than the thermal conductivity obtained for the largest box size, which amounts to 47 $Wm^{-1}K^{-1}$. This could be seen as an indication that even larger simulation box sizes would be desirable. However, these go beyond computational capabilities, as already now the largest box contains more than 100,000 atoms, and for that NEMD simulation, the coupled equations of motion need to be solved more than 4 million times.

In analogy to PE, we also performed a finite-size extrapolation for PT. For PT, we calculated boxes with 128, 256, 512, and 1024 unit cell repetitions, which have distances between the cold and hot regions of 498 Å, 996 Å, 1992 Å, and 3984 Å. These yield thermal conductivities of 15.14 $Wm^{-1}K^{-1}$, 25.16 $Wm^{-1}K^{-1}$, 38.10 $Wm^{-1}K^{-1}$, and 56.56 $Wm^{-1}K^{-1}$. A linear fit through these datapoints gives 84 $Wm^{-1}K^{-1}$ ± 9 $Wm^{-1}K^{-1}$, while a 2nd order polynomial fit gives 98 $Wm^{-1}K^{-1}$ ± 12 $Wm^{-1}K^{-1}$ (again, with the ΔT_{full} definition of the temperature gradient explained below). These data points and the corresponding fits are shown in Figure S16b. The situation is somewhat different for PT than for PE, since the linear as well as the 2nd order polynomial fit can fit the datapoints well and also produce rather similar thermal conductivities. This is presumably due to the overall larger supercells considered for this material.

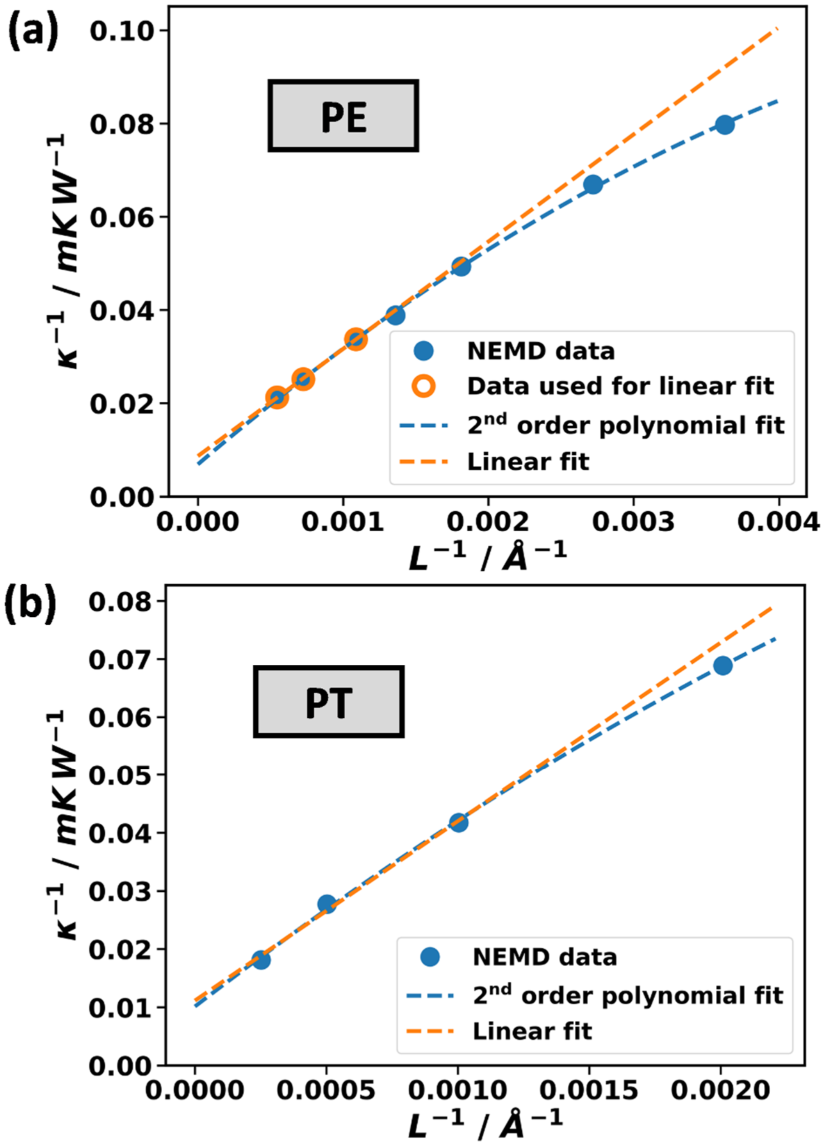

Figure S16: Finite-size extrapolation of NEMD simulation for a) PE and b) PT. A 2nd-order polynomial fit (blue dotted line) and a linear fit (orange line) are performed through the NEMD data points obtained via NEMD simulations on supercells with different lengths, L, along the polymer chains. For PE, the linear fit is only performed through the data points corresponding to the three largest unit cells

(highlighted with orange open points). For PT, both fits are performed through all the shown data points.

Still, whether a linear or a 2nd order polynomial fit should be used is unclear and, thus, constitutes a systematic error. The uncertainty from this systematic error is not accounted for in the reported uncertainties, which are solely based on the uncertainty of the respective fits. The inverse thermal conductivities for PT follow the linear fit quite well, even though the box sizes differ by up to a factor of eight. This suggests a rather well-converged extrapolation.

S9 Further details on the AEMD simulation

The AEMD setup consists of a hot and a cold simulation half. The temperature difference between the hot and cold half $\Delta T(t)$ is calculated from an atomic average over these halves. $\Delta T(t)$ is obtained by a long enough molecular dynamics run (see Section S1 for convergence tests) and is subsequently fitted by[22]

$$\Delta T(t) = \sum_{n=1}^{N_{exp}} C_n e^{-\alpha_n^2 \overline{\kappa} t} \tag{1}$$

with the number of exponentials N_{exp}, the thermal diffusivity $\overline{\kappa}$ and time t. C_n is given by

$$C_n = 8(T_1 - T_2) \frac{\left[cos\left(\frac{\alpha_n L}{2}\right) - 1\right]^2}{\alpha_n^2 L^2} \tag{2}$$

and

$$\alpha_n = \frac{2\pi n}{L} \tag{3}$$

with the summation index n indicating the order of the exponential, and the length of the cell L (comprising the hot and cold region). Formally, one ought to take N_{exp} as infinite, which is of course not feasible. Luckily, the thermal conductivity quickly converges with respect to N_{exp} as shown in Section S1 and in the literature[22]. In our case, we always take N_{exp} equal to 3 as the converged value. From the fit of $\Delta T(t)$, one obtains the thermal diffusivity $\overline{\kappa}$, from which the thermal conductivity κ can be calculated via

$$\kappa = \frac{\overline{\kappa} C_p}{V} \tag{4}$$

with the heat capacity at constant pressure C_p and the volume of the supercell V. Since we perform classical molecular dynamics (MD) simulations, phonons follow the equipartition theorem[23], and the Dulong-Petit law is valid. The Dulong-Petit law is given by

$$C_p = 3N_A k_B \tag{5}$$

with the number of atoms N_A and the Boltzmann constant k_B.

S10 AEMD finite-size extrapolation

Similar to the situation for NEMD calculations, also in AEMD, one has to correct for finite-size effects by calculating increasingly large supercells and performing a fit to an infinitely large supercell. However, the scatterings are of a different nature than in NEMD. In NEMD, phonons can scatter at the thermostat region, whereas in AEMD, there is no thermostat region. Rather, the observation of a length dependence of thermal conductivity in AEMD can be interpreted as a consequence of the extents of the hot and cold regions being smaller than the phonon mean free paths.[24] In the following, we will compare two approaches to perform the finite-size extrapolation in AEMD: a linear fit and a square root fit proposed by Zaoui et al.[24]. The resulting thermal conductivities are denoted by κ_{linear} and κ_{Zaoui}. The linear fit is defined by the function[24]

$$\frac{1}{\kappa(L)} = \frac{1}{\kappa_{linear}}\left(1+\frac{\lambda}{L}\right) \tag{6}$$

where $\kappa(L)$ are the thermal conductivity values that we obtain for the different unit cell sizes L. κ_{linear} is the bulk thermal conductivity that is obtained from the linear fit. In addition to κ_{linear}, λ is also a parameter obtained from the fit with the dimension of a length. The linear fit can be interpreted in terms of a Taylor expansion and could be refined by including more terms[24] (akin to the NEMD fit shown above). However, the linear fit cannot be physically justified in AEMD. Therefore, Zaoui et al. proposed a square root fit of the form[24]

$$\kappa(L) = \kappa_{Zaoui}\left(1-\sqrt{\frac{\Lambda_0}{L}}\right) \tag{7}$$

where the $\kappa(L)$ are again the thermal conductivities that were obtained at the unit cell sizes L. κ_{Zaoui} is the bulk thermal conductivity. Λ_0 as well as κ_{Zaoui} are obtained as a result of the fit. In the derivation of the above equation, Zaoui et al. assumed a linear dispersion of the phonon bands at low frequencies up to a frequency of ω_0. The mean free path that corresponds to that frequency is $\Lambda_0 = \Lambda(\omega_0)$. This means that the adjustable parameter Λ_0 corresponds to the lower limit in the mean free path domain, where the assumption of a linear dispersion is valid. The assumption of a linear dispersion is to some extent a limitation of the Zaoui approach. For PE and PT, this assumption is reasonably well justified because the acoustic phonons have a roughly linear dispersion and are the main carriers of heat. Zaoui et al. further assumed that the mean free path is proportional to the inverse phonon frequency squared. Since the square root fit is physically motivated, it is preferred here over the more arbitrary linear fit. Table S11 compares the thermal conductivity obtained from AEMD for the two different fit methods. For PE, supercells with 432, 720, 1080, 1440, and 2160 repetitions of the primitive unit cell in chain direction were calculated, which yielded thermal conductivities of 44.3 $Wm^{-1}K^{-1}$, 61.1 $Wm^{-1}K^{-1}$, 73.0 $Wm^{-1}K^{-1}$, 80.6 $Wm^{-1}K^{-1}$, and 91.9 $Wm^{-1}K^{-1}$ with simulation times given in Section S1.4. A square root fit and a linear fit give very similar results of 127 $Wm^{-1}K^{-1}$ ± 3 $Wm^{-1}K^{-1}$ and 125 $Wm^{-1}K^{-1}$ ± 3 $Wm^{-1}K^{-1}$, respectively (with the standard deviations from the fits as uncertainties). For PT, supercell sizes with repetitions of 128, 192, 256, 384, and 512 were calculated. These give thermal conductivities of 27.2 $Wm^{-1}K^{-1}$, 37.1 $Wm^{-1}K^{-1}$, 45.8 $Wm^{-1}K^{-1}$, 56.6 $Wm^{-1}K^{-1}$, and 59.6 $Wm^{-1}K^{-1}$. The square root fit yields 94 $Wm^{-1}K^{-1}$ ± 3 $Wm^{-1}K^{-1}$ and the linear fit 112 $Wm^{-1}K^{-1}$ ± 13 $Wm^{-1}K^{-1}$. Both fits are consistent with each other within two standard deviations. The linear fit has a much larger uncertainty of 13 $Wm^{-1}K^{-1}$, which comes about because the linear fit is not able to fit the data point for the largest cell size well (see Figure S17b). Accurately fitting the calculations with large unit cells is more important than accurately

fitting the calculations with small unit cells. The square root fit performs much better in that regard. For this reason and since it is physically motivated, the square root fit is preferred. Thus, we only give the result for the square root fit in the main paper.

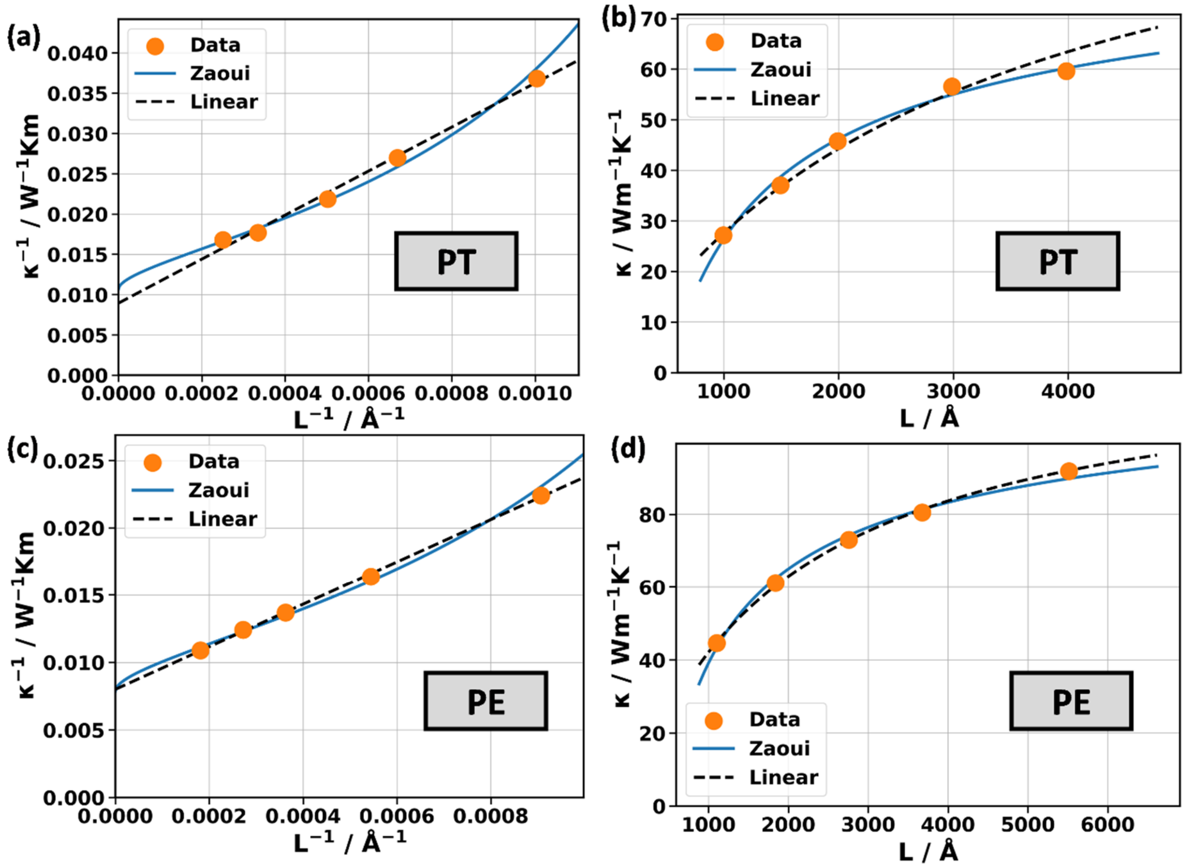

Figure S17: AEMD finite-size extrapolation for PT plotted for inverse (a) and linear axes (b). (c) and (d) contain equivalent plots for PE. "Zaoui" refers to the square root fit suggested by Zaoui et al.[24], while "Linear" refers to a linear fit of inverse quantities.

S11 MTPs for NEMD and AEMD simulations

As discussed in the Methods section of the main paper, MTPs parametrised in a somewhat different way from the procedure suggested in Ref. [1] were used for NEMD and AEMD simulations. When we started working with MTPs, we tested different settings for the MTPs to see which worked best. This resulted in MTPs that were trained on a mix of different training data, as described below. These "old" MTPs were used to perform the expensive NEMD and AEMD simulations during the testing phase of the Vienna Scientific Cluster 5 (VSC-5), during which computing hours were not billed. Later, we trained MTPs with more consistent settings, as described in Ref. [1] and in the main paper. Ideally, we would have repeated the NEMD and AEMD simulations with these new "consistent" MTPs. However, this would have been exceedingly expensive, as described in the main paper. Instead of repeating the NEMD and AEMD calculations, including the complete finite-size extrapolation, we only repeated the AEMD simulation with relatively small unit cells and show that the "consistent" MTPs give similar

thermal conductivities as the “old” MTPs. This is done in Table S12 for PT and Table S13 for PE. To put this comparison into perspective, it is useful to consider that the spread between thermal conductivity values obtained with MTP^{MD}s fitted with different initializations is around 8% and 5% for the smallest considered supercell of PT and PE, respectively (see Section S16 for details). Here, the difference in thermal conductivity between the “old” MTPs and “consistent” MTPs for the same (smallest) supercell is 8% and 4% for PT and PE, respectively. The extrapolated thermal conductivity varies by 1% and 14% between the two types of MTPs for PT and PE, respectively. Since the difference between the types of MTPs is very similar to the spread of the MTP^{MD}s, we argue that the “old” MTPs and “consistent” MTPs can be regarded as essentially equivalent.

Regarding the parametrization of the “old” MTPs: For PT, it has a level of 16. Its training data consists of 380 structures sampled with a temperature ramp from 15 K to 500 K and 169 structures sampled at 15 K during an MD run with constant temperature. The “old” MTP of PE is a level 22 MTP with 46 structures sampled at 15 K at constant temperature, 168 structures sampled with a temperature ramp from 15 K to 300 K, and 177 structures sampled at the constant temperature 400 K. For PE, we gave more weight to configurations close to equilibrium by setting the “scale-by-force” keyword to 0.7 in MLIP. As written in the main paper, the “consistent” MTPs are level 22, have training data from 15 K to 500 K, and are trained without using the “scale-by-force” keyword. In passing, we note that this serves as a test for the level, since the level 16 “old” MTP and the level 22 “consistent data” MTP yield very similar thermal conductivities.

Table S12: Thermal conductivity of PT calculated with AEMD for different MTPs and different unit cell repetitions along the chain direction. The bulk value is obtained by an extrapolation with the square root fit suggested by Zaoui et al.[24].

unit cell repetitions	κ_{ZZ} with “old” MTP / $Wm^{-1}K^{-1}$	κ_{ZZ} with “consistent” MTP / $Wm^{-1}K^{-1}$
**128**	27.2	29.3
**192**	37.1	41.7
**256**	45.8	46.8
**bulk**	89.2	90.2

Table S13: Thermal conductivity of PE calculated with AEMD for different MTPs and different unit cell repetitions along the chain direction. The bulk value is obtained by an extrapolation with the square root fit suggested by Zaoui et al.[24].

unit cell repetitions	κ_{ZZ} with “old” MTP / $Wm^{-1}K^{-1}$	κ_{ZZ} with “consistent” MTP / $Wm^{-1}K^{-1}$
**432**	43.2	44.9
**720**	61.1	62.3
**1080**	73.0	79.6
**bulk**	120.1	136.7

S12 DFT-relaxed and 300 K unit cells

The unit cell length of the DFT-relaxed and 300 K unit cells, which were used for the calculations in the main paper, are given in Table S14. The agreement between the DFT- and MTP-relaxed unit cells is good, as shown in much more detail in Ref. [1]. The thermal expansion in vdW-bonded directions is 5% and 1.3% for PE, while it is only 0.9% and 1.0% for PT. This very large thermal expansion of PE leads to significant changes in the thermal conductivity, as discussed in the main paper. In stark contrast to the vdW-bonded directions, the thermal expansion in the chain direction is very small and negative.

Table S14: Lattice parameters a_1, a_2, and a_3 of the 0 K and 300 K unit cells of PE and PT (see Figure 1 for definitions of the directions). 0 K unit cells are the ones that are relaxed with DFT and the MTP^{phonon} (see Ref. [1] for more details). 300 K unit cells are obtained by performing MD with the MTP (see Methods section of main paper for details).

**Temperature [K]**	**a_1 [Å]**	**a_2 [Å]**	**a_3 [Å]**
	**Polyethylene**		
0 K, DFT	7.074	4.853	2.554
0 K, MTP^{phonon}	7.062	4.847	2.554
300 K, MTP	7.445	4.918	2.553
	**Polythiophene**		
0 K, DFT	7.530	5.542	7.785
0 K, MTP^{phonon}	7.467	5.508	7.782
300 K, MTP	7.600	5.600	7.780

S13 Complication with the Dynaphopy fit for PT for one of the considered q-points

In Figure 3d of the main paper, the thermal conductivity contribution of the phonons along the shown path is calculated with MD-BTE. The MD-BTE calculations are performed with Dynaphopy, which projects the velocities onto phonon eigenvectors (see Ref. [16] for more details). The result is a power spectrum for each phonon mode and wave vector **q**. Ideally, this power spectrum should contain one Lorentzian peak, which is fitted within Dynaphopy to obtain the phonon lifetime. This is also the case for nearly all considered **q**-points; however, for the longitudinal acoustic phonon at the considered **q**-point closest to the Γ-point, the power spectrum displays two peaks, as shown in Figure S18. This is, insofar, a problem, as it puts the Lorentzian fit for this mode at this wave vector into question. Notably, in the phonopy calculation, there is only a band at around 0.4 THz, but not at 0.38 THz. The shape of this power spectrum also does not change fundamentally, even when the simulation time is increased from 1 ns to 2 ns and the resolution is decreased from 0.004 THz to 0.002 THz. We note that for all other power spectra that we looked at, there is only one peak. These considerations lead us to the conclusion that the MD-BTE calculation might be less reliable for this particular phonon mode at this particular wave vector, but is valid for all other modes and wave vectors. Considering the small volume in reciprocal space that the concerned **q**-point is associated with, the described complication is expected to have no impact on the calculated thermal conductivity.

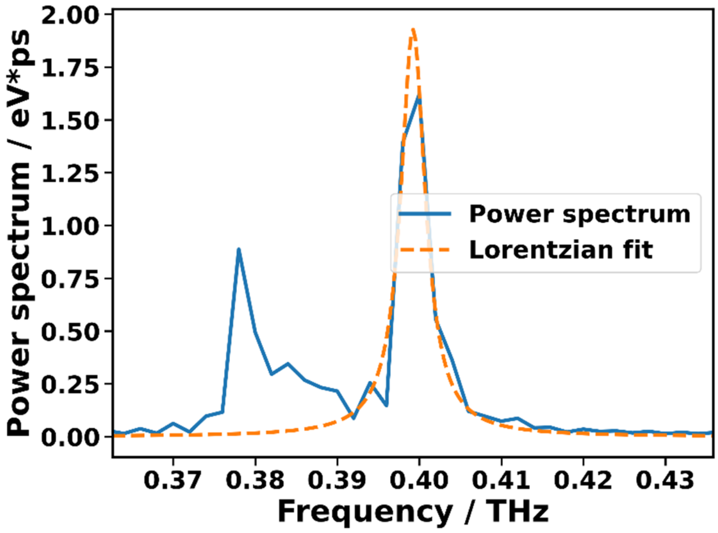

Figure S18: The Dynaphopy calculation of PT yielded this power spectrum for the longitudinal acoustic mode closest to the Γ-point. The displayed Lorentzian fit was performed with an in-house Python script that takes the lifetime from the Dynaphopy calculation and fits the peak position and peak height. The simulation time is 2 ns, and the resolution is 0.002 THz.

S14 Frequency-resolved thermal conductivity with heat capacity according to equipartition and Bose-Einstein statistics

To illustrate what difference it makes whether the Bose-Einstein mode heat capacity C_{BE} or the equipartition mode heat capacity C_{EQ} is used, the MD-BTE calculations are evaluated with both heat capacities, and the results are shown in Figure S19. For PT, where the major contributions to heat transport come from low-frequency phonons, the choice of the mode heat capacity has only a small effect. The situation is different for PE, where, due to high-frequency phonons, the choice of the heat capacity has a larger effect than for PT.

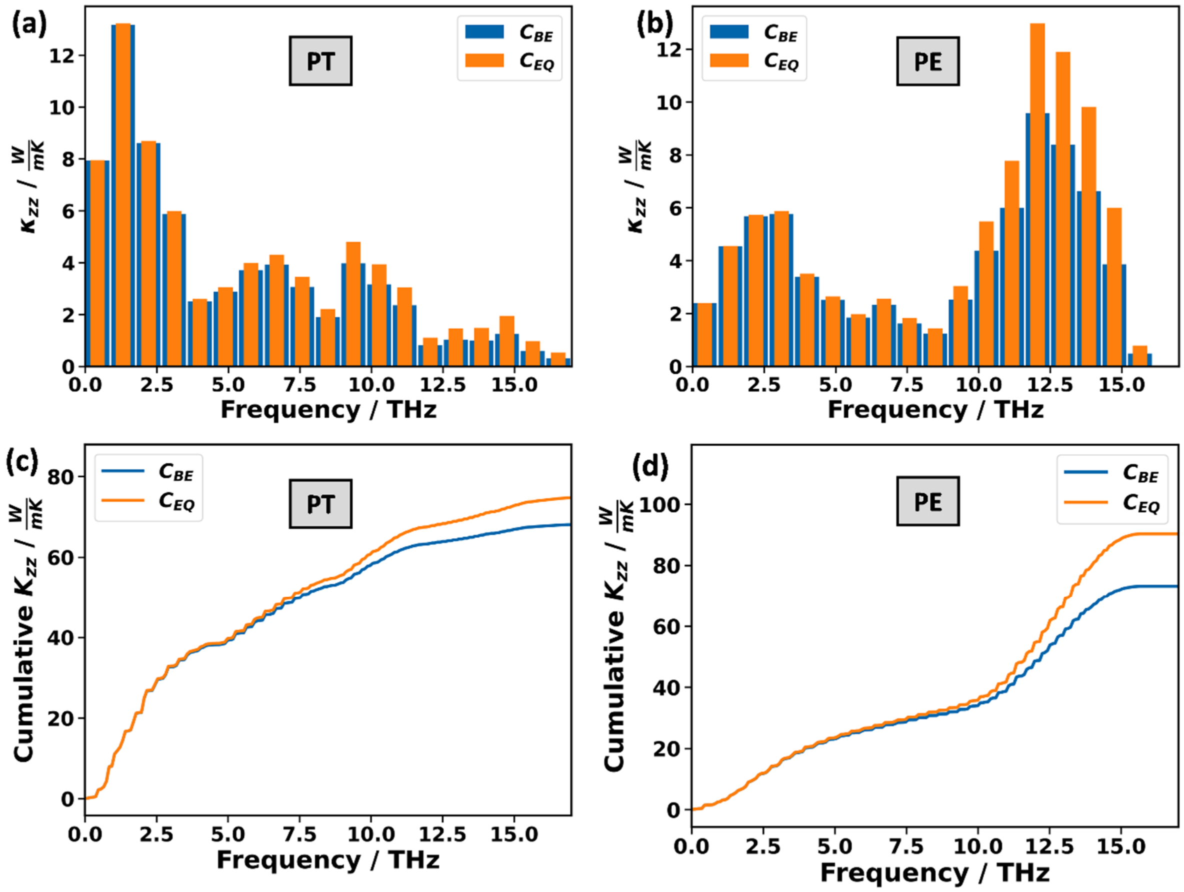

Figure S19: Frequency-resolved MD-BTE thermal conductivities calculated with C_{BE} and C_{EQ} assuming a temperature of 300 K for (a) PT and (b) PE. The corresponding cumulative thermal conductivities are shown in panels (c) and (d) for PT and PE, respectively. As for all MD-BTE calculations, the 300 K unit cell is used.

S15 Lifetimes of PT calculated with MD-BTE

In the main paper, the lifetimes of PT along the Γ-Z path are given for the ALD-BTE calculation. Here, a respective plot for the MD-BTE calculation is provided in Figure S20b. For comparison, the respective ALD-BTE calculation is reprinted from the main paper in Figure S20a. This comparison shows that the phonon lifetimes calculated with ALD-BTE and MD-BTE agree rather well with each other. The MD-BTE calculation is performed for 4 ns. Even for this rather long simulation time, the datapoints with the largest lifetimes are somewhat noisy. A calculation with an even longer simulation time could remedy this, but since this would not qualitatively change the result and considering the rather high computational cost, we refrained from performing this calculation.

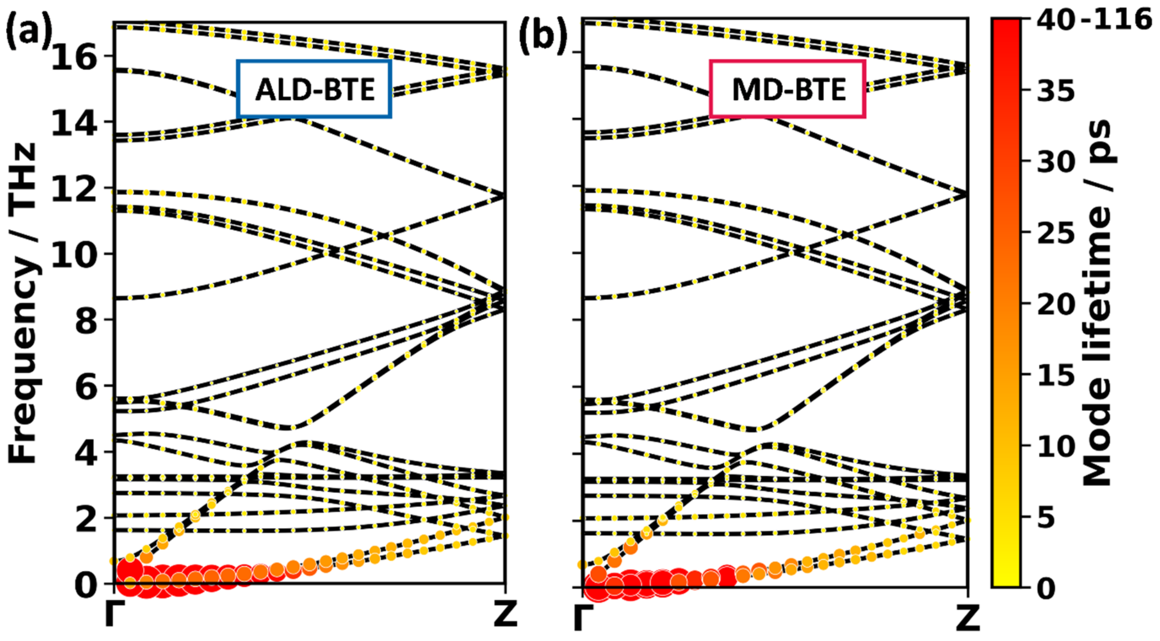

Figure S20: Lifetimes of PT along the Γ-Z-path calculated with (a) ALD-BTE and (b) MD-BTE. The ALD-BTE plot is the same as in the main paper.

S16 Statistical noise in AEMD simulations and uncertainty of MTPs in AEMD

When an AEMD simulation is restarted with a different random seed for initializing the velocities, the resulting thermal conductivity is different due to statistical noise. Typically, we observe that this noise is smaller for larger simulation cells. This is sensible because the thermal conductivity is effectively evaluated as an "average" over the atoms, and a larger number of atoms thus reduces the statistical noise. Therefore, to estimate an "upper limit" of the statistical noise and to save computational resources, we investigated the noise with the smallest supercell, which was used in AEMD simulations. For PT, this cell contains 21,504 atoms with 128 unit-cell repetitions in the chain direction, while for PE, it has 31,104 atoms and 432 unit-cell repetitions along the chain. Five simulations with different random seeds yield the thermal conductivities listed in Table S15. For both materials, the standard deviation is 5% of the mean value, which we regard as acceptably small. The statistical noise could be reduced by performing each calculation multiple times and taking the average over these calculations. However, since we estimate that the statistical noise is 5% for the smallest supercell and even less for larger cells, we argue that repeating the calculations is not necessary in our case, especially considering the high computational cost that would be associated with repeating the calculations multiple times.

Table S15: Thermal conductivity with different random seeds for initialization of the velocities in AEMD simulations of the smallest studied supercells. These supercells have the dimensions 2 × 3 × 128 and 2 × 3 × 432 for PT and PE, respectively. Other simulation parameters, including the used MTP, are kept fixed for each material.

**Index of seed**	**Thermal conductivity κ_{zz} of PT / Wm^{-1}K^{-1}**	**Thermal conductivity κ_{zz} of PE / Wm^{-1}K^{-1}**
**1**	25.87	44.9
**2**	28.11	42.5
**3**	29.33	41.7
**4**	29.62	47.6
**5**	29.67	46.2
**Mean**	**28.52**	**44.6**
**Standard deviation**	**1.44**	**2.4**

Having determined the statistical noise inherent to the AEMD simulation, we next consider the uncertainty stemming from the MTPs. For this purpose, five MTPs are trained with differently initialised parameters. These MTPs are parametrised according to the MTP^{MD} scheme, as described in the Methods section of the main paper. They yield the thermal conductivities reported in Table S16. For PT, the statistical spread caused by the MTP is around 50% larger than the statistical uncertainty of the differently initialised AEMD simulations discussed above (see Table S15). Still, the overall spread is rather minor considering the small size of the supercells. For PE, the calculated spread due to different MTPs is essentially the same as the statistical noise caused by the AEMD seeds and amounts to around 5%.

Table S16: Thermal conductivity is calculated using AEMD with five different MTPs for PT (second column) and PE (third column). For PT, the initial seed is set to seed #3 (see Table S15), therefore, the calculation with the third index is identical to the one in Table S15. The supercells have the dimensions 2 × 3 × 128 and 2 × 3 × 432 for PT and PE, respectively.

**MTP index**	**Thermal conductivity κ_{zz} of PT / Wm^{-1}K^{-1}**	**Thermal conductivity κ_{zz} of PE / Wm^{-1}K^{-1}**
**1**	28.99	46.2
**2**	32.27	44.9
**3**	29.33	47.1
**4**	25.77	41.5
**5**	27.40	47.5
**Mean**	**28.76**	**45.5**
**Standard deviation**	**2.17**	**2.4**

S17 Uncertainty of the MTPs in MD-BTE simulations of PE

Considering the complications encountered for the ALD-BTE calculations of PE when using MTP^{phonon}s and the 300 K unit cell (see main manuscript), it is worthwhile testing whether similar problems occur for MD-BTE simulations. In fact, in the MD-BTE simulation, a much more benign behaviour of (in this case, MTP^{MD} potentials) was observed: MD-BTE calculations of five distinct MTPs yield 73.3 Wm^{-1}K^{-1}, 73.4 Wm^{-1}K^{-1}, 80.3 Wm^{-1}K^{-1}, 65.9 Wm^{-1}K^{-1}, and 81.2 Wm^{-1}K^{-1}. This gives a mean of 74.8 Wm^{-1}K^{-1} and a standard deviation of 5.6 Wm^{-1}K^{-1}, which corresponds to 7% of the mean value. This uncertainty is substantially smaller than for the ALD-BTE simulations, as argued in the main paper. The simulations are performed with a 2 × 3 × 80 supercell, 1 ns simulation time, and resolution of 0.004 THz. Here, the supercell is reduced from a 2 × 3 × 160 supercell, as was used in the main paper, to 2 × 3 × 80 to save computational resources, while still employing essentially converged settings.

Figure S21 shows that not only the finally obtained thermal conductivities, but also the general trends for the mode contributions to the thermal conductivity are consistent between the different MTPs. In passing, we note that based on the smallest force error for the verification set, MTP^{MD} #1 was chosen as the "best" MTP^{MD}.

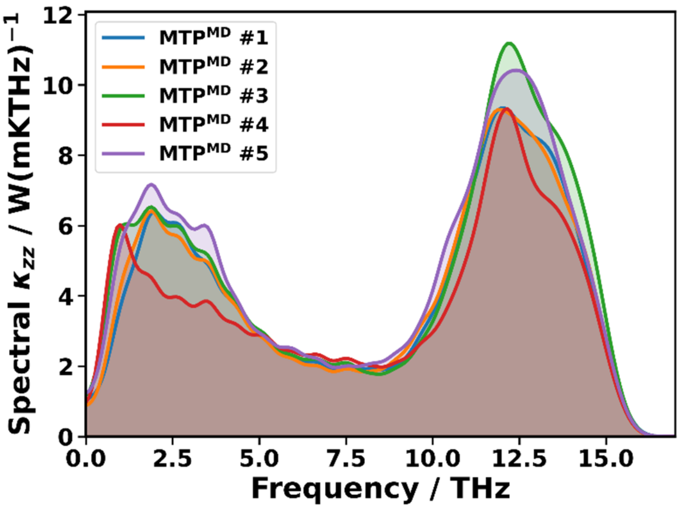

Figure S21: Spectrally resolved contributions to thermal conductivity of PE calculated with MD-BTE employing five distinct MTP^{MD}s. MTP^{MD} #1 is the "best" MTP that was used throughout the main paper.

S18 Thermal conductivities of additional MTPs with ALD-BTE

Typically, we parametrised five MTPs for each configuration (i.e., type of unit cell, material). In the main paper, we reported the "best" out of these and the mean values for the MTP. Here, we report the individual thermal conductivities. The number of negative eigenvalues of the collision matrix is reported in brackets and is discussed in Section S1.6.

Table S17: Thermal conductivities of PE in $Wm^{-1}K^{-1}$ for the DFT-relaxed unit cell. The thermal conductivities calculated with five differently initialised MTP^{phonon}s are given. MTP #4 is the "best" MTP (see Method Section of main paper for the definition of "best"). The mean value of five differently initialised MTPs is reported with the standard deviation in square brackets. The calculation of the mean for the full BTE calculation is omitted for the 4 × 6 × 120 ***q****-mesh because of a negative thermal conductivity. This negative thermal conductivity value is underlined. The number of negative eigenvalues of the collision matrix is given in round brackets (see Section S1.6 for more details). The thermal conductivities that are reported in the main paper are highlighted in bold.*

	**MTP index**	**q-mesh 4 × 6 × 120**	**q-mesh 4 × 6 × 160**
RTA	1	229	230
	2	284	281
	3	327	324
	4, "best"	291	**295**
	5	282	286
	mean	283 [± 35]	**284 [± 34]**
full BTE	1	315 (0)	313 (0)
	2	398 (0)	385 (1)
	3	-159 (1)	430 (1)
	4, "best"	435 (2)	**408** (1)
	5	408 (1)	404 (2)
	mean	-	**388 [± 40]**

Table S18: Thermal conductivities of PE are reported similarly to Table S17, except that for these calculations, the 300 K unit cell is used. Unreasonable thermal conductivities are underlined.

	**MTP index**	**q-mesh 4 × 6 × 120**	**q-mesh 4 × 6 × 160**	**q-mesh 4 × 6 × 320**
RTA	1	369	363	372
	2	339	335	341
	3, "best"	265	**263**	265
	4	304	304	307
	5	352	357	359
	mean	336 [± 42]	**324 [± 41]**	329 [± 43]
full BTE	1	484 (1)	491 (2)	642 (1)
	2	386 (1)	2527 (1)	506 (2)
	3, "best"	303 (1)	**393** (0)	416 (1)
	4	434 (0)	325 (1)	469 (1)
	5	884 (0)	415 (2)	545 (1)

Table S19: Thermal conductivities of PT in $Wm^{-1}K^{-1}$ for the DFT-relaxed unit cell. The notation is the same as in Table S17.

	MTP index	q-mesh 4 × 6 × 48
RTA	1	77.0
	2, “best”	**83.9**
	3	88.0
	4	73.1
	5	81.7
	mean	**80.8 [± 5.8]**
full BTE	1	93.5 (1)
	2, “best”	**98.4** (0)
	3	104.8 (1)
	4	89.1 (0)
	5	95.9 (0)
	mean	**96.3 [± 5.8]**

Table S20: Thermal conductivities of PT in $Wm^{-1}K^{-1}$ for the 300 K unit cell. The notation is the same as in Table S17.

	MTP index	q-mesh 4 × 6 × 48
RTA	1	73.4
	2, “best”	**78.5**
	3	71.0
	4	66.1
	5	64.9
	mean	**70.8 [± 5.6]**
full BTE	1	88.1 (1)
	2, “best”	**91.9** (1)
	3	88.3 (1)
	4	75.8 (1)
	5	79.7 (1)
	mean	**84.7 [± 6.7]**

S19 Outlier MTP for the DFT-relaxed unit cell of PE

As listed in the previous section, the MTPs for the DFT unit cell of PE yield 230 $Wm^{-1}K^{-1}$, 281 $Wm^{-1}K^{-1}$, 324 $Wm^{-1}K^{-1}$, 295 $Wm^{-1}K^{-1,}$ and 286 $Wm^{-1}K^{-1}$ in the RTA, generally comparing well to the DFT result of 296 $Wm^{-1}K^{-1}$. We regard the MTP that gives 230 $Wm^{-1}K^{-1}$ as an outlier, which raises the question whether this outlier MTP yields results that are also qualitatively at variance with the DFT results. In Figure S22, the spectral thermal conductivity is plotted for these MTPs alongside the DFT result. The outlier MTP is the one labelled “MTP #1”. It is the one that is deviating the most from DFT, but it still

reproduces the overall qualitative trend that the largest contributions to the thermal conductivity, when neglecting higher-order scattering processes, stem from phonons between 11 THz and 16 THz.

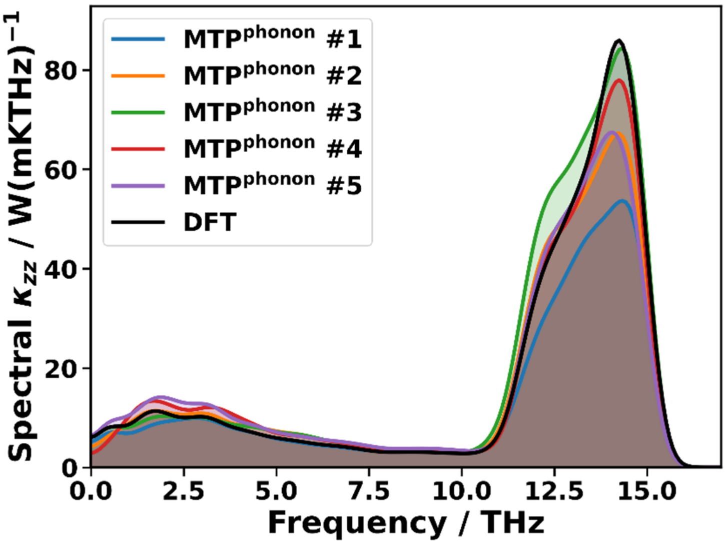

Figure S22: The spectrally resolved contributions to the thermal conductivity of PE are calculated with the DFT-relaxed unit cell using five MTP^{phonon}s (coloured lines) and using DFT (black line). "MTP^{phonon} #4" is the "best" MTP.

S20 References

1. Reicht, L., Legenstein, L., Wieser, S. & Zojer, E. Designing Accurate Moment Tensor Potentials for Phonon-Related Properties of Crystalline Polymers. *Int J Mol Sci* (2024) doi:10.3390/molecules29163724.

2. Wu, X. *et al.* Correcting force error-induced underestimation of lattice thermal conductivity in machine learning molecular dynamics. *Journal of Chemical Physics* **161**, (2024).

3. Togo, A. First-principles Phonon Calculations with Phonopy and Phono3py. *J Physical Soc Japan* **92**, (2023).

4. Cheng, P., Shulumba, N. & Minnich, A. J. Thermal transport and phonon focusing in complex molecular crystals: Ab initio study of polythiophene. *Phys Rev B* **100**, 94306 (2019).

5. Dion, M., Rydberg, H., Schröder, E., Langreth, D. C. & Lundqvist, B. I. Van der Waals density functional for general geometries. *Phys Rev Lett* **92**, 246401 (2004).

6. Perdew, J. P., Burke, K. & Ernzerhof, M. Generalized gradient approximation made simple. *Phys Rev Lett* **77**, 3865–3868 (1996).

7. Grimme, S., Ehrlich, S. & Goerigk, L. Effect of the Damping Function in Dispersion Corrected Density Functional Theory. (2010) doi:10.1002/jcc.21759.

8. Grimme, S., Antony, J., Ehrlich, S. & Krieg, H. A consistent and accurate ab initio parametrization of density functional dispersion correction (DFT-D) for the 94 elements H-Pu. *Journal of Chemical Physics* **132**, (2010).

9. Mo, Z. *et al.* X-ray Scattering from Polythiophene: Crystallinity and Crystallographic Structure. *Macromolecules* **18**, 1972–1977 (1985).

10. Brückner, S. & Porzio, W. The structure of neutral polythiophene. An application of the Rietveld method. *Die Makromolekulare Chemie* **189**, 961–967 (1988).

11. Wang, X., Kaviany, M. & Huang, B. Phonon coupling and transport in individual polyethylene chains: A comparison study with the bulk crystal. *Nanoscale* **9**, 18022–18031 (2017).

12. Klimeš, J., Bowler, D. R. & Michaelides, A. Chemical accuracy for the van der Waals density functional. *Journal of Physics Condensed Matter* **22**, (2010).

13. Klimeš, J., Bowler, D. R. & Michaelides, A. Van der Waals density functionals applied to solids. *Phys Rev B Condens Matter Mater Phys* **83**, 1–13 (2011).

14. Simoncelli, M., Marzari, N. & Mauri, F. Unified theory of thermal transport in crystals and glasses. *Nat Phys* **15**, 809–813 (2019).

15. Li, Z., Xia, Y. & Wolverton, C. First-principles calculations of lattice thermal conductivity in Tl 3 VSe 4 : Uncertainties from different approaches of force constants. **184307**, 1–10 (2023).

16. Carreras, A., Togo, A. & Tanaka, I. DynaPhoPy: A code for extracting phonon quasiparticles from molecular dynamics simulations. *Comput Phys Commun* **221**, 221–234 (2017).

17. Simoncelli, M., Marzari, N. & Mauri, F. Wigner Formulation of Thermal Transport in Solids. *Phys Rev X* **12**, 41011 (2022).

18. Müller-Plathe, F. A simple nonequilibrium molecular dynamics method for calculating the thermal conductivity. *Journal of Chemical Physics* **106**, 6082–6085 (1997).

19. Li, Z. *et al.* Influence of thermostatting on nonequilibrium molecular dynamics simulations of heat conduction in solids. *Journal of Chemical Physics* **151**, (2019).

20. Wieser, S. & Zojer, E. Machine learned force-fields for an Ab-initio quality description of metal-organic frameworks. *NPJ Comput Mater* **10**, (2024).

21. Sellan, D. P., Landry, E. S., Turney, J. E., McGaughey, A. J. H. & Amon, C. H. Size effects in molecular dynamics thermal conductivity predictions. *Phys Rev B Condens Matter Mater Phys* **81**, 1–10 (2010).

22. Melis, C., Dettori, R., Vandermeulen, S. & Colombo, L. Calculating thermal conductivity in a transient conduction regime: theory and implementation. *European Physical Journal B* **87**, 1–9 (2014).

23. Puligheddu, M., Xia, Y., Chan, M. & Galli, G. Computational prediction of lattice thermal conductivity: A comparison of molecular dynamics and Boltzmann transport approaches. *Phys Rev Mater* **3**, 1–11 (2019).

24. Zaoui, H., Palla, P. L., Cleri, F. & Lampin, E. Length dependence of thermal conductivity by approach-to-equilibrium molecular dynamics. *Phys Rev B* **94**, 054304 (2016).
